# Revision of Chinese *Phorocardius* species (Coleoptera, Elateridae, Cardiophorinae)

**DOI:** 10.3897/zookeys.993.53805

**Published:** 2020-11-16

**Authors:** Yongying Ruan, Hume B. Douglas, Lu Qiu, Xiaoqin Chen, Shihong Jiang

**Affiliations:** 1 School of Applied Chemistry and Biological Technology, Shenzhen Polytechnic, Shenzhen, Guangdong 518055, China School of Applied Chemistry and Biological Technology Shenzhen China; 2 Canadian National Collection of Insects, Arachnids and Nematodes, Agriculture and Agri-Food Canada, 960 Carling Ave., Ottawa, Ontario, K1A 0C6, Canada Canadian National Collection of Insects Ottawa Canada; 3 Institute of Entomology, College of Plant Protection, Southwest University, Beibei, Chongqing 400716, China Southwest University Chongqing China

**Keywords:** *Cardiophorus*, checklist, click beetles, *Displatynychus*, diversity, elaterid, new species, review

## Abstract

The Chinese species of *Phorocardius* Fleutiaux, 1931 have been studied and six species are described as new: *P.alterlineatus* Ruan & Douglas, **sp. nov.**; *P.flavistriolatus* Ruan & Douglas, **sp. nov.**; *P.minutus* Ruan & Douglas, **sp. nov.**; *P.rufiposterus* Ruan & Douglas, **sp. nov.**; *P.yunnanensis* Ruan & Douglas, **sp. nov.**; and *P.zhiweii* Ruan, Douglas & Qiu, **sp. nov.** Lectotypes are designated for *Cardiophoruscomptus* Candèze, 1860, *Cardiophoruscontemptus* Candèze, 1860, *Phorocardiusmagnus* Fleutiaux, 1931, and *Cardiophorusmanuleatus* Candèze, 1888. The holotype is identified for *Cardiophorusyanagiharae* Miwa, 1927. *Phorocardiusflorentini* (Fleutiaux, 1895) and *P.manuleatus* (Candèze, 1888) are newly reported from China; *P.comptus* (Candèze, 1860) is excluded from the Chinese fauna. A key to the 11 *Phorocardius* species known from China is given. *Phorocardius* is newly recorded from deep within the Palearctic Region. The procoxal cavities of *P.rufiposterus* Ruan & Douglas, sp. nov. are closed, which is different from all other species of *Phorocardius*. An annotated checklist of the 21 *Phorocardius* species of the world is provided. Additionally, *Phorocardiuscontemptus* (Candèze, 1860), **comb. nov.** is transferred from *Cardiophorus* to *Phorocardius*; four species are transferred from *Phorocardius* to *Displatynychus*: *Displatynychusbombycinus* (Candèze, 1895), **comb. nov.**, *Displatynychuspakistanicus* (Platia & Ahmed, 2016), **comb. nov.**, *Displatynychussobrinus* (Laporte, 1840), **comb. nov.**, and *Displatynychustibialis* (Platia & Ahmed, 2016), **comb. nov.**

## Introduction

*Phorocardius* Fleutiaux, 1931 is a small Asian genus of elaterids with 15 species known previously (Douglas et al. 2018), placed in the subfamily Cardiophorinae Candèze, 1859 (Fleutiaux 1931; Douglas 2017; Douglas et al. 2018). Fleutiaux (1931) established *Phorocardius* and its two subgenera for Cardiophorinae with tarsal claws bidentate near their apices. Subsequently, Fleutiaux (1947) revised eight species from French Indo-China (Vietnam, Laos, and Cambodia). Since 1947, only a few new studies have been published: Platia (2015) and Platia and Ahmed (2016) described new species from Pakistan and Maldives respectively; and Douglas (2017) redefined the genus based on a phylogeny using adult morphological characters.

Previously, *Phorocardius* included a second subgenus: *Diocarphus* Fleutiaux, 1947. *Diocarphus* was recognized by the reduced ventral apex of the tarsal claw and more pronounced pronotal lateral carina “sutures inferieures” in comparison to subgenus Phorocardius. However, Douglas (2017) found *Phorocardius* polyphyletic and elevated *Diocarphus* to genus status with diagnoses including procoxal cavity closure and female internal genitalic structures.

With only three species previously known (Cate et al. 2007), the *Phorocardius* fauna of China remained little known. In this paper, we study the taxonomy of Chinese *Phorocardius* and describe six new species. Additionally, we designate lectotypes for *Cardiophoruscomptus* Candèze, 1860, *Cardiophoruscontemptus* Candèze, 1860, *Phorocardiusmagnus* Fleutiaux, 1931, and *Cardiophorusmanuleatus* Candèze, 1888; and the holotype of *Cardiophorusyanagiharae* Miwa, 1927 is identified. These were designated (or identified) to fix species concepts and to ensure their universal and consistent interpretation.

## Materials and methods

**Figure 1.**
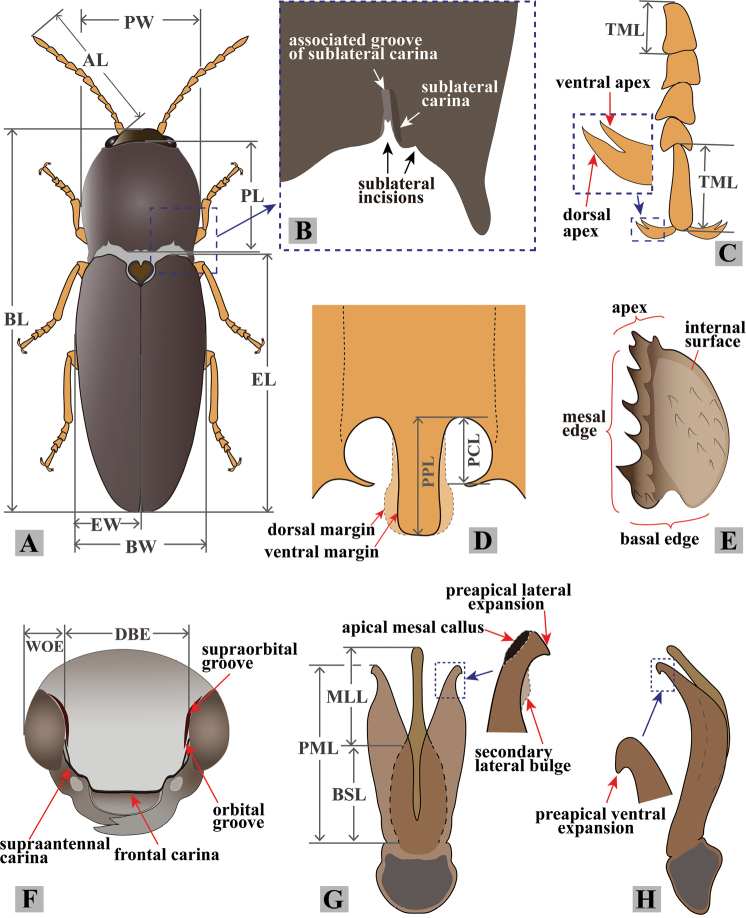
Explanation of measurement and terminology. **A** male habitus (AL: antenna length; BL: body length; BW: body width; EL: elytron length; EW: elytron width; PL: pronotum length; PW: pronotum width) **B** postero-lateral part of pronotum **C** tarsomeres I to V (TML: tarsomere length), with inset showing bifid claw **D** posterior part of prosternum, showing prosternal process, arrows showing its dorsal and ventral margins (PCL: procoxal cavity length; PPL: prosternal process length) **E** proximal sclerite of bursa copulatrix, showing internal surface: with a concavity on basal edge, large spines on mesal edge and minute ones on disc **F** head, frontal view (DBE: distance between eyes; WOE: width of eye) **G** aedeagus, ventral view (BSL: basal strut length; MLL: median lobe length; PML: paramere length), inset showing apical part of paramere **H** aedeagus, lateral view, inset showing apical part of paramere.

Observations of the habitus and diagnostic characters were made using a NIKON SMZ645 stereo microscope and a NIKON E100 optical microscope. Digital images were taken using a CANNON D800 camera attached to a CANNON MP-E 65 mm Lens or NIKON E100 microscope. Before dissection, dry specimens were submerged in hot water for 10 minutes. Male genitalia were subsequently dissected and glued to card papers pinned under the specimens. Female genitalia were submerged in hot 10% NaOH solution for approximately 1 minute, surrounding tissues were cleared, mounted in glycerin on slides for photography, and then glued to card papers pinned under the specimens. Specimen measurements were made as shown in Fig. 1.

Specimens were identified using species identification keys (e.g., Candèze 1860; Fleutiaux 1947), and additional species descriptions. We were able to examine type material for most species only occurring outside the study area. The remainder were excluded from the Chinese fauna based on literature alone. Details are presented in Results: Checklist and synonymy of world *Phorocardius* species.

Morphological terminology and the generic concept of *Phorocardius* follow Douglas (2017).

Definition of Oriental and Palearctic Regions mainly follows Wallace (1876) and Morrone (2015), south Tibet and the Yangtze River valley in central China is treated as the boundary (or transition zone) between the two regions. In the ‘Type Material’ and ‘Material’ sections of each species, specimen data are recorded verbatim from labels. Specimens were examined from the following insect collections:

**IZCAS**Institute of Zoology, Chinese Academy of Sciences, Beijing, China.

**LQCC** Lu Qiu Personal Collection, Chongqing, China.

**MNHN** Museum national d’Histoire naturelle, Paris, France.

**NHMUK**The Natural History Museum (formerly British Museum), London, United Kingdom.

**NHMW**Naturhistorisches Museum Wien, Wien (Vienna), Austria.

**RBINS**Royal Belgian Institute of Natural Sciences (Institut Royal des Sciences Naturelles de Belgique), Brussels, Belgium.

**SZPT**School of Applied Chemistry and Biological Technology, Shenzhen Polytechnic, Shenzhen, Guangdong, China.

**TARI **Taiwan Agricultural Research Institute, Taichung, Taiwan, China.

## Results

### Phorocardius

Taxon classificationAnimaliaColeopteraElateridae

Fleutiaux, 1931

E720FE17-A119-5D30-AA0A-12EEBB89E1F2

Phorocardius Fleutiaux, 1931: 308. Type species: Cardiophorusflorentini Fleutiaux, 1895.

#### Distribution of known species.

Oriental and southeast Palearctic Regions: China (Inner Mongolia, Shaanxi, Henan, Sichuan, Hubei, Guizhou, Yunnan, Guangxi, Taiwan, Hainan), Myanmar, India, Laos, Nepal, Thailand, Vietnam, Sri Lanka, Cambodia, Maldives (doubtful).

#### Description of *Phorocardius* based on species from China.

Body length 5–13.9 mm. Width 1.6–4.5 mm. Integument black, brown, yellow and/or red, some with spots or stripes on pronotum or elytra. Body with yellow to yellow-grey pubescence (brown setae present on disc of pronotum in *P.florentini* and *P.zhiweii* Ruan Douglas & Qiu, sp. nov.).

***Head*.** Hypognathous. Frons and vertex convex, flat or weakly concave; frontal carina (joined supraantennal carinae, raised above labrum) convex or straight in dorsal view; carina smooth and glabrous; supraantennal carinae forked near junctures with compound eyes (Fig. 23, indicated by arrows), weakly separated in rare cases (e.g., in *P.manuleatus*, see Fig. 23E); frons with supraorbital and orbital grooves present and shallow (Fig. 1F). Antennae not reaching or slightly exceeding posterior angles of pronotum; antennal sensory elements beginning on antennomere 3. Mandible with apex bidentate to tridentate; apical palpomere of maxillary hatchet-shaped or polygonal, longer than wide. Labrum evenly convex. Area between each antennal fossa and adjacent compound eye unsculptured, or with groove or pit(s).

***Prothorax*.** Pronotum in dorsal view with sides straight, convex, or sinuate near posterior fourth. Pronotum with punctures circular or oval on dorsal surface, punctures larger and deeper on disc and anterad, sparser and smaller posterad; sublateral incisions and carinae present (with carinae obsolete, see Fig. 1B); posterior edge of pronotum sinuate, with three apices mesally (tridentate antescutellar lobe, i.e., the median basal lobe in Douglas 2003); lateral carina on hypomeron not present (or not distinguishable from hind angle carina); posterior angles not truncate dorsally; hind angle carina not extending anterad beyond posterior third; anterior angles obtuse and not projecting anterad, posterior angles straight-sided, slightly convex or strongly bulged laterally (e.g., Fig. 17C, D), parallel to weakly divergent; hypomeral hind edges rectangularly emarginate (Fig. 12B, indicated by arrow) immediately meso-ventrad of posterior angles. Procoxal cavities open or closed. Prosternum with sides concave in ventral view; anterior prosternal lobe long, covering labium when head is retracted; prosternal process curved dorsad or not, ventral surface convex to flat, carinate laterally or not; prosternal process approximately twice as long as procoxal cavity length.

***Pterothorax*.** Scutellar shield heart-symbol shaped, with anterior edge emarginate (Fig. 13D), anterolateral edges sinuate to evenly convex, posterior apex narrowly rounded (Fig. 13D) to pointed (Figs 11A, 15A), strongly elongate and produced posteriorly in some (e.g., in *P.florentini*). In lateral view, mesosternum with anterior edges concave (Fig. 24); anterior facing projections on posterior edge of mesosternum (i.e., on anteroventral angle of mesosternal fossa according to Douglas (2003)) strongly developed, sharp and produced anteriorly to absent (Fig. 25, indicated by solid line and lower red arrow). In ventral view, mesosternal fossa approximately diamond-symbol shaped (Fig. 25), with lateral edges sinuate anterad of mesocoxae (Fig. 25); antero-mesal angle of mesepisternum broadly rounded to acute, facing antero-mesally (Fig. 25, indicated by dashed line and green arrow). Elytra with humeral angle angulate or tuberculate in dorsal view; interstriae prominently convex near base in most, gradually becoming less convex on apical half; upper edge of epipleura with minute serrations. Hind wings large, and apparently capable of flight; notched in anal area or not (Douglas 2017).

***Legs*.** Tarsi simple; tarsomere V longest; tarsal claws each with two apices, apices separated in apical half of claw, with ventral surface of claw sinuate basad of ventral apex, ventral apex much smaller than to almost as large as dorsal apex. Metacoxal plate large, covering 1/2–2/3 of metatrochanter with legs withdrawn.

***Abdomen*.** Lateral edges of visible abdominal ventrites I–V (i.e., urosternites III–VII) with or without minute serrations.

***Male genitalia*.** Urosternite VIII straight to anteriorly pointed, with two lateral posterior lobes, without median posterior lobe; abdominal segment IX with tergite and sternites articulated at sides. Aedeagus: paramere, with or without preapical lateral expansion (Fig. 1G), with preapical ventral (or apical mesal) expansion in some (Fig. 1H), apical mesal callus (in most oval, disc-like, with sclerotized sharp edge; see Figs 1G, 4G) present or absent, lateral side with two setae near apex; aedeagus with basal strut ca. 0.8–1.0 × median lobe length; in ventral or dorsal view, median lobe tapered, parallel-sided or apically expanded, apex pointed to rounded to blunt; in lateral view, apex of median lobe bent abruptly dorsad in some (e.g., in *P.magnus*); in lateral view, paramere and median lobe bent 30–45° ventrad near mid-length or apical third.

**Female.** Body of same or different color as male, some slightly longer and wider than male. Antennae of some shorter than in male. Apex of abdominal ventrite V arcuate to truncate, with deep to shallow incision on each side (Figs 9D, E, 20D), or with elongate deep invagination containing slender blade-like projection (Fig. 15C) (in male: apex of abdominal ventrite V simple, arcuate to slightly sinuate, without incision or invagination). Ovipositor with baculae present; coxites heavily sclerotized. Bursa copulatrix without sclerotized spermathecae; with paired distal and spine-bearing proximal sclerites, proximal sclerites ovoid with emarginate base to elongate and parallel-sided; distal sclerites claw-like and not fused, gradually narrowed to pointed apex; spermathecal gland duct with row of diverticulae in some, base not sclerotized inside bursa (Douglas 2017); anterior end of bursa with a single pedunculate sac.

### Checklist and synonymy of world *Phorocardius* species

**1. *Phorocardiusalterlineatus* Ruan & Douglas, sp. nov.** (details under species treatment)

**2. Phorocardiusastutus (Candèze, 1888)**

Cardiophorusastutus Candèze, 1888: 681. Type locality: “Teinzò”, Myanmar.

Phorocardiusastutus: Fleutiaux 1931: 311 (key to species).

**Distribution.** Cambodia (Fleutiaux 1931), Laos (Fleutiaux 1931), Vietnam (Fleutiaux 1931), Myanmar (Candèze 1888).

**Remarks.** Cotype specimens examined (NHMUK). Recognizable by brown-black body with dark appendages; legs with red-brown joints; parameres wedge-like with pre-apical lateral expansions.

**3. Phorocardiusbifidus (Fleutiaux, 1918)**

Cardiophorusbifidus Fleutiaux, 1918: 222. Type locality: Bangkok, Thailand.

Phorocardiusbifidus: Fleutiaux 1931: 311 (key to species).

**Distribution.** Thailand (Fleutiaux 1918), Laos (Fleutiaux 1931).

**Remarks.** Type specimen examined (MNHN). Body brown-black with red-brown appendages; and yellow pubescence. Aedeagus with median lobe parallel-sided, with rounded apex, parameres narrowed to a point without preapical expansions.

**4. *Phorocardiuscomptus* (Candèze, 1860)** (details under species treatment)

**5. Phorocardiuscontemptus (Candèze, 1860) comb. nov. Fig 29**

Cardiophoruscontemptus Candèze, 1860: 202. Type locality: “Hindoustan meridional; Pondichery et Mysore” (India: Pudicherry; Karnataka, Mysore). Lectotype designated here.

**Distribution.** India (Candèze 1860), Myanmar (‘Thagatà’; Candèze 1888), Bangladesh (‘Bengale’; Candèze 1891), Vietnam? (‘Cochinchine’; Fleutiaux 1918; doubtful record).

**Remarks.** Only a single female syntype was discovered in NHMUK: integument entirely black with dark legs, pubescence yellow. Bursa copulatrix with proximal sclerites (from internal view) ovoid with slight basal concavity; spines present on convex mesal edge, both sides of apex and flattened internal surface. The male syntype described in the original paper was not seen in NHMUK. Since Candèze’s collection before 1869 had been transferred to the NHMUK (Bousquet 2016), this female specimen is studied and designated as the lectotype to fix species concepts.

The examination of the lectotype shows that it resembles *P.comptus*Candèze (1860) in the female genitalia and external characters except for the all-black elytral color. Candèze (1860) also commented that “This may be an entirely black variety of comptus”. It is possible that *C.contemptus* is conspecific with *P.comptus*. Because we have not studied any male specimen, *C.contemptus* is treated here as valid.

**Type material.** (NHMUK) (Photographs of syntype provided by Ms Karine Savard, Agriculture and Agri-food Canada). ***Lectotype***. ♀, labels: 1) Syntype [blue ringed disk]; 2) [female symbol]; 3) [red square]; 4) [red square]; 5) 215; 6) C75; 7) Ind. Or Moussour ca; 8) *Cardioph.*ContemptusCdzésec.Cdze; 9) Janson Coll. Ex. Deyrolle. 1903.130; 10) NHMUK04016800; 11) Lectotype, *Cardiophoruscontemptus* Candèze, 1860, Des. Ruan & Douglas, 2020.

**6. Phorocardiuserythronotus (Candèze, 1860)**

Cardiophoruserythronotus Candèze, 1860: 212. Type locality: India: Patna, Dinapur.

Phorocardiuserythronotus: Ôhira 1978: 96 (distribution, photograph of habitus and diagnostic characters).

**Distribution.** India (Candèze 1860), Nepal (Ôhira 1978).

**Remarks.** Type material examined (NHMUK). Body brown-black; prothorax, head and antennae red-yellow to red-brown; pronotum in lateral view with lateral carina diverging from hind angle carina. This species is probably not *Phorocardius* because of the presence of pronotal lateral carina. It was not transferred outside *Phorocardius* because we were unable to make a well-supported generic placement without data from female morphology.

**7. *Phorocardiusflavistriolatus* Ruan & Douglas, sp. nov.** (details under species treatment)

**8. *Phorocardiusflorentini* (Fleutiaux, 1895)** (details under species treatment)

**9. *Phorocardiusmagnus* Fleutiaux, 1931** (details under species treatment)

**10. Phorocardiusmaldivianus Platia, 2015**

Phorocardiusmaldivianus Platia, 2015: 184. Type locality: “Maldives, Meemu Atoll, Kureli Island”.

**Distribution.** Maldives (Platia 2015).

**Remarks.** This species is unlikely to truly belong to *Phorocardius* because the pronotal lateral carina diverges from the hind angle carina. The distinctive arcuate parameres with bulbous apices distinguish this species from those of any *Phorocardius* examined by the authors. However, *P.maldivianus* was not transferred outside *Phorocardius* because we were unable to make a well-supported generic placement without data from female morphology or DNA.

**11. *Phorocardiusmanuleatus* (Candèze, 1888)** (details under species treatment)

**12. Phorocardiusmelanopterus (Candèze, 1878)**

Cardiophorusmelanopterus Candèze, 1878: 38. Type locality: Cambodia.

Phorocardiusmelanopterus: Fleutiaux 1931: 311 (key to species).

**Distribution.** Cambodia (Candèze 1878).

**Remarks.** Type material examined (RBINS, photograph examined): the head is brown-black, the remainder of the body is brown throughout including the legs and basal four antennomeres (remaining antennomeres are lost), and pronotum with lateral carina diverging from hind angle carina. This species is probably not *Phorocardius* because of having a pronotal lateral carina. It was not transferred outside *Phorocardius* because we were unable to make a well-supported generic placement without data from female morphology.

**13. *Phorocardiusminutus* Ruan & Douglas, sp. nov.** (details under species treatment)

**14. Phorocardiusmoorii (Candèze, 1860)**

Cardiophorusmoorii Candèze, 1860: 206. Type locality: “Madras” (India).

Phorocardiusmoorii: Ôhira 1978: 96 (distribution, photograph of habitus and diagnostic characters)

Phorocardiusmoorii: Cate et al. 2007: 206 (distribution).

**Distribution.** India (Candèze 1860). Nepal (Ôhira 1978).

**Remarks.** Body black, with four round yellow spots on its elytra. This species is probably a junior synonym of *Elatertetraspilotus* Hope (syntypes of *E.tetraspilotus* and *C.moorii* housed in NHMUK examined). However, dissection of type specimens is required to confirm possible synonymy.

**15. *Phorocardiusrufiposterus* Ruan & Douglas, sp. nov.** (details under species treatment)

**16. Phorocardiussystenus (Candèze, 1860)**

Cardiophorussystenus Candèze, 1860: 210. Type locality: “Hindoustan” (India).

Platynychussystenus: Miwa 1934: 212 (distribution).

Phorocardiussystenus: Ôhira, 1978: 96 (distribution, photograph of habitus, and diagnostic characters).

**Distribution.** India (Candèze 1860), Nepal (Ôhira 1978).

**Remarks.** Type examined (NHMUK). *Cardiophorussystenus* Candèze, 1860 was transferred to *Phorocardius* by Ôhira (1978). However, Miwa (1934) had already treated the same species as a member of the genus *Platynychus*. This species was treated separately as both *Platynychus* and *Phorocardius* by Cate et al. (2007). This species is closer to *Platynychus* rather than *Phorocardius* because, in lateral view, the pronotum has the lateral carina diverging from hind angle carina. However, here we treat this species as a member of *Phorocardius* until more evidence is gathered for transferring it to another genus. Furthermore, the examination of the type specimens of *Cardiophorusbucculatus* Candèze, 1860 and *Cardiophorussystenus* Candèze, 1860 indicates they are probably conspecific.

**17. *Phorocardiusunguicularis* Fleutiaux, 1918** (details under species treatment)

**18. Phorocardiusvicinus (Kollar, 1848)**

Cardiophorusvicinus Kollar, 1848: 507. Type locality: Kashmir.

Phorocardiusvicinus: Cate et al. 2007: 206 (distribution).

**Distribution.** India: Kashmir (Cate 2007).

**Remarks.** Type specimen examined by photographs (NHMW): 9 mm long, black with dark legs, pubescence yellow. Bursa copulatrix with proximal sclerites ovoid without basal concavity; spines present on convex mesal edge, both sides of apex and flattened internal surface.

**19. *Phorocardiusyanagiharae* (Miwa, 1927)** (details under species treatment)

**20. *Phorocardiusyunnanensis* Ruan & Douglas, sp. nov.** (details under species treatment)

**21. *Phorocardiuszhiweii* Ruan, Douglas & Qiu, sp. nov.** (details under species treatment)

#### Species transferred from *Phorocardius* to *Displatynychus*

The following *Phorocardius* species are shown, in their original publications (or in the examined type specimens) to have the diagnostic characters of *Displatynychus* and not *Phorocardius*. While these species have *Phorocardius*-like claws, they are shown to have the following diagnostic characters of *Displatynychus*: pronotal lateral carina distinct from hind-angle carina and hidden in dorsal view by overhanging edge of upper part of pronotum; and female bursa copulatrix with base of spermathecal gland duct sclerotized into complex tube-like structure.

Recently described *Phorocardius* species from south Asia (*P.maldivianus* Platia, 2015; *P.pakistanicus* Platia & Ahmed, 2016; *P.tibialis* Platia & Ahmed, 2016) could be distinguished from all Chinese *Phorocardius* species using the species descriptions. We recommend the transfer of *P.pakistanicus* and *P.tibialis* to *Displatynychus* Ôhira, 1987. However, we do not recommend the transfer of *P.maldivianus* outside *Phorocardius* because we were unable to make a well-supported generic placement without data from female morphology or DNA.

**1. Displatynychusbombycinus (Candèze, 1895) comb. nov.**

Cardiophorusbombycinus Candèze, 1895: 46. Type locality: “Darjeeling” (India).

Phorocardiusbombycinus: Cate et al. 2007: 206 (distribution).

**Distribution.** India: west Bengal (Candèze 1895).

**Remarks.** This species matches *Displatynychus* and not *Phorocardius* in two key diagnostic characters listed above. Type specimen examined (RBINS): “Coll. R.I.Sc.N.B./ Inde Kurseong”; “Collection/ E. Candeze”; “n.sp. 11. 1982/ Bombycinus/ Cand./ Kurseong”; type has pronotum with lateral carina diverging from hind angle carina and nearly reaching the anterior edge of the pronotum. Female specimen from India (NHMUK): Darjeeling (NHMUK014016975), with base of spermathecal gland duct sclerotized into complex tube-like structure and distal sclerites absent.

**2. Displatynychuspakistanicus (Platia & Ahmed, 2016) comb. nov.**

Phorocardiuspakistanicus Platia & Ahmed, 2016: 16. Type locality: “Pakistan, Thar”.

**Distribution.** Pakistan (Platia and Ahmed 2016).

**3. Displatynychussobrinus (Laporte, 1840) comb. nov.**

Caloderussobrinus Laporte, 1840: 250. Type locality: “Hindoustan” (south Asia).

Cardiophorussobrinus: Candèze 1860: 210. (key to species, redescription).

Dicronychussobrinus: Ôhira 1973: 38 (distribution).

Phorocardiussobrinus: Ôhira 1978: 95 (distribution, photograph of habitus, and diagnostic characters).

Phorocardiussobrinus: Cate et al. 2007: 206 (distribution).

**Distribution.** India (Ôhira 1973), Sri Lanka (Laporte 1840; Candèze 1860; Ôhira 1973), Cambodia (Fleutiaux 1918), Nepal (Ôhira 1978).

**Remarks.** This species matches *Displatynychus* and not *Phorocardius* in two key diagnostic characters mentioned above. Type material was not examined (probably in MNHN), but non-type specimens at NHMUK (Janson Coll.) had pronotum with lateral carina diverging from hind angle carina and nearly reaching the anterior edge of the pronotum. Female specimen at NHMUK (NHMUK014017117) with base of spermathecal gland duct sclerotized into complex tube-like structure and distal sclerites absent.

**4. Displatynychustibialis (Platia & Ahmed, 2016) comb. nov.**

Phorocardiustibialis Platia & Ahmed, 2016: 17. Type locality: “Pakistan, Chakri, Islamabad”.

**Distribution.** Pakistan (Platia and Ahmed 2016).

### Taxonomy of Chinese *Phorocardius* species

#### Key to Chinese *Phorocardius* species

**Table:** 

1	Procoxal cavities closed (Fig. 15E, narrowly open in rare cases); color of dorsum and venter anteriorly black, fading to red-brown or yellow-brown posterad; apex of last abdominal ventrite (ventrite V) of female deeply emarginate, with slender blade-like projection at middle (Fig. 15C); from dorsal and ventral views, paramere of aedeagus with preapical lateral expansion triangular, not acute (Fig. 14G)	***P.rufiposterus* sp. nov.**
–	Procoxal cavities open (e.g., Fig. 25A); body color not fading from black to paler posterad; apex of last abdominal ventrite (ventrite V) of female without longitudinal slender blade-like structure; from dorsal and ventral views, paramere of aedeagus with preapical lateral expansion absent, rounded or acute, not triangular	**2**
2	Body length less than 7.0 mm; scutellar shield with antero-lateral edge evenly convex and posterior apex narrowly rounded; tarsal claw with ventral apex much smaller than dorsal one (Fig. 12A, indicated by arrow); apex of paramere pointed and turned laterad in ventral view (Fig. 12E, I, indicated by arrows)	***P.minutus* sp. nov.**
–	Body length greater than 7.0 mm; scutellar shield with antero-lateral edge sinuate and posterior apex pointed; tarsal claw with ventral apex as large as dorsal one (Figs 4A, 10B, indicated by blue arrow); paramere apex not pointed and bent laterad	**3**
3	Elytra with metallic luster; pronotum and hypomeron entirely red except posterior edge red-brown or black	**4**
–	Elytra without metallic luster; pronotum and hypomeron black, brown or with longitudinal midline black and remainder red	**5**
4	Elytra black, with metallic blue to purple luster; paramere with preapical lateral expansion in ventral view, (Fig. 7E), with sides rounded, facing laterally, without apical mesal callus; prosternal process with outline of ventral apex rounded-rectangular in ventral view (Fig. 25C)	***P.florentini* (Fleutiaux, 1895)**
–	Elytra metallic green; paramere of aedeagus without preapical lateral expansion, with apical mesal callus (Fig. 21D); prosternal process with outline of ventral apex evenly rounded in ventral view (Fig. 25I)	***P.zhiweii* sp. nov.**
5	Dorsum bicolored, with yellow to red maculation; if dorsum unicolor, apex of median lobe of aedeagus dilated in ventral view (Figs 10D, F, 11F)	**6**
–	Dorsum unicolored, without maculation; in ventral view, apex of median lobe of aedeagus not dilated	**8**
6	Body dark brown, elytra with longitudinal yellow stripes; ventral surface of prosternal process not strongly narrowed posterad in ventral view, with apex truncate to slightly convex (Fig. 25A, B); paramere without preapical lateral expansion in ventral view	**7**
–	Body entirely black, yellow or mixed with both yellow and black (Fig. 11A); ventral surface of prosternal process strongly narrowed posterad in ventral view, with apex acute (Fig. 25E); paramere with small and round preapical lateral expansion in ventral view (Fig. 11F)	***P.manuleatus* (Candèze, 1888)**
7	Each elytron with three separate slender longitudinal yellow stripes on interstriae III, V and VII (Fig. 2A); aedeagus gradually narrowed from base to apex in lateral view (Fig. 2H); apex of median lobe narrowly rounded to angulate in ventral view (as in Fig. 2G, I)	***P.alterlineatus* sp. nov.**
–	Each elytron with a single longitudinal yellow stripe covering basal half of interstria IV and interstriae V to VII. (Fig. 4A, C); in lateral view, aedeagus of equal thickness from base to apical fifth, only slightly narrowed at apical fifth (as in Fig. 4N); apex of median lobe truncate to broadly rounded in ventral view	***P.flavistriolatus* sp. nov.**
8	Head with frontal carina convex in frontal view (Fig. 16C). Pronotum with interspaces between punctures 0.3–1 × average puncture diameter (Fig. 16D). Elytral length to pronotal length ratio 2.7–3.1. Paramere of aedeagus with apex needle-like and simple, without preapical lateral expansion (Fig. 16F)	***P.unguicularis* (Fleutiaux, 1918)**
–	Head with frontal carina straight in frontal view. Pronotum with interspaces between punctures 1–3 × average puncture diameter. Elytral length to pronotal length ratio 2.5–2.7. Paramere of aedeagus not needle-like at apex, with preapical lateral expansion	**9**
9	Posterior angle of pronotum with lateral edge convex, strongly bulged laterally in dorsal view (Fig. 17C, D). Median lobe of aedeagus with apex not dilated or bent dorsad in lateral view (Fig. 17G)	***P.yanagiharae* (Miwa, 1927)**
–	Posterior angle of pronotum with lateral edge straight to slightly convex, not strongly bulged in dorsal view. Median lobe of aedeagus with apex dilated (Fig. 19F) or bent dorsad (Fig. 8G) in lateral view	**10**
10	Dorsum matt, brown to red-brown. Aedeagus with apex of median lobe bent abruptly dorsad in lateral view (Fig. 8G); in ventral and dorsal views, paramere with a secondary lateral bulge present before preapical lateral expansion (Fig. 8D, E, F, indicated by arrows)	***P.magnus* Fleutiaux, 1931**
–	Dorsum entirely black and shiny. Aedeagus with apex of median lobe not bent dorsad in lateral view (Fig. 19F); in ventral and dorsal views, paramere without a secondary lateral bulge (Fig. 19D, E)	***P.yunnanensis* sp. nov.**

##### 1. Phorocardius alterlineatus

Taxon classificationAnimaliaColeopteraElateridae

Ruan & Douglas sp. nov.

42C922B4-6308-5E3A-A676-696B92CD60B4

http://zoobank.org/E5EEBBF2-D73F-498B-88C4-99452A547603

**Figure 2.**
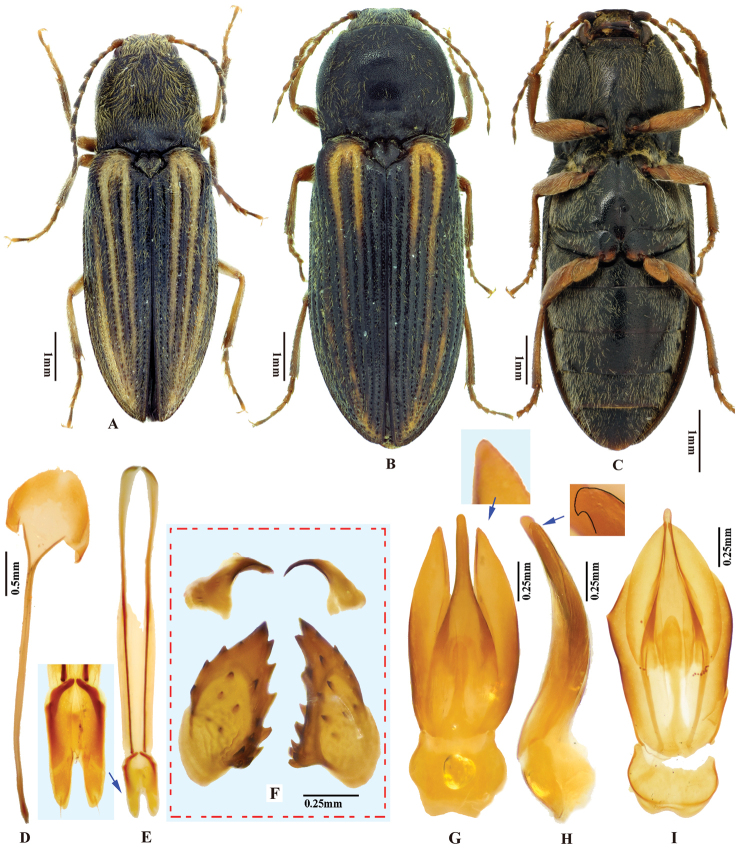
*Phorocardiusalterlineatus* Ruan & Douglas, sp. nov. **A** male habitus, paratype (Shaanxi, Zha-shui County, Niu-bei-liang), dorsal view **B** female habitus, paratype (Shaanxi, Shan-luo, Zha-shui County, Ying-pan township), dorsal view **C** female habitus, paratype (same individual as in inset B), ventral view **D** female abdominal sternite VIII **E** ovipositor, dorsal view (paratype), arrow indicating coxites **F** distal (upper side) and proximal sclerites of bursa copulatrix (the two proximal sclerites are viewed from different angles, resulting in different shapes shown above) **G** aedeagus, dorsal view (paratype, dry), arrow indicating apex of paramere **H** aedeagus, lateral view (paratype, dry), arrow indicating apex of paramere. **I** aedeagus (mounted in glycerin), dorsal view (paratype).

**Figure 3.**
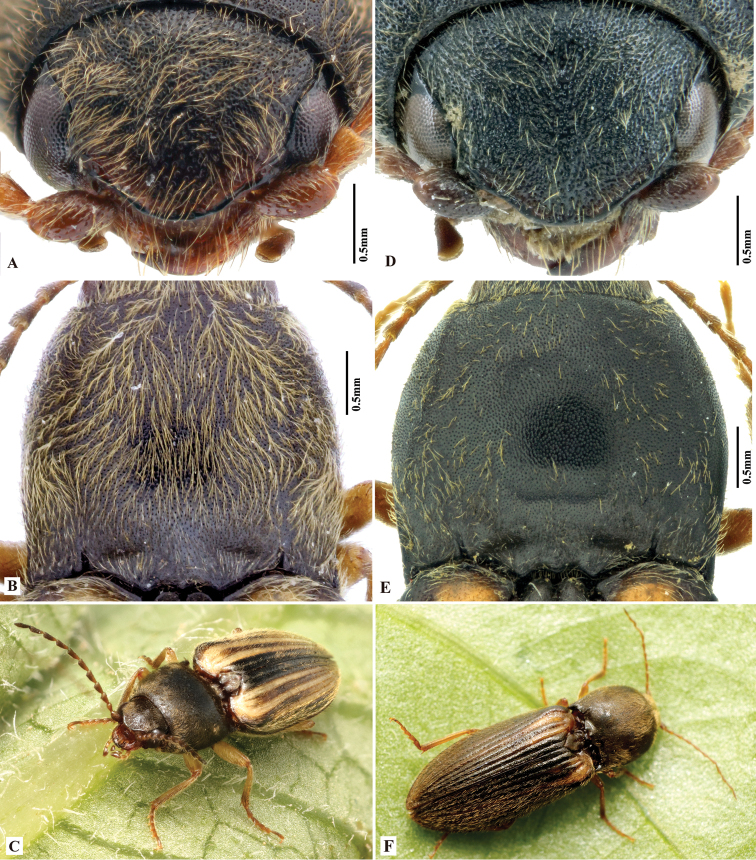
*Phorocardiusalterlineatus* Ruan & Douglas, sp. nov. **A** male head, frontal view (paratype) **B** male pronotum, dorsal view (paratype) **C** male on foliage (indoors; holotype) **D** female head, frontal view (paratype) **E** female pronotum, dorsal view (paratype) **F** female on foliage (indoors; paratype; collected from Shaanxi, Yan-an).

Figs 2 , 3 , 23A , 24A , 25A , 26A

###### Type locality.

Shaanxi Prov., Yan-an (36.622°N, 109.457°E, alt. 993 m).

###### Etymology.

The name of this species refers to the alternating longitudinal maculation on elytra.

###### Distribution.

China (Shaanxi, Hubei, Sichuan, Guangxi).

###### Differential diagnosis.

Body length greater than 7.0 mm; integument dark brown (non-metallic), each elytron with three separate yellow stripes along interstriae III, V, and VII. Prothorax: procoxal cavities open. Prosternal process not strongly narrowed posterad to ventral apex in ventral view, with apex truncate to slightly rounded. Pterothorax: scutellar shield with posterior apex pointed. Tarsal claw with ventral apex not smaller than dorsal apex. Male genitalia: paramere without preapical lateral expansion or apical mesal callus. Female: apex of last abdominal ventrite (ventrite V) simple, not emarginate at apex.

This species is unique in *Phorocardius* in its alternating dark and yellow stripes on the elytra, the aedeagus is also unique due to its simple shape: without any preapical lateral expansion (but with ventral hook-like expansion).

*Phorocardiusalterlineatus* Ruan & Douglas, sp. nov. resembles *P.flavistriolatus* Ruan & Douglas, sp. nov. and *P.comptus* (Candèze, 1860) in having longitudinal yellow maculation on the elytron, but it could be distinguished from the latter two species by the following characters. In *P.alterlineatus* Ruan & Douglas, sp. nov., aedeagus gradually narrowed from base to apex in lateral view; apex of median lobe narrowly rounded in ventral view; and each elytron with three slender longitudinal stripes present separately on interstriae III, V, and VII, which partly merged near base and apex; while in *P.flavistriolatus* Ruan & Douglas, sp. nov., in lateral view, aedeagus with equal breadth from base to apical fifth, only slightly narrowed at apical fifth; apex of median lobe truncate to broadly rounded in ventral view; and each elytron with a single broad stripe covering basal half of interstria IV and interstriae V–VII.

In *Phorocardiusalterlineatus* Ruan & Douglas, sp. nov., the parameres of the aedeagus have apical lateral expansions in dorsal view and lack preapical ventral expansions in lateral view; body brown-black; and each elytron with three slender longitudinal stripes present separately on interstriae III, V, and VII, with pale stripes partly joined near base and apex; while in *P.comptus* (Candèze, 1860), the parameres lack apical lateral expansions in dorsal view and have preapical ventral expansions in lateral view; body black; and a single broad longitudinal stripe covering four interstriae (interstriae V–VIII).

###### Description.

(Based on all type specimens) Body brown-black. Each elytron with three separate longitudinal yellow stripes along interstriae III, V, and VII, partly joined near base and apex; stripes on mid-length of elytron absent or reduced in some females. Elytral stripes pale yellow in most males, orange in females. Antenna brown to dark brown, with first two antennomeres paler. Legs yellow-brown to brown, darker on tarsomere V and tibial apex. Body with yellow pubescence.

###### Measurements.

(based on all type specimens) Male body length 7.3–9.2 mm, width 2.3–3.0 mm. Female body length 8.2–10.6 mm, width 2.7–3.7 mm. Body length to width ratio 2.7–3.0. Pronotal width to length ratio 1.1–1.2. Pronotal width to body width ratio 0.83–0.90. Elytral length to pronotal length ratio 2.6–2.8; elytron length to width ratio 4.0–4.2.

***Head*.** Frons and vertex with interspaces between punctures 0.5–1.0 × average puncture diameter; punctures slightly sparser at centre of vertex. Frontal carina convex in frontal view. Distance between eyes to width of eye ratio 4.2–4.5. Antenna with last antennomere entirely reaching beyond posterior angle of pronotum in male, only reaching to posterior angle in female. Antenna length to body length ratio, in male 0.39–0.41; in female 0.35–0.37. Proportions of antennomere lengths (male): 100 (scape); 53–58; 75–81; 82–83; 81–85; 83–88; 84–86; 85–90; 85–90; 85–90; 100–130.

***Prothorax*.** Pronotum in dorsal view: sides evenly convex from anterior edge to constriction near posterior fourth, widest near mid-length; posterior angles with lateral sides almost straight, not bulged; surface with deep punctures, interspaces between punctures 0.5–1.0 × average puncture diameter. In ventral view, ventral surface of prosternal process with sides carinate and slightly and gradually narrow from anterior to posterior end, with apex almost truncate (Fig. 25A). In lateral view, prosternal process with ventral surface curved slightly dorsad; with posterior end (i.e., area between ventral and dorsal apices (sensu Douglas 2011, 2017)) slightly concave to almost straight (Fig. 24A, upper arrow). Procoxal cavities open.

***Pterothorax*** (Figs 24A, 25A). Mesepisternum in ventral view with antero-mesal corner broadly rounded, facing antero-mesally (Fig. 25A, upper (green) arrow). Projections on posterior edge of mesosternum: in ventral view present (Fig. 25A, lower (red) arrow); in lateral view present, acute, produced anteriorly (Fig. 24A, red arrow). Scutellar shield: width to length ratio 0.97–1.00; anterolateral edges slightly sinuate; posterior apex pointed. Elytra: upper edge of epipleura with minute serrations.

***Legs*.** Length ratio of metatarsomeres I–V (excluding claws): 100; 68–74; 66–70; 51–53; 125–128. Claw with ventral apex almost as large as dorsal apex.

***Abdomen*.** Lateral edges of visible abdominal ventrites I–V with minute serrations.

***Male genitalia*.** Robust in dorsal view (Fig. 2G); in lateral view slender, gradually narrowing from base to apex. Median lobe in ventral view narrowed from base to mid-length; parallel-sided and slender on apical half, apex narrowly rounded. Median lobe in lateral view gently and evenly curved ventrad from base to apex, apex rounded. Paramere in dorsal view: wide from base to mid-length, gradually narrowed beyond mid-length, angulate at apex; preapical lateral expansion and apical mesal callus absent; width 2.5–3.5 × median lobe width (measured at mid-length of paramere and median lobe respectively). Paramere in lateral view: slender, gradually narrowed and evenly curved ventrad from base to apex; apex with sharp hook-shaped preapical ventral expansion (Fig. 2H, indicated by blue arrow).

**Female.** Body color slightly blacker than male (Fig. 2B), elytral longitudinal yellow stripes partly absent on mid-length of elytron. Apex of abdominal ventrite V convex (Fig. 26A). Bursa copulatrix with proximal sclerites ovoid, apex acute, base slightly concave; with numerous spines on concave internal surface: each with 9–12 large spines on mesal edge, 6–8 smaller ones on disc.

###### Type material.

***Holotype*.** ♂ (SZPT), labels: 1) **Shaanxi**, Yan-an (延安), Yan-an University, Yao-yuan Holiday Hotel, leg. Yongying Ruan et al. 2018-VI-22–23 [in Chinese]; 2) 36.622°N, 109.457°E, 993 m; 3) Holotype *Phorocardiusalterlineatus* sp. nov. Des. Ruan & Douglas, 2019.

***Paratypes*** (100♂, 136♀). 3♀ (SZPT), labels: 1) **Shaanxi**, Yan-an (延安), Yan-an University, Yao-yuan Holiday Hotel, leg. Yongying Ruan et al. 2018-VI-22–23, light trap [in Chinese]; 2) 36.622°N, 109.457°E, 993 m; 3) Paratype *Phorocardiusalterlineatus* sp. nov. Des. Ruan & Douglas, 2019. • 2♀ (IZCAS), labels: 1) **Shaanxi**, Liu-ba County (留坝县), Huo-shao-dian, Hong-ya-gou, 2012.VI.23, 33.51°N 106.93°E, 986 m, leg. Yi Hua [in Chinese]; 2) Paratype *Phorocardiusalterlineatus* sp. nov. Des. Ruan & Douglas, 2019. • 1♀ (IZCAS), labels: 1) **Shaanxi**, Tai-bai County (太白县), Huang-bai-yuan, He-tao-ping, 2012.VI.19, 33.822°N 107.556°E, leg. Li Sha, light trap [in Chinese]; 2) Paratype *Phorocardiusalterlineatus* sp. nov. Des. Ruan & Douglas, 2019. • 1♂ (IZCAS), labels: 1) **Shaanxi**, Liu-ba County (留坝县), 2012.VI.21, 33.616°N 106.907°E, 980 m, leg. Li Sha; 2) Paratype *Phorocardiusalterlineatus* sp. nov. Des. Ruan & Douglas, 2019. • 1♂7♀ (in IZCAS, 1♀ to be transferred to LQCC), labels: 1) **Shaanxi**, Qin-ling Botanical Garden (秦岭植物园), grand canyon, 2012.VII.5, 33.9282°N 108.3525°E, leg. Hua Yi, 925 m [in Chinese]; 2) Paratype *Phorocardiusalterlineatus* sp. nov. Des. Ruan & Douglas, 2019. • 1♀ (IZCAS), labels: 1) **Shaanxi**, Qin-ling Botanical Garden (秦岭植物园), Bai-yang-ji-li-gou, 2012.VII.6, 33.9827°N 108.3282°E, leg. Hua Yi, 698–738 m [in Chinese]; 2) Paratype *Phorocardiusalterlineatus* sp. nov. Des. Ruan & Douglas, 2019. • 1♂ (IZCAS), labels: 1) **Shaanxi**, Zha-shui County, Niu-bei-liang (牛背梁), 2013.VII.1–2, leg. Junzhi Cui & Yuanyuan Lu, 33.85742°N, 108.99886°E [in Chinese]; 2) Paratype *Phorocardiusalterlineatus* sp. nov. Des. Ruan & Douglas, 2019. • 1♀ (IZCAS), labels: 1) **Shaanxi**, Shan-luo, Zha-shui County, Ying-pan township (营盘镇), Light trap 2014.VII.29, leg. Yuanyuan Lu, Chinese Academy of Sciences [in Chinese]; 2) 955 m, pure ethanol, 33.776572°N 109.043397°E, Chinese Academy of Sciences [in Chinese]; 3) Paratype *Phorocardiusalterlineatus* sp. nov. Des. Ruan & Douglas, 2019. • 53♂67♀ (SZPT), labels: 1) **Shaanxi**, Zi-wu-ling reserve (子午岭保护区), Hua-shu-gou, colleting method FIT, 2019.VI–VII [in Chinese]; 2) Paratype *Phorocardiusalterlineatus* sp. nov. Des. Ruan & Douglas, 2020. • 28♂38♀ (SZPT), labels: 1) **Shaanxi**, Zi-wu-ling reserve (子午岭保护区), Shi-hui-gou, colleting method FIT, 2019.VI–VII [in Chinese]; 2) Paratype *Phorocardiusalterlineatus* sp. nov. Des. Ruan & Douglas, 2020. • 9♂3♀ (SZPT), labels: 1) **Shaanxi**, Zi-wu-ling reserve (子午岭保护区), Chen-jia-he, sweeping, leg. Lei Dang, 2019.VI.26 [in Chinese]; 2) Paratype *Phorocardiusalterlineatus* sp. nov. Des. Ruan & Douglas, 2020. • 5♂9♀ (SZPT), labels: 1) **Shaanxi**, Zi-wu-ling reserve (子午岭保护区), Chen-jia-he, colleting method MT-5, 2019.VII.5–19 [in Chinese]; 2) Paratype *Phorocardiusalterlineatus* sp. nov. Des. Ruan & Douglas, 2020. • 1♀ (SZPT, ex. LQCC), labels: 1) **Sichuan**, Jiu-zai-gou (九寨沟), 2015.6.11, leg. Hao Huang [in Chinese]; 2) Paratype *Phorocardiusalterlineatus* sp. nov. Des. Ruan & Douglas, 2019. • 1♂ (SZPT), labels: 1) **Hubei**, Wu-dang Mts., Chao-tian-gong (朝天宫), 1982.VII.5; 2) *Phorocardiuscomptus* (Candèze), det. Shihong Jiang 19; 3) 7.30*2.60 cm; 4) Paratype *Phorocardiusalterlineatus* sp. nov. Des. Ruan & Douglas, 2019. • 1♂ (SZPT), labels: 1) **Hubei**, Wu-dang Mts., Zi-xiao (紫霄), 1982.VII.10; 2) *Phorocardiuscomptus* (Candèze), det. Shihong Jiang 1993; 3) Paratype *Phorocardiusalterlineatus* sp. nov. Des. Ruan & Douglas, 2019. • 1♀ (SZPT), labels: 1) **Hubei**, Wu-dang Mts., Jin-ding (金顶), 1982.VII.9; 2) *Phorocardiuscomptus* (Candèze), det. Shihong Jiang 1993; 3) Paratype *Phorocardiusalterlineatus* sp. nov. Des. Ruan & Douglas, 2019. • 1♀ (SZPT), labels: 1) **Hubei**, Wu-dang Mts., Lao-yan (老燕), 1983.VII.2; 2) *Phorocardiuscomptus* (Candèze), det. Shihong Jiang 1993; 3) Paratype *Phorocardiusalterlineatus* sp. nov. Des. Ruan & Douglas, 2019. • 1♀ (SZPT), labels: 1) **Hubei**, Wu-dang Mts., Nan-yan (南岩), 1983.VII.2; 2) *Phorocardiuscomptus* (Candèze), det. Shihong Jiang 1991; 3) Paratype *Phorocardiusalterlineatus* sp. nov. Des. Ruan & Douglas, 2019. • 1♂ (TARI , ex. SZPT), labels: 1) **Guangxi**, Jin-xiu, Tong-shui township (桐水镇), VI-25.2006, Shenzhen Polytechnic [in Chinese]; 2) Paratype *Phorocardiusalterlineatus* sp. nov. Des. Ruan & Douglas, 2019.

###### Remarks.

Specimens examined were from low to middle elevations (0–2500 m), temperate to subtropical mountain evergreen forests in central and south China. Specimens of this species were collected at different environments using variable collecting methods, including flight interception trap (in forests), malaise trap (in forests), sweeping (on shrubs), and light trap (near a hill in suburbs of a city). Specimens came to light traps, or were caught by sweep-netting vegetation in daylight. This suggests that this species is nocturnal and diurnal. This species is known from central China (i.e., the boundary between the Palearctic and Oriental Regions), only one specimen examined is from south China (Oriental Region).

In the field, this beetle was spotted running on multiple plants. We have observed one live adult in the field with an aphid in its mouthparts. However, individuals taken back to containers in the laboratory were not observed to feed on live aphids presented there. Further evidence is required to learn the adult and larval feeding habits of this species.

Some of the paratype specimens of *P.alterlineatus* sp. nov. Ruan & Douglas were previously misidentified and used for distributional records as *P.comptus* in Jiang (1993) and Jiang and Wang (1999). The label information for these specimens is listed as follows. 1♂ (SZPT), labels: 1) Hubei, Wu-dang Mts., Chao-tian-gong (朝天宫), 1982.VII.5; 2) *Phorocardiuscomptus* (Candèze), det. Shihong Jiang 19; 3) 7.30*2.60 cm. • 1♂ (SZPT), labels: 1) Hubei, Wu-dang Mts., Zi-xiao (紫霄), 1982.VII.10; 2) *Phorocardiuscomptus* (Candèze), det. Shihong Jiang 1993. • 1♀ (SZPT), labels: 1) Hubei, Wu-dang Mts., Jin-ding (金顶), 1982.VII.9; 2) *Phorocardiuscomptus* (Candèze), det. Shihong Jiang 1993. • 1♀ (SZPT), labels: 1) Hubei, Wu-dang Mts., Lao-yan (老燕), 1983.VII.2; 2) *Phorocardiuscomptus* (Candèze), det. Shihong Jiang 1993. • 1♀ (SZPT), labels: 1) Hubei, Wu-dang Mts., Nan-yan (南岩), 1983.VII.2; 2) *Phorocardiuscomptus* (Candèze), det. Shihong Jiang 1991.

##### 2. Phorocardius flavistriolatus

Taxon classificationAnimaliaColeopteraElateridae

Ruan & Douglas sp. nov.

2D697954-5C8A-5643-99F7-12FAD8E28B63

http://zoobank.org/64BB27C0-B42E-40C7-A0AD-5CE7217C549B

**Figure 4.**
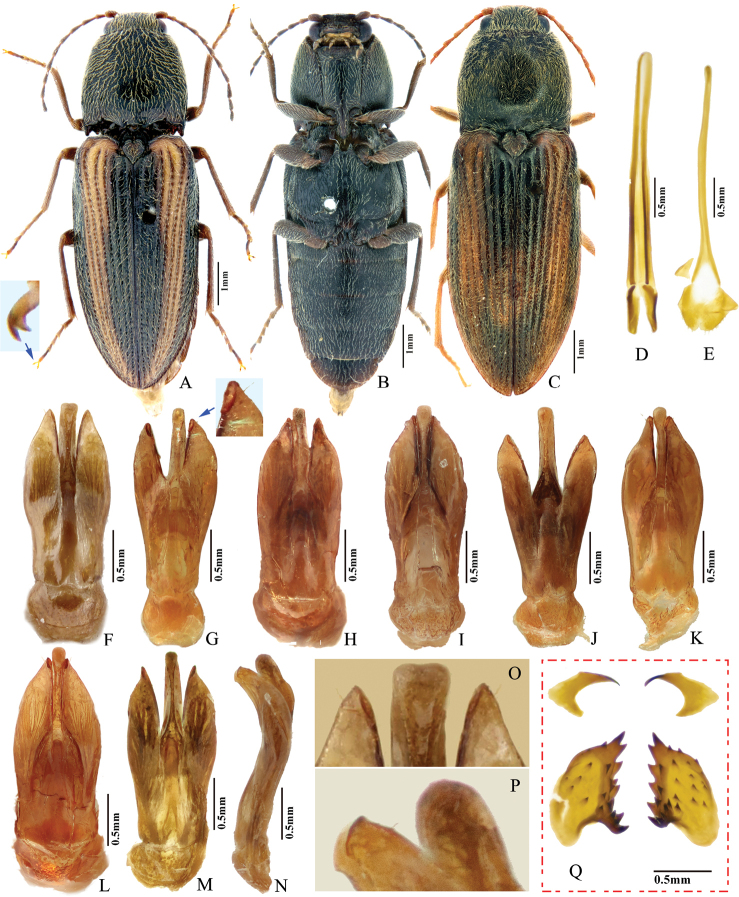
*Phorocardiusflavistriolatus* Ruan & Douglas, sp. nov. **A** habitus of holotype, male, dorsal view, arrow indicating claw **B** habitus of holotype, male, ventral view **C** female habitus, dorsal view (paratype; Sichuan, Bao-xing County, Feng-tong-zai National Nature Reserve) **D** ovipositor, dorsal view (paratype) **E** female abdominal sternite VIII, dorsal view (paratype) **F** aedeagus of holotype, ventral view **G–N** ventral or lateral view of aedeagi of paratype specimens from different localities, indicating variations in shape [collecting information as follows **G, H** Sichuan Prov., Shi-mian, Li-zi-ping National Nature Reserve, Zi-ma reserve station, VII-27-2016 **I** Sichuan, Lu-ding County, Xin-xing township, 2000 m, 2009.VII.2–7 **J, K** Sichuan Prov., Lu-ding County, Mo-xi township, 1500 m–1600 m,1983.VI.17–19 **L** Shaanxi Prov., Fo-ping County, alt. 843 m, 2007.VIII.16 **M, N** Shaanxi Prov., Ning-shan County, Shi-ba-zhang-pu-bu Scenic Spot, 15.VIII.2013], arrow indicating apex of paramere **O** apical part of aedeagus of holotype, ventral view **P** apex of aedeagus of paratype, lateral view **Q** distal (upper side) and proximal sclerites of bursa copulatrix (paratype).

**Figure 5.**
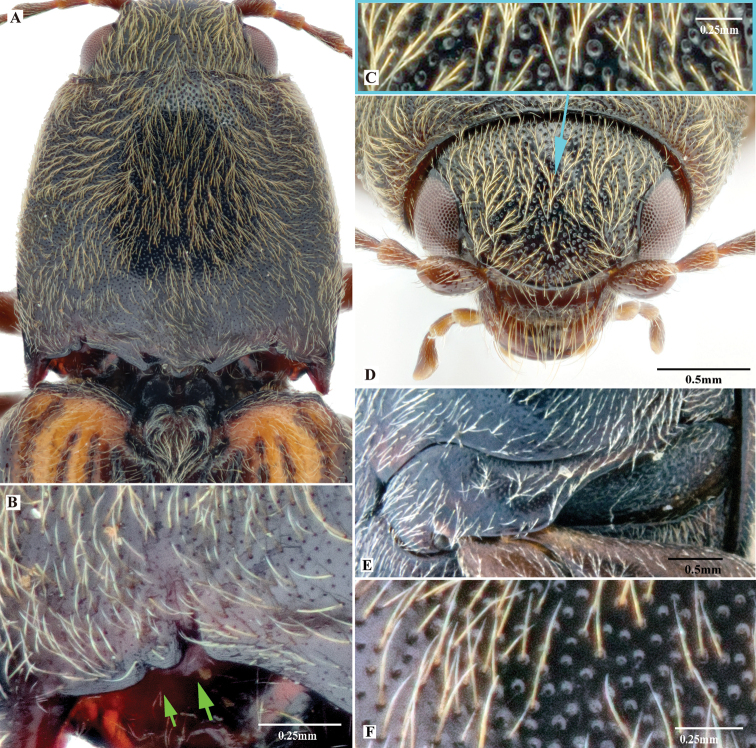
*Phorocardiusflavistriolatus* Ruan & Douglas, sp. nov. **A** pronotum, dorsal view (holotype) **B** posterior edge of pronotum, left side, arrow indicating sublateral incisions **C** punctation on centre of vertex of head **D** head, frontal view **E** left metacoxal plate, ventral view **F** punctation on disc of pronotum.

**Figure 6.**
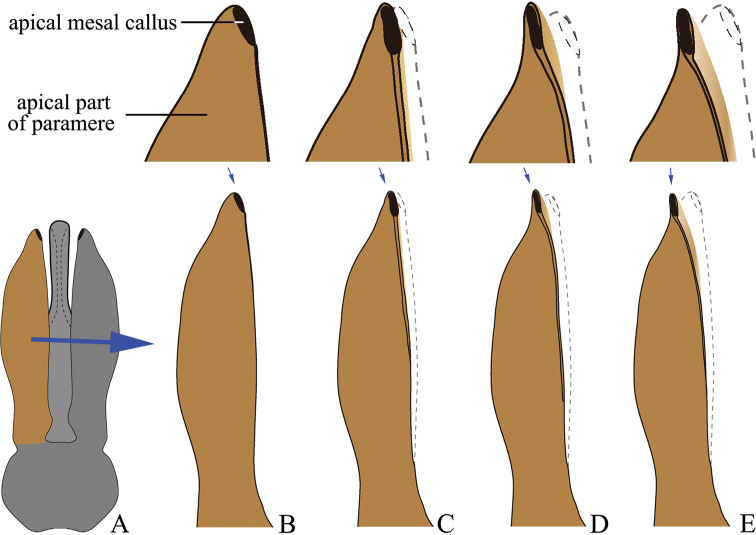
Shape variation due to rotation of paramere in *Phorocardiusflavistriolatus* Ruan & Douglas, sp. nov. **A** aedeagus, ventral view **B–E** ventral view of paramere, showing variation; the mesal side of apical part of paramere rotated ventrad to increasing degrees, resulting in different observed shapes in ventral view, apex of paramere are shown in upper insets.

Figs 4 , 5 , 6 , 23B , 24B , 25B , 26B

###### Type locality.

China: Henan Prov., Nan-Yang City, Bao-tian-man National Nature Reserve.

###### Etymology.

The name of this species is derived from the yellow stripes on its elytra.

###### Distribution.

Central China (Henan, Shaanxi, Sichuan).

###### Differential diagnosis.

Body length 7.0–12 mm; integument dark brown (non-metallic), each elytron with a longitudinal yellow stripe covering basal half of interstria IV and interstriae V to VII. Prothorax: procoxal cavities open; prosternal process not strongly narrowed from anterior base posterad to ventral apex in ventral view, ventral apex straight to slightly concave. Pterothorax: scutellar shield with posterior apex pointed. Tarsal claw with ventral apex not smaller than dorsal apex. Male genitalia: paramere without preapical lateral expansion, with apical mesal callus present. Female: apex of last abdominal ventrite (ventrite V) simple, not emarginate at apex.

This species is unique in its longitudinal yellow elytral maculation, aedeagus with apical fourth of paramere compressed and gradually narrowing towards apex, and with apical mesal part of paramere turned ventrad (Fig. 6).

*Phorocardiusflavistriolatus* Ruan & Douglas, sp. nov. resembles *P.comptus* in having a longitudinal yellow stripe on each elytron. They can be separated by the following combination of characters: in *P.flavistriolatus* Ruan & Douglas, sp. nov., each elytron with a longitudinal yellow stripe covering basal half of interstria IV and interstriae V–VII, interstria VIII is entirely brown-black; female with pronotum not strongly enlarged, sides of pronotum only gently convex, width of pronotum to elytra ratio ca. 0.83–0.85, and proximal sclerites of bursa copulatrix with deep basal emargination; while in *P.comptus*, each elytron with a longitudinal yellow stripe covering interstriae V–VIII, interstria IV is entirely black; female with pronotum strongly enlarged, sides of pronotum strongly convex, width of pronotum to elytra ratio ca. 0.90–0.92 (measured in two specimens), and proximal sclerites of bursa copulatrix without basal emargination or with emargination narrower than 1/3 width of sclerite. The aedeagus of *P.flavistriolatus* Ruan & Douglas, sp. nov. has parameres with no apical or preapical expansion, with apical mesal callus present; while *P.comptus* has acute apical lateral expansions, without apical mesal callus.

*Phorocardiusflavistriolatus* Ruan & Douglas, sp. nov. is similar to *P.alterlineatus* Ruan & Douglas, sp. nov. in having yellow stripes on the elytra. However, they can be separated by the following characters: in *P.flavistriolatus* Ruan & Douglas, sp. nov., in lateral view, aedeagus with equal breadth from base to apical fifth, only slightly narrowed at apical fifth; apex of median lobe truncate to broadly rounded in ventral view; and each elytron with a single broad stripe covering basal half of interstria IV and interstriae V–VII; while in *P.alterlineatus* Ruan & Douglas, sp. nov., aedeagus gradually narrowed from base to apex in lateral view; apex of median lobe narrowly rounded in ventral view; and each elytron with three slender longitudinal stripes present separately on interstriae III, V, and VII, which partly merged near base and apex.

###### Description.

(based on all type specimens) Body brown-black, matt; antennae and legs paler, brown to yellow-brown. Head brown-black. Pronotum brown-black, with posterior edge brown. Scutellar shield brown-black. Elytra brown-black, each elytron with a longitudinal yellow stripe covering basal half of interstria IV and interstriae V to VII, epipleura orange at base, dark orange on remainder. Ventral surface entirely brown-black. Body with yellow-grey pubescence.

###### Measurements.

(based on all type specimens) Male body length 7.1–9.6 mm, width 2.2–2.9 mm. Female body length 8.0–11.0 mm, width 2.7–3.7 mm. Body length to width ratio 2.9–3.0. Pronotal width to length ratio 1.1–1.2. Pronotal width to body width ratio 0.82–0.85. Elytral length to pronotal length ratio 2.6–2.7; elytron length to width ratio 3.9–4.2.

***Head*.** Frons and vertex punctures with interspaces 0.5–1.0 × average puncture diameter; punctures sparser at centre of vertex. Frontal carina in frontal view convex, not straight. Distance between eyes to width of eye ratio 3.6–3.9. Antenna barely extending beyond posterior angle of pronotum. Antenna length to body length ratio, in male 0.39–0.41, in female 0.36–0.38. Proportions of antennomere lengths: 100 (scape); 52–60; 71–73; 71–73; 72–76; 72–76; 72–76; 73–78; 73–78; 73–78; 90–100.

***Prothorax*.** Pronotum in dorsal view: sides evenly convex from anterior edge to constriction near posterior fourth, nearly straight at posterior fourth, widest near posterior third; posterior angles with lateral sides almost straight, not bulged; surface with interspaces between punctures 1–2 × average puncture diameter. In ventral view, ventral surface of prosternal process with sides carinate and slightly and gradually narrow from anterior to mid-length, parallel from mid-length to apex, with apex slightly convex to almost straight. In lateral view, prosternal process with ventral surface curved slightly dorsad; posterior end weakly concave or not (Fig. 24B, upper arrow). Procoxal cavities open.

***Pterothorax*** (Figs 24B, 25B). Mesepisternum in ventral view with antero-mesal corner angulate (Fig. 25B, upper (green) arrow). Projections on posterior edge of mesosternum: in ventral view present (Fig. 25B, lower (red) arrow); in lateral view present, acute, strongly produced anteriorly (Fig. 24B, lower (red) arrow). Scutellar shield: width to length ratio 0.84–0.9; anterolateral edges slightly sinuate; posterior apex pointed. Elytra: upper edge of epipleura with minute serrations.

***Legs*.** Length ratio of metatarsomeres I–V (excluding claws): 100; 77–81; 65–69; 48–60; 121–123. Claw with ventral apex almost as large as dorsal apex.

***Abdomen*.** Lateral edges of visible abdominal ventrites I–V with minute serrations.

***Male genitalia*** (Fig. 4F–P). Robust from ventral and lateral views. Median lobe in ventral view with ridge along midline; narrowed from base to basal third, parallel-sided from basal third to apex, apex broadly rounded to truncate. Median lobe in lateral view curved ventrad from base to apex, apex rounded with angulate dorsal and ventral corners (Fig. 4P). Paramere in ventral view: robust, widest near mid-length, gradually narrowing towards apex, apical part with mesal side turned ventrad in varying degree (Fig. 6), result in different shapes in ventral view; apex with apical mesal callus present (Figs 4O, 6), without preapical lateral expansion; width 1.5–2.5 × median lobe width (measured at mid-length of paramere and median lobe respectively). Paramere in lateral view (Fig. 4N): robust; apical fourth compressed and turned ventrad; preapical ventral expansion obtuse, not sharp hook-shaped (Fig. 4P).

**Female.** Color pattern like male. Apex of abdominal ventrite V convex (Fig. 26B). Proximal sclerites of bursa copulatrix wide and somewhat diamond-shaped (Fig. 4Q), with concave basal edge, flat mesal edge and acute apex; each sclerite with seven or eight large spines arranged along mesal edge, and with 10–15 smaller scattered spines on disc.

###### Type material.

***Holotype***. male (SZPT), labels: 1) **Henan** Province, Nan-Yang City, Bao-tian-man National Nature Reserve (宝天曼国家自然保护区), Outward Bound Center, VIII-26-2015, leg. Mei-rong Liang, light trap, Shenzhen Polytechnic [in Chinese]; 2) Holotype, *Phorocardiusflavistriolatus* sp. nov., Des. Ruan et al., 2019.

***Paratypes*** (16♂, 11♀). 2♂ (IZCAS), labels: 1) **Sichuan**, Lu-ding County, Xin-xing township (新兴乡), 2000 m, 2009.VII.2–7, leg. Hua-kang Zhang, Chinese Academy of Sciences [in Chinese]; 2) Paratype, *Phorocardiusflavistriolatus* sp. nov., Des. Ruan et al., 2019. • 1♂ (LQCC), labels: 1) Lu-ding (泸定) (**Sichuan**), De-tuo township (得妥乡), 2015.8.6, QL, leg. Lu Qiu, Shenzhen Polytechnic [in Chinese]; 2) Paratype, *Phorocardiusflavistriolatus* sp. nov., Des. Ruan et al., 2019. • 2♂ (SZPT, ex. LQCC), labels: 1) Lu-ding (泸定) (**Sichuan**), Xin-xing township (新兴乡), 2016.VI.23, Q.X, leg. Jianyue Qiu & Hao Xu, Shenzhen Polytechnic [in Chinese]; 2) Paratype, *Phorocardiusflavistriolatus* sp. nov., Des. Ruan et al., 2019. • 4♂1♀ (SZPT), labels: 1) **Sichuan**, Shi-mian (石棉), Li-zi-ping National Nature Reserve, Zi-ma reserve station, VII-27-2016, leg. Li-ting Yu et al., Shenzhen Polytechnic [in Chinese]; 2) Paratype, *Phorocardiusflavistriolatus* sp. nov., Des. Ruan et al., 2019. • 1♂2♀ (SZPT), labels: 1) **Sichuan**, Shi-mian County (石棉), Li-zi-ping National Nature Reserve, Gong-yi-hai reserve station, Ma-ma-di, VII-24-2016, leg. Huang-qiang Liu, Shenzhen Polytechnic [in Chinese]; 2) Paratype, *Phorocardiusflavistriolatus* sp. nov., Des. Ruan et al., 2019. • 1♀ (SZPT), labels: 1) **Sichuan**, Bao-xing County (宝兴), Feng-tong-zai National Nature Reserve, Deng-chi-gou village, VIII-1-2016, leg. Huang-qiang Liu, Shenzhen Polytechnic [in Chinese]; 2) Paratype, *Phorocardiusflavistriolatus* sp. nov., Des. Ruan et al., 2019. • 2♂1♀ (IZCAS), labels: 1) **Sichuan**, Lu-ding County (泸定), Mo-xi township, 1500 m–1600 m, Chinese Academy of Sciences [in Chinese]; 2) 1983.VI.17–19 leg. Shuyong Wang & Xue-zhong Zhang [in Chinese]; 3) *Phorocardiuscomptus* Cand. Det. Ge; 4) Paratype, *Phorocardiusflavistriolatus* sp. nov., Des. Ruan et al., 2019. • 1♂ (SZPT), labels: 1) Gong-ga Mts. (**Sichuan**), Mo-xi (墨西), 2016.VI.26, leg. Chenglong Ren, SZPT [in Chinese]; 2) Paratype, *Phorocardiusflavistriolatus* sp. nov., Des. Ruan et al., 2019. • 1♂ (SZPT), labels: 1) **Shaanxi**, Ning-shan County (宁陕), Shi-ba-zhang-pu-bu Scenic Spot, 15.VIII.2013, leg. Jun Xu [in Chinese]; 2) Paratype, *Phorocardiusflavistriolatus* sp. nov., Des. Ruan et al., 2019. • 1♂ (IZCAS), labels: 1) **Shaanxi**, Fo-ping County (佛坪), alt.843 m, 2007.VIII.16, Chinese Academy of Sciences [in Chinese]; 2) 33.52°N, 107.98°E, leg. Yu-xia Yang, Chinese Academy of Sciences [in Chinese]; 3) IOZ(E)1882863; 4) Paratype, *Phorocardiusflavistriolatus* sp. nov., Des. Ruan et al., 2019. • 1♂1♀ (SZPT, ex. LQCC), labels: 1) **Henan**, Bai-yun-shan (白云山), Yu-huang-ding (玉皇顶), 1800–2200 m, 2016.8.12, leg. Weipeng Qiao, Shenzhen Polytechnic [in Chinese]; 2) Paratype, *Phorocardiusflavistriolatus* sp. nov., Des. Ruan et al., 2019. • 5♀ (SZPT, ex. LQCC), labels: 1) Nei-xiang (内乡县) (**Henan** prov.), Bao-tian-man (宝天曼), 1200–1400 m, 2016.8.15–20, leg. Qiaozhi Yang & Weipeng Qiao, Shenzhen Polytechnic [in Chinese]; 2) Paratype, *Phorocardiusflavistriolatus* sp. nov., Des. Ruan et al., 2019.

###### Remarks.

Although there are variations in the shape of aedeagi, all the male specimens are identical in color patterns and other external structures. Females are stable in both external characters and the shape of sclerites of bursa copulatrix. Variations are found even in the specimens collected at the same place and time. For instance, Fig. 4G, H shows aedeagi of two externally identical males collected on the same day and same locality in Shi-mian, Sichuan; the two aedeagi are different from each other in the basal width and the shape of the apex of paramere. In Fig. 4J, K, these are aedeagi of two males collected on the same date and same locality in Lu-ding County, Sichuan; the two aedeagi are different from each other in the basal width, median lobe shape, and the shape of the apex of paramere. Additional mitochondrial DNA sequence comparisons would be useful to further test species limits.

Variation of the shape of aedeagus: in some cases, the base of the aedeagus is much narrower (e.g., Fig. 4G, J). Variations in the paramere: apical half of paramere of aedeagus compressed; additionally, apical part with mesal side turned ventrad to a varying degree (Fig. 6), resulting in different shapes in ventral view. In ventral view, when the turning is minimal, the apical part of paramere is nearly horizontal to the observers, therefore the paramere apex is broad and gradually narrowed in appearance (e.g., Fig. 4F, G); but, when the turning is stronger, the paramere apex would be nearly vertical to the observers, in which circumstance the paramere apex we see is acute and abruptly narrowed (e.g., Fig. 4K, M). Variations in the median lobe: in ventral view, apex usually truncate, in rare cases broadly rounded (Fig. 4K, L), lateral sides very slightly concave in rare cases (Fig. 4K).

Most specimens of this species were collected from low to middle elevations (ca. 500–2500 m) in central China on the boundary of the Oriental Palearctic Regions. Based on collecting information, it inhabits mountainous areas with evergreen forest and temperate to subtropical climate. Specimens were collected at light traps, indicating nocturnal activity. Specimens collected by sweep-netting indicate their presence on vegetation during daylight.

##### 3. Phorocardius florentini

Taxon classificationAnimaliaColeopteraElateridae

(Fleutiaux, 1895)

0C605CE9-F7EB-5C11-81B6-0B41959B80D7

**Figure 7.**
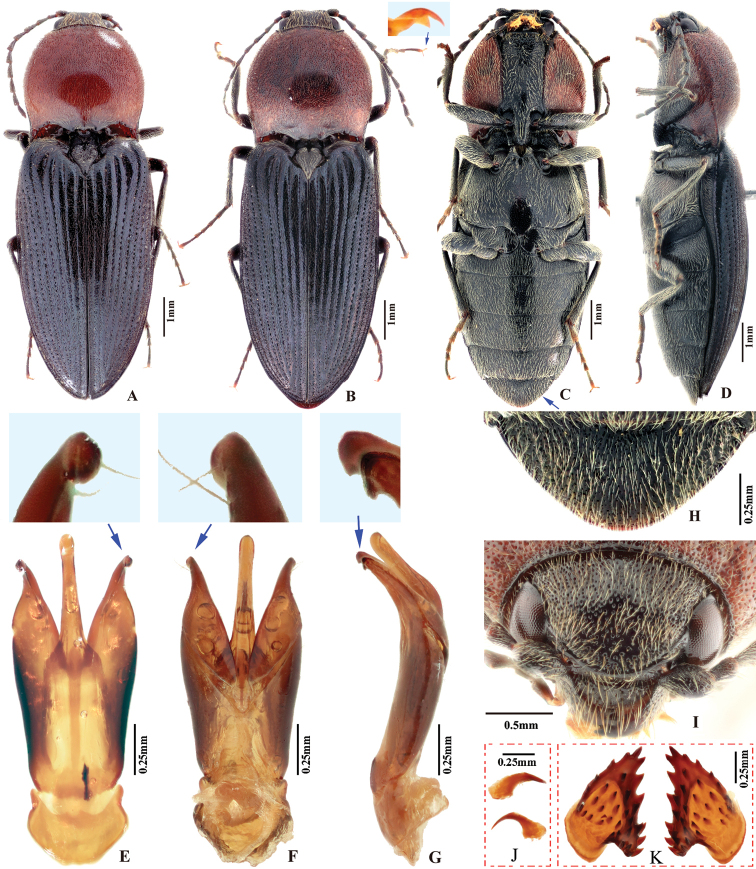
*Phorocardiusflorentini* (Fleutiaux, 1895). **A** male habitus, dorsal view **B** female habitus, dorsal view, arrow indicating claw **C** female habitus, ventral view, arrow indicating last visible ventrite **D** female habitus, lateral view **E** aedeagus, ventral view, arrow indicating preapical lateral expansion **F** aedeagus, dorsal view, arrow indicating preapical lateral expansion **G** aedeagus, lateral view, arrow indicating preapical ventral expansion **H** last visible ventrite of female, ventral view **I** head, frontal view **J** distal sclerites of bursa copulatrix **K** proximal sclerites of bursa copulatrix.

Figs 7 , 23C , 24C , 25C , 26C

Cardiophorus florentini Fleutiaux, 1895: 687. Type locality: “Tonkin: Lang-son”, interpreted as Vietnam: Lạng Sơn Province.
Phorocardius florentini : Fleutiaux 1931: 311.

###### Distribution.

China (Guizhou, new record); Vietnam (Fleutiaux 1895).

###### Differential diagnosis.

Body length greater than 7.0 mm; pronotum and hypomeron red, elytra black with metallic blue to purple luster. Prothorax: procoxal cavities open; prosternal process not strongly narrowed from anterior base posterad to ventral apex in ventral view, with apex convex. Pterothorax: scutellar shield elongate, with posterior apex pointed. Tarsal claw with ventral apex not smaller than dorsal apex. Male genitalia: paramere with preapical lateral expansion present, apical mesal callus absent. Female: apex of last abdominal ventrite (ventrite V) simple, not emarginate at apex.

This species is distinctive for its elytral color: black with metallic blue to purple luster.

*Phorocardiusflorentini* (Fleutiaux, 1895) resembles *P.zhiweii* Ruan, Douglas & Qiu, sp. nov. in its entirely red pronotum and metallic elytra. *P.florentini* (Fleutiaux, 1895) can be easily separated from *P.zhiweii* Ruan, Douglas & Qiu, sp. nov. by the following characters. In *P.florentini* (Fleutiaux, 1895): aedeagus strongly narrowed from mid-length to apex in lateral view; in dorsal view, paramere with preapical lateral expansion minute, acute to rounded, facing laterally, with apical mesal callus absent; scutellar shield elongate (width to length ratio: 0.81–0.86); and elytra black, with metallic blue to purple luster; while in *P.zhiweii* Ruan, Douglas & Qiu, sp. nov., the aedeagus is only slightly narrowed from mid-length to apex in lateral view; in dorsal view, paramere with preapical lateral expansion absent, apical mesal callus present, apex narrow and slightly bent laterad; scutellar shield not elongate (width to length ratio: 1.0); and elytra metallic green with slight purple luster.

###### Description.

(Based on holotype and three non-type specimens examined) Body black, red and metallic blue-purple (Fig. 7A–D). Pronotum and hypomera red. Elytra black, with metallic blue to purple luster. Head brown-black to black; antennae brown-black. Prosternum red, black or mixed with red and black; meso- and meta- sternum black; abdominal ventrites black; legs brown-black to black. Body with short, yellow pubescence; brown setae also present on disc of pronotum.

###### Measurements.

(based on type and non-type specimens) Male body length 9.0–11.5 mm, width 3.3–3.8 mm. Female body length 10.0–13.0 mm, width 3.7–4.0 mm. Body length to width ratio 2.6–2.8. Pronotal width to length ratio 1.1–1.2, Pronotal width to body width ratio 0.86–0.91. Elytral length to pronotal length ratio 2.3–2.5; elytron length to width ratio 3.7–3.9.

***Head*.** Frons and vertex punctures with interspaces 0.5–1 × average puncture diameter; punctures sparser at centre of vertex, with interspaces 1.5–2 × average puncture diameter. Frontal carina in frontal view convex, not straight (Fig. 7I). Antenna with last antennomere not reaching beyond posterior angle of pronotum. Distance between eyes to width of eye ratio 3.8–3.9. Antenna length to body length ratio, in male 0.36–0.38; in female 0.37–0.39. Proportions of antennomere lengths (male): 100 (scape); 60–65; 75–83; 75–83; 75–83; 78–85; 81–86; 85–90; 85–90; 81–85; 90–100.

***Prothorax*.** Pronotum in dorsal view: sides evenly convex from anterior edge to constriction near posterior end, widest near mid-length; posterior angles with lateral sides almost straight, not bulged; surface with interspaces between punctures 1–2 × average puncture diameter. In ventral view, ventral surface of prosternal process with sides carinate and slightly and gradually narrow from anterior to mid-length, parallel from mid-length to posterior end, with apex convex. In lateral view, prosternal process with ventral surface curved slightly dorsad, posterior end somewhat concave or not (Fig. 24C, upper arrow). Procoxal cavities open.

***Pterothorax*** (Figs 24C, 25C). Mesepisternum in ventral view with antero-mesal corner angulate (Fig. 25C, upper (green) arrow). Projections on posterior edge of mesosternum: in ventral view present (Fig. 25C, red arrow); in lateral view present, acute, strongly produced anteriorly (Fig. 24C). Scutellar shield: elongate, width to length ratio 0.81–0.86; anterolateral edges slightly sinuate; posterior edge gradually narrowed and elongate, strongly protruding posterad, pointed at apex. Elytra: upper edge of epipleura with minute serrations.

***Legs*.** Length ratio of metatarsomeres I–V (excluding claws): 100; 80–85; 70–75; 50–55; 135–140. Claw with ventral apex almost as large as dorsal apex.

***Abdomen*.** Lateral edges of visible abdominal ventrites I–V with minute serrations.

***Male genitalia*** (Fig. 7E–G). Robust from ventral and lateral views. Median lobe in ventral view slightly narrowed from base to basal third, parallel-sided from basal third to near apex, apex rounded and very slightly dilated. Median lobe in lateral view wide from base to mid-length, narrow from mid-length to apex; gently and evenly curved ventrad from base to mid-length, straight from mid-length to apex; apex rounded. Paramere in ventral view: wide and equal wide from base to apical third; abruptly narrowed from apical third to apex; preapical lateral expansion present, minute and rounded, facing laterally; apical mesal callus absent; paramere width 3–4 × median lobe width (measured at mid-length of paramere and median lobe respectively). Paramere in lateral view: robust, almost straight from base to mid-length, curved ventrad and narrowed from mid-length to apex; apex with hook-like preapical expansion with barb facing base.

**Female.** Body color like male (Fig. 7B). Apex of abdominal ventrite V convex (Fig. 7H). Proximal sclerites of bursa copulatrix wide with apex acute, base concave, and mesal edge flat (Fig. 7K); each with 8–10 large spines on mesal edge, 14–18 smaller spines on disc.

###### Type material.

***Lectotype***. ♀ (MNHN), labels: 1) Tonkin Florentin; 2) Type [red label]; 3) Museum Paris Coll. E. Fleutiaux; 4) *Cardiophorusflorentini* Fleut. Type; 5) Fleut Ann. Soc. Ent. Fr., 1894. P. 687, Collection Fleutiaux; 6) *C.florentini* Fleut., type, Collection Fleutiaux; 7) Lectotype *Cardiophorusflorentini* Fleutiaux desig. Douglas 2015.

###### Additional material.

2♂1♀ (SZPT, ex. LQCC), labels: 1) **Guizhou**, Li-bo, Mao-lan, Dong-duo, 2000 m, 2018.VI.11–17, leg. Jianyue Qiu & Hao Xu [in Chinese]; 2) *Phorocardiusflorentini* (Fleutiaux, 1894) Det. Ruan, 2019.

###### Remarks.

Based on examined material, this species inhabits low to middle elevations (0–2000 m) in south China and north Vietnam. Recent Chinese specimens were collected in daylight in a mountainous area with evergreen forest and subtropical climate. Known from the Oriental Region only.

##### 4. Phorocardius magnus

Taxon classificationAnimaliaColeopteraElateridae

Fleutiaux, 1931

E3ED0F8A-F3D0-5172-8DB0-35BD1E988AB8

**Figure 8.**
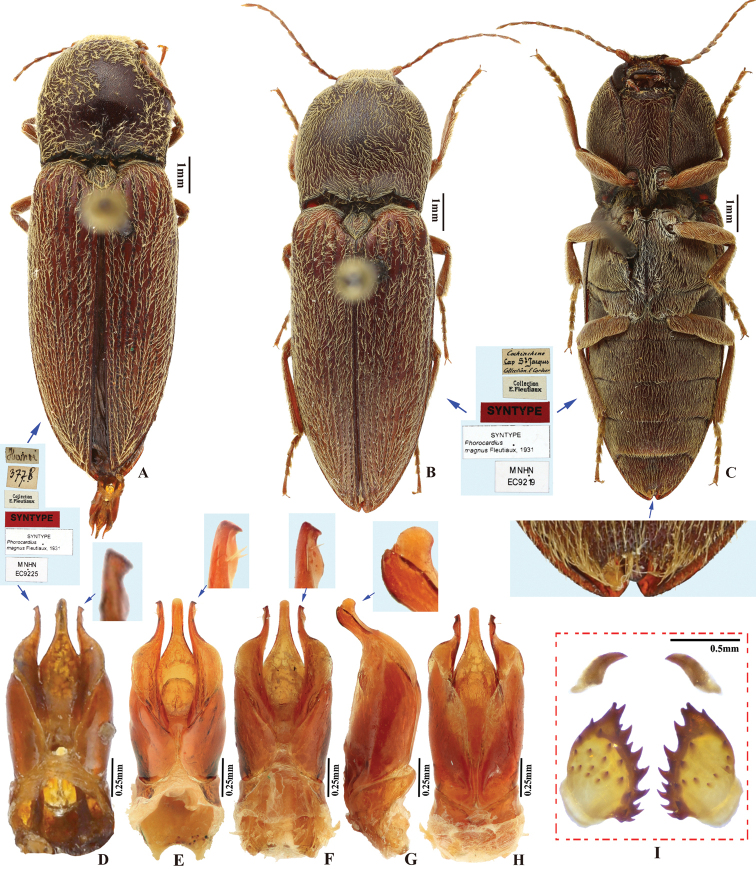
*Phorocardiusmagnus* Fleutiaux, 1931. **A** lectotype, male, from Hanoi, Vietnam, dorsal view (photograph: Dr Antoine Mantilleri, MNHN), arrow indicating specimen labels **B** paralectotype, female, from Vung Tau, Vietnam, dorsal view (photograph: Dr Antoine Mantilleri, MNHN), arrow indicating specimen labels **C** the same female as **B**, ventral view (photograph: Dr Antoine Mantilleri, MNHN), arrows indicating specimen labels and last visible ventrite **D** aedeagus of lectotype, dorsal view (photograph: Dr Antoine Mantilleri, MNHN), arrows indicating specimen labels and apex of paramere **E** aedeagus of non-type specimen, individual-1 (Yunnan, Xi-shuang-ban-na, Meng-zhe), dorsal view, arrow indicating apex of paramere **F** aedeagus of non-type specimen (Yunnan, Jing-dong, Dong-jia-feng), individual-2, dorsal view, arrow indicating apex of paramere **G** aedeagus, individual-2, lateral view, arrow indicating apex of paramere **H** aedeagus, individual-2, ventral view **I** distal (upper side) and proximal sclerites of bursa copulatrix, non-type specimen.

**Figure 9.**
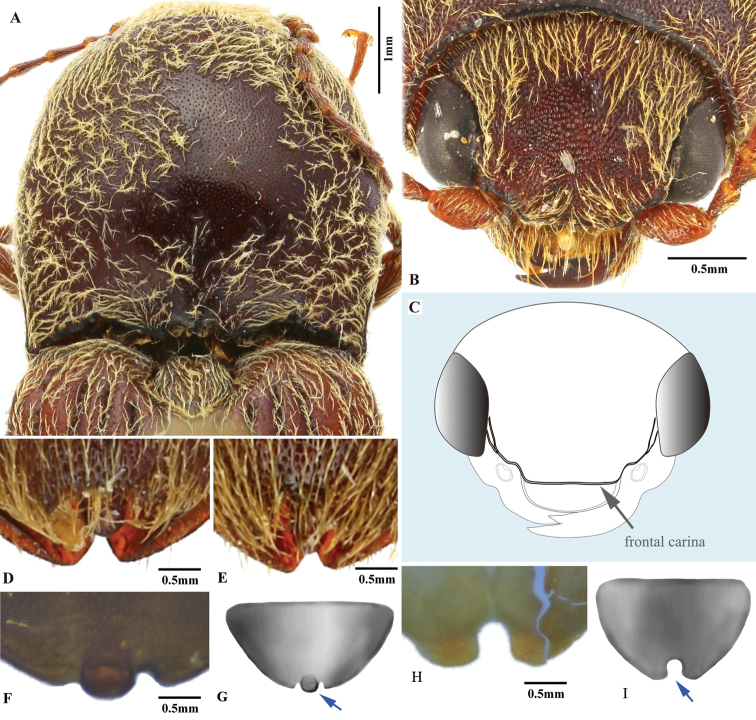
*Phorocardiusmagnus* Fleutiaux, 1931. **A** pronotum of lectotype, dorsal view (photograph: Dr Antoine Mantilleri, MNHN) **B** head of paralectotype, frontal view (photograph: Dr Antoine Mantilleri, MNHN) **C** head, frontal view, hand drawing, arrow indicating straight frontal carina **D, E** apex of last visible abdominal ventrite (ventrite V) of female, ventral view, paralectotype (photograph: Dr Antoine Mantilleri, MNHN) **F** apex of last visible abdominal ventrite (ventrite V) of female, dorsal view, non-type specimen **G** a model of last visible abdominal ventrite (ventrite V) of female, ventral view, hand drawing, arrow indicating tri-lobed apex **H** apex of last visible abdominal tergite (tergite VII) of female, dorsal view, non-type specimen **I** a model of last visible abdominal tergite (tergite VII) of female, dorsal view, hand drawing, arrow indicating concave apex.

Figs 8 , 9 , 23D , 24D , 25D , 26D

Phorocardius magnus Fleutiaux, 1931: 310. Type locality: Vietnam (Hanoi). Lectotype designated here.

###### Distribution.

China (Yunnan, Hainan), Vietnam (Fleutiaux 1931).

###### Differential diagnosis.

Body length greater than 7.0 mm; integument entirely red-brown to brown throughout. Prothorax: procoxal cavities open; prosternal process gradually narrowed posterad to ventral apex in ventral view, with apex narrowly rounded. Pterothorax: scutellar shield with posterior apex pointed. Tarsal claw with ventral apex not smaller than dorsal apex. Male genitalia: paramere with preapical lateral expansion and secondary lateral bulge present, apical mesal callus absent. Female: apex of last abdominal ventrite (ventrite V) tri-lobed, emarginate between middle and lateral lobes.

*Phorocardiusmagnus* Fleutiaux, 1931 resembles *P.unguicularis* (Fleutiaux, 1918) in body color and size. They can be separated by the following combination of characters: in *P.magnus* Fleutiaux, 1931, aedeagus robust in lateral view (more than 4 × thicker at mid-length than at apical 1/5 of parameres); in ventral view, paramere slightly narrowed from base to apical fourth, apical fourth abruptly narrowed to 1/4 width at mid-length, with hook-like preapical lateral expansion; pronotum with shallow punctures, interspaces between punctures 1–2.5 × average puncture diameter; and head with frontal carina straight in frontal view; while *P.unguicularis* (Fleutiaux, 1918) has aedeagus slender in lateral view, in ventral view paramere slightly widened from base to mid-length, gradually narrowed from mid-length to apex, apex pointed and without preapical lateral expansion; pronotum with deep punctures, interspaces between punctures 0.3–1 × average puncture diameter; and head with frontal carina convex in frontal view.

###### Description.

(based on photographs of eight type specimens and four non-type specimens) Body robust. Color entirely red-brown to brown throughout, including legs and antennae; pronotum slightly darker than remainder. Integument matt, with yellow pubescence.

###### Measurements.

(based on lectotype and examined specimens) Male body length 9.5–12.0 mm, width 3.0–4.1 mm. Female body length 11.0–13.9 mm, width 3.9–4.5 mm. Body length to width ratio 2.9–3.2. Pronotal width to length ratio 1.0–1.2. Pronotal width to body width ratio 0.90–0.97. Elytral length to pronotal length ratio 2.5–2.7; elytron length to width ratio 3.9–4.1.

***Head*.** Frons and vertex with interspaces between punctures 0.3–1 × average puncture diameter (Fig. 9B). Frontal carina in frontal view transversely straight (Fig. 9C). Antenna with apex extending to posterior angle of pronotum. Distance between eyes to width of eye ratio 2.9–3.1. Antenna length to body length ratio, in male 0.35–0.36, in female 0.32–0.33. Proportions of antennomere lengths (male): 100 (scape); 46–53; 57–63; 55–58; 50–55; 59–60; 59–65; 57–63; 60–63; 60–67; 80–87.

***Prothorax*.** Pronotum in dorsal view: robust, comparatively larger than other Chinese *Phorocardius* species, with sides evenly convex, widest near mid-length, straighter posterad; posterior angles with lateral sides straight to slightly convex, not bulged; surface with shallow punctures, interspaces between punctures 1–2.5 × average puncture diameter. In ventral view, ventral surface of prosternal process with sides carinate and gradually narrow from anterior to posterior end, with apex narrowly rounded. In lateral view, prosternal process with ventral surface curved slightly dorsad, posterior end somewhat concave (Fig. 24D, upper arrow). Procoxal cavities narrowly open.

***Pterothorax*** (Figs 24D, 25D). Mesepisternum in ventral view with antero-mesal corner narrowly rounded (Fig. 25D, upper (green) arrow). Projections on posterior edge of mesosternum: in ventral view present (Fig. 25D, lower (red) arrow); in lateral view present, acute, strongly produced anteriorly (Fig. 24D, lower (red) arrow). Scutellar shield: width to length ratio 0.92–1.10; anterolateral edges slightly sinuate; posterior apex pointed. Elytra: upper edge of epipleura with very weak minute serrations (barely visible).

***Legs*.** Length ratio of metatarsomeres I–V (excluding claws): 100; 90–98; 70–79; 61–75; 145–152. Claw with ventral apex almost as large as dorsal apex.

***Abdomen*.** Lateral edges of visible abdominal ventrites I–V with minute serrations. Male genitalia. Robust from ventral and lateral views (Fig. 8). Median lobe in ventral view gradually narrowing from base to apical third, parallel-sided near rounded apex. Median lobe in lateral view robust at base, curved ventrad and gradually narrowed from base to apical third, apical third slender, apex globose and recurved dorsad (Fig. 8G, indicated by arrow). Paramere in ventral view: robust, widest near mid-length (4–5 × wider than at apical fifth); width 1.5–2.5 × median lobe width (measured at mid-length part of paramere and median lobe respectively); apical fourth gradually narrowing towards apex, with a secondary lateral bulge present before apex (Fig. 8D–F, indicated by arrow); secondary lateral bulge turned and bent ventrad; preapical lateral expansion small, sharp, hook like; apical mesal callus absent. Paramere in lateral view: robust; parallel from base to mid-length, abruptly narrowed from mid-length to apical fourth, nearly parallel from apical fourth to apex; preapical ventral expansion absent, without hook-shaped structure.

**Female.** Color like male. Apex of abdominal ventrite V tri-lobed; with shape of middle lobe semicircular to longitudinal with apex rounded; deeply to gently incised between middle and lateral lobes (Fig. 9D–G). [In non-type specimens: proximal sclerites of bursa copulatrix ovoid (Fig. 8I), with shallow basal concavity; apex acute, each with 8–11 large spines on convex mesal edge, 15–17 smaller spines on disc.]

###### Type material.

(all in MNHN) (photographs provided by Dr Antoine Mantilleri). ***Lectotype***. ♂, labels: 1) Hanoi; 2) 3778; 3) Collection E. Fleutiaux; 4) Syntype [red label]; 5) Syntype *Phorocardiusmagnus* Fleutiaux, 1931; 6) MNHN EC9225; 7) Lectotype, *Phorocardiusmagnus* Fleutiaux, 1931, Des. Ruan & Douglas, 2019.

###### Paralectotypes.

1♀, labels: 1) Cochinchine, Cap St. Jacques, Collection E Cordier; 2) *Phorocardiusmagnus* Fleut., type, Collection Fleutiaux; 3) Collection E. Fleutiaux; 4) Syntype [red label]; 5) Syntype *Phorocardiusmagnus* Fleutiaux, 1931; 6) MNHN EC9218; 7) Paralectotype, *Phorocardiusmagnus* Fleutiaux, 1931, Des. Ruan & Douglas, 2020. • 1♀, labels: 1) Cochinchine, Cap St. Jacques, Collection E Cordier; 2) Collection E. Fleutiaux; 3) Syntype [red label]; 4) Syntype *Phorocardiusmagnus* Fleutiaux, 1931; 5) MNHN EC9219; 6) Paralectotype, *Phorocardiusmagnus* Fleutiaux, 1931, Des. Ruan & Douglas, 2020. • 1 (sex unknown), labels: 1) Cochinchine, Cap St. Jacques, Collection E Cordier; 2) Collection E. Fleutiaux; 3) Syntype [red label]; 4) Syntype *Phorocardiusmagnus* Fleutiaux, 1931; 5) MNHN EC9220; 6) Paralectotype, *Phorocardiusmagnus* Fleutiaux, 1931, Des. Ruan & Douglas, 2020. • 1 (sex unknown), labels: 1) Cochinchine, Cap St. Jacques, Collection E Cordier; 2) Collection E. Fleutiaux; 3) Syntype [red label]; 4) Syntype *Phorocardiusmagnus* Fleutiaux, 1931; 5) MNHN EC9221; 6) Paralectotype, *Phorocardiusmagnus* Fleutiaux, 1931, Des. Ruan & Douglas, 2020. • 1♀, labels: 1) Cochinchine, Cap St. Jacques, Collection E Cordier; 2) type, ……[characters illegible] Collection Fleutiaux; 3) Collection E. Fleutiaux; 4) ex Coll Fleut., *Phorocardiusmagnus*. 5) Syntype [red label]; 6) Syntype *Phorocardiusmagnus* Fleutiaux, 1931; 6) MNHN EC9222; 7) ♀ genitalia See slide Coll No. 105; 8) Paralectotype, *Phorocardiusmagnus* Fleutiaux, 1931, Des. Ruan & Douglas, 2020. • 1 (sex unknown), labels: 1) Dap Cau, 1 au 9. 7. 06 [= 1 to 9 July, 1906]; 2) Collection E. Fleutiaux; 3) *Phorocardiusmagnus* Fleut., Collection E. Fleutiaux; 4) Syntype [red label]; 5) Syntype *Phorocardiusmagnus* Fleutiaux, 1931; 6) MNHN EC9223; 7) Paralectotype, *Phorocardiusmagnus* Fleutiaux, 1931, Des. Ruan & Douglas, 2020. • 1 (sex unknown), label: 1) Dap Cau, 1 au 9. 7. 06’ [= 1 to 9 July, 1906]; 2) Collection E. Fleutiaux; 3) Syntype [red label]; 4) Syntype *Phorocardiusmagnus* Fleutiaux, 1931; 5) MNHN EC9224; 6) Paralectotype, *Phorocardiusmagnus* Fleutiaux, 1931, Des. Ruan & Douglas, 2020.

###### Additional material.

One female and two males. 1♀ (IZCAS), labels: 1) **Hainan** Prov., Na-da (那大), Beijing Natural History Museum, leg. Sikong Liu, 1964.V.14 [in Chinese]; 2) *Phorocardiusmagnus* Fleut. Det. Siqin Ge; 3) *Phorocardiusmagnus* Fleutiaux, 1931 Det. Ruan, 2018. • 1♂ (IZCAS), labels: 1) **Yunnan**, Xi-shuang-ban-na, Meng-zhe (勐遮), 1700 m, Chinese Academy of Sciences [in Chinese]; 2) 1958.IV.22, leg. Shuyong Wang [in Chinese]; 3) *Phorocardiusmagnus* Fleutiaux, 1931 Det. Ruan, 2018. • 1♂ (IZCAS), labels: 1) **Yunnan**, Jing-dong, Dong-jia-feng (董家坟), 1250 m, 1956.VI.2; 2) *Phorocardiusmagnus* Fleutiaux, 1931 Det. Ruan, 2018.

###### Remarks.

This species has the largest body size in Chinese *Phorocardius* species, with females up to 13.9 mm long and 4.5 mm wide.

Based on specimen information, this species inhabits low to middle elevations (0–1700 m) in south China and throughout Vietnam. It inhabits mountainous areas with subtropical to tropical climates and much rainfall. One specimen was collected in or near to tropical rain forest (“Xi-shuang-ban-na tropical rain forest”). Known from the Oriental Region only.

##### 5. Phorocardius manuleatus

Taxon classificationAnimaliaColeopteraElateridae

(Candèze, 1888)

073F6FA9-B02D-5907-BA00-70D580B56200

**Figure 10.**
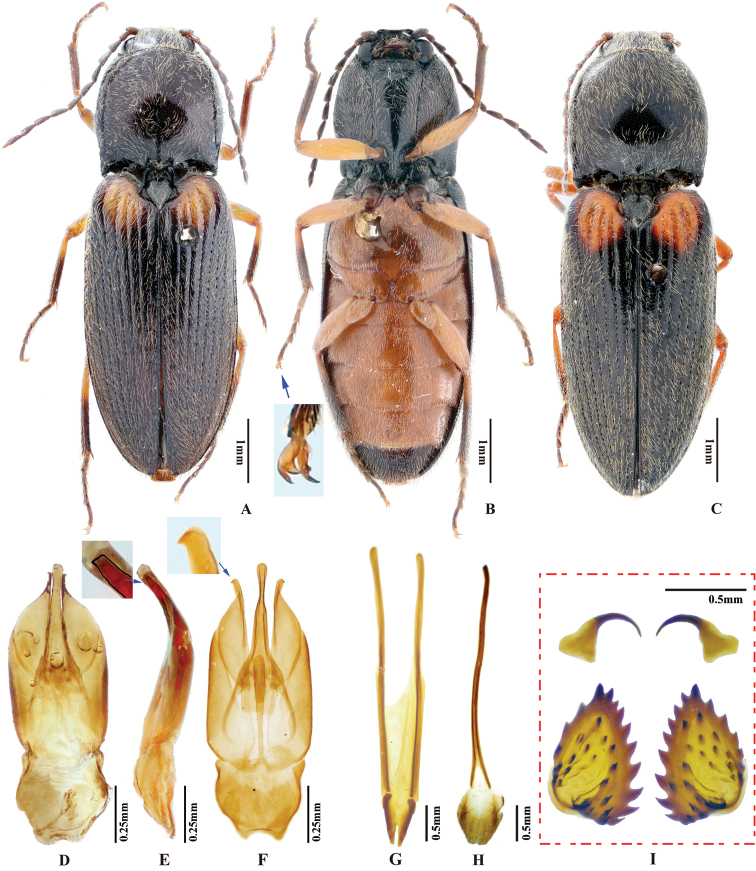
*Phorocardiusmanuleatus* Candèze, 1888. **A** male habitus, dorsal view **B** male habitus, ventral view, arrow indicating claws **C** female habitus, dorsal view **D** aedeagus, ventral view (immersed in glycerin) **E** aedeagus, lateral view, arrow indicating apex of paramere **F** aedeagus, dorsal view (immersed in glycerin), arrow indicating apex of paramere **G** ovipositor, dorsal view **H** female abdominal sternite VIII, dorsal view (near type locality) **I** distal (top) and proximal sclerites of bursa copulatrix.

**Figure 11.**
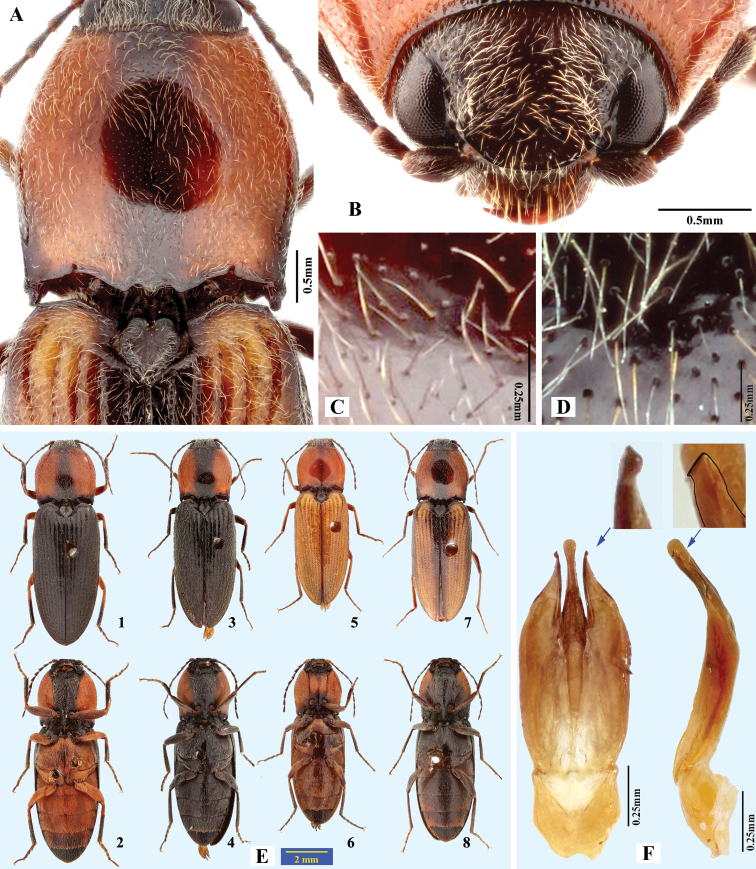
*Phorocardiusmanuleatus*Candèze (1888). **A** pronotum and scutellar shield, dorsal view **B** head, frontal view **C** punctation on disc of pronotum **D** punctation on vertex of head **E** color variation between four individuals; insets 2, 4, 6, 8 are the ventral views of 1, 3, 5, 7 **F** aedeagus, in dorsal view and lateral view, arrows indicating apices of parameres.

Figs 10 , 11 , 23E , 24E , 25E , 26E

Cardiophorus manuleatus Candèze, 1888: 681. Type locality: “Thagatà, Tenasserim”, interpreted as Myanmar, Kayin State, mountains east of Kyaikdon using Hallermann et al. (2002). Lectotype designated here.
Phorocardius melanopterus manuleatus : Fleutiaux 1931: 311.
Phorocardius manuleatus : Fleutiaux 1947: 366.

###### Differential diagnosis.

Body length greater than 7.0 mm; integument shiny, black with yellow in most, entirely black to black-brown in some. Prothorax: procoxal cavities open; prosternal process strongly narrowed posterad to ventral apex in ventral view, with apex acute. Pterothorax: scutellar shield with posterior apex pointed. Tarsal claw with ventral apex not smaller than dorsal apex. Male genitalia: paramere with preapical lateral expansion present, without apical mesal callus. Female: apex of last abdominal ventrite (ventrite V) simple, not emarginate at apex.

*Phorocardiusmanuleatus* (Candèze, 1888) is unique among Chinese *Phorocardius* species for its variable color pattern. Some individuals are entirely black to black-brown throughout body, resembling *P.yanagiharae* (Miwa, 1927) and *P.yunnanensis* sp. nov.

This species can be differentiated from *P.yanagiharae* by the following combination of characters. In *P.manuleatus*: in ventral view, parameres of aedeagus with sides gently narrowed from mid-length to apex (not abruptly narrowed from apical third to near apex), with width 1.5–2 × that of median lobe (measured at apical fourth); and in dorsal view, pronotum with lateral sides of posterior angles almost straight, slightly convex (bulged) at posterior half in a few cases (e.g., in Fig. 11A); while in *P.yanagiharae*, in ventral view, paramere of aedeagus with sides abruptly narrowed from apical third to near apex, with width 2–3 × that of median lobe (measured at apical fourth); and in dorsal view, pronotum with lateral sides of posterior angles strongly bulged and convex (Fig. 18C, D).

This species can be differentiated from *P.yunnanensis* Ruan & Douglas, sp. nov. by the following combination of characters. In *P.manuleatus*, in ventral view, paramere of aedeagus narrow and slender near apex, with width to that of median lobe ratio 0.5–0.7 (measured at the area posterior of preapical lateral expansion); legs darker in apical half, not unicolor yellow-brown to brown; while in *P.yunnanensis* Ruan & Douglas, sp. nov., in ventral view, paramere of aedeagus wide and strong near apex, with width to that of median lobe ratio 1.0–1.2 (measured at the area posterior of preapical lateral expansion); and legs unicolor, entirely yellow-brown.

###### Distribution.

China (Yunnan, new record), Myanmar (Candèze 1888), Laos (Fleutiaux 1931, 1947), Vietnam (Fleutiaux 1918, 1947).

###### Description.

(based on lectotype and 24 non-type specimens) Integument shiny, black with yellow in most, or entirely black to black-brown. Pronotum entirely black or orange with variable median black stripe (Fig. 11E). Ventral side of prothorax yellow, orange, black or orange with black prosternum. Elytra black, yellow, or black with orange spot at elytral bases. Mesosternum brown to black. Metasternum yellow to black. Abdominal ventrites yellow, black, or bicolored (I–IV orange, V yellow). Head red-brown to black. Antennae brown. Legs variably orange to yellow-brown from coxa to mid tibia, yellow-brown to brown from mid tibia to last tarsomere. Body with yellow pubescence.

###### Measurements.

(based on lectotype and examined specimens) Male body length 7.2–9.6 mm, width 2.2–2.6 mm. Female body length 8.5–9.7 mm, width 2.5–2.9 mm. Body length to width ratio 3.0–3.1. Pronotal width to length ratio 1.1–1.2. Pronotum narrower than elytra, pronotal width to body width ratio 0.87–0.90. Elytral length to pronotal length ratio 2.4–2.6; elytron length to width ratio 4.1–4.2.

***Head*.** Frons and vertex with interspaces between punctures 2.5–6 × average diameter of puncture (Fig. 11D). Frontal carina in frontal view convex, not straight. Antenna with apex extending to posterior angle of pronotum. Distance between eyes to width of eye ratio 2.7–3.1. Antenna length to body length ratio, in male 0.41–0.42, in female 0.39–0.40. Proportions of antennomere lengths (male): 100 (scape); 51–55; 72–80; 75–82; 82–88; 82–84; 72–78; 72–78; 80–88; 80–89; 114–120.

***Prothorax*.** Pronotum in dorsal view: sides convex near mid-length, nearly straight at ends, widest near mid-length; posterior angles with lateral sides almost straight, slightly convex (bulged) at basal half in a few cases (e.g., Fig. 11A); surface with interspaces between punctures 4–8 × average puncture diameter (Fig. 11C). In ventral view, ventral surface of prosternal process with sides carinate and strongly narrowed from anterior to posterior end, with apex acute. In lateral view, prosternal process with ventral surface curved slightly dorsad, posterior end strongly concave (Fig. 24E, upper arrow). Procoxal cavities open.

***Pterothorax*** (Figs 24E, 25E). Mesepisternum in ventral view with antero-mesal corner angulate mesad of a notch (Fig. 25E, upper (green) arrow). Projections on posterior edge of mesosternum: in ventral view present (Fig. 25E, lower (red) arrow); in lateral view present, acute, strongly produced anteriorly (Fig. 24E, lower (red) arrow). Scutellar shield: width to length ratio 1.0, anterolateral edges slightly sinuate, posterior apex pointed. Elytra: upper edge of epipleura with minute serrations.

***Legs*.** Length ratio of metatarsomeres I–V: 100; 82–92; 67–75; 60–70; 155–180. Claw with ventral apex almost as large as dorsal apex.

***Abdomen*.** Serrations on lateral edges of visible abdominal ventrites I–V absent.

***Male genitalia*.** Robust in ventral view, slender in lateral view. Median lobe in ventral view gradually narrowing from base to near apex, then dilated to rounded apex. Median lobe in lateral view curved ventrad at base, straight from basal third to apex; apex broadly rounded. Paramere in ventral view: robust, width 3–4 × median lobe width (measured at mid-length of paramere and median lobe respectively), widest near mid-length; apical fourth gradually narrowing towards apex, with mesal side bent and turned ventrad in varying degree, result in slightly different shapes in ventral view; apex of paramere slender and sharp, with preapical lateral expansion acute, hook-like to rounded, facing laterally (Figs 10F, 11F, indicated by blue arrow), without apical mesal callus. Paramere in lateral view: slender, almost straight from base to mid-length, curved ventrad and gradually narrowed from mid-length to apex; apex obliquely truncate; preapical ventral expansion acute but not hook-like (Figs 10E, 11F, indicated by blue arrows).

**Female.** Body color like male. Apex of abdominal ventrite V convex, somewhat angulate (Fig. 26E). Proximal sclerites of bursa copulatrix ovoid-triangular shaped (Fig. 10I), basal edge almost without concavity: each with 9–11 large spines mainly on the convex mesal edge, 15–20 smaller spines on disc.

###### Type material.

***Lectotype***. ♂ (RBINS): 1) Coll. R. I. SC. N. B., Inde; 2) Tenasserim, Thagatà, Fea. Apr. 1887; 3) Collection E. Candèze; 4) *Manuleatus* cdz., Tenasserim; 5) *Cardiophorusmanuleatus*, Cd., dèt. E. Candèze; 6) Probably syntype var. a., Det. W. Suzuki, 1986; 7) Lectotype, *Cardiophorusmanuleatus* Candèze, 1888, Des. Ruan & Douglas, 2020.

###### Additional material.

1♂ (NHMUK), labels: 1) Cotype; 2) Carin Chebà, 900–1100 m, L. Fea, V XII-88; 3) Andrewes Bequest. B. M. 1922-221. 4) *Cardiophorusmanuleatus* Cand. Co.type.; 5) Not paratype of *manuleatus* Cand., wrong loc., C.M.F. von Hayek. det., 1957. [Notes: locality of this specimen (“Chebà”) differs from what Candèze (1888) provided (“Thagatà, Tenasserim”). Although “Thagatà” and “Chebà” are both in “Carin State” (Now Kayin State), Myanmar and there is a ‘Cotype’ label under the specimen, it is still unknown if this specimen belongs to the syntypes described by the author.]

3♂1♀ (IZCAS), labels: 1) **Yunnan**, Xi-shuang-ban-na, Meng-a (勐阿), 1050–1080 m, Chinese Academy of Sciences [in Chinese]; 2) 1958.VI.2–10, leg. Shuyong Wang [in Chinese]; 3) *Phorocardiusflavus* Det. Shihong Jiang, 1999; 4) *Phorocardiusmanuleatus* (Candèze, 1888) Det. Ruan, 2018. • 1♂ (IZCAS), labels: 1) **Yunnan**, Xi-shuang-ban-na, Meng-zhe (勐遮), 1200 m, Chinese Academy of Sciences [in Chinese]; 2) 1958.IV.14, leg. Shuyong Wang [in Chinese]; 3) *Phorocardiusmanuleatus* (Candèze, 1888) Det. Ruan, 2018. • 1♂ (IZCAS), labels: 1) Da-nuo-you IV B 26.04.2009 leg. L.Z.Meng, gift from Na-ban-he Nature reserve [in Chinese]; 2) **Yunnan**, Jing-hong, Na-ban-he Nature reserve, Meng-song county, Da-nuo-you (大糯有), 2009.IV.26, 770 m, Chinese Academy of Sciences [in Chinese]; 3) 22.20699°N, 100.63761°E, Malaise trap, leg. Linzeng Meng, Chinese Academy of Sciences [in Chinese]; 4) *Phorocardiusmanuleatus* (Candèze, 1888) Det. Ruan, 2018. • 1♀ (SZPT), labels: 1) **Yunnan**, Xi-shuang-ban-na, Meng-hun (勐混), Chinese Academy of Sciences [in Chinese]; 2) 1958.V.31, leg. Chun-pei Hong [in Chinese]; 3) *Phorocardiusflavus* Det. Shihong Jiang, 1999; 4) *Phorocardiusmanuleatus* (Candèze, 1888) Det. Ruan, 2018. • 1♀ (IZCAS), labels: 1) **Yunnan**, Xi-shuang-ban-na, Meng-hun (勐混), Chinese Academy of Sciences [in Chinese]; 2) 1958.VI.12, leg. Yirang Zhang [in Chinese]; 3) *Phorocardiusmanuleatus* (Candèze, 1888) Det. Ruan, 2018. • 1♂ (IZCAS), labels: 1) **Yunnan**, Xi-shuang-ban-na, Da-meng-long (大勐龙), 650 m, Chinese Academy of Sciences [in Chinese]; 2) 1958.IV.18, leg. Fu-ji Pu [in Chinese]; 3) *Phorocardiusmanuleatus* (Candèze, 1888) Det. Ruan, 2018. • 1♂ (IZCAS), labels: 1) **Yunnan**, Xi-shuang-ban-na, Meng-la (勐腊), 620–650 m, Chinese Academy of Sciences [in Chinese]; 2) 1958.V.17, leg. Fa-cai Zhang [in Chinese]; 3) *Phorocardiusmanuleatus* (Candèze, 1888) Det. Ruan, 2018. • 4♂2♀ (IZCAS), labels: 1) **Yunnan**, Meng-la (勐腊), 670 m, Chinese Academy of Sciences [in Chinese]; 2) 1982.IV.20, leg. Subai Liao [in Chinese]; 3) *Phorocardiusmanuleatus* (Candèze, 1888) Det. Ruan, 2018. • 3♂1♀ (IZCAS), labels: 1) **Yunnan**, Gan-lang-ba (橄榄坝), 560 m, Chinese Academy of Sciences [in Chinese]; 2) 1957.IV.19, leg. Guangji Hong [in Chinese]; 3) *Phorocardiusflavus* Det. Shihong Jiang, 1999; 4) *Phorocardiusmanuleatus* (Candèze, 1888) Det. Ruan, 2018. • 1♂1♀ (IZCAS), labels: 1) **Yunnan**, Si-mao (思茅), 1200 m, 1957.V.11, leg. Shuyong Wang [in Chinese]; 2) leg. Guangji Hong [in Chinese]; 3) *Phorocardiusflavus* Det. Shihong Jiang, 1999; 4) *Phorocardiusmanuleatus* (Candèze, 1888) Det. Ruan, 2018. • 1♂ (IZCAS), labels: 1) **Yunnan**, close to Si-mao (思茅), 750 m, 1957.V.11, leg. Д. панфилов [Russian name, written in Chinese]; 2) *Phorocardiusmanuleatus* (Candèze, 1888) Det. Ruan, 2018.

###### Remarks.

This species is unusual for its extensively varied body color. The following three main patterns were found in examined specimens. Color pattern 1 (Fig. 10A, C): black on dorsum, with orange or yellow spot on base of each elytron; venter black before mesocoxae, orange from mesocoxae to abdominal ventrite IV, brown-black on abdominal ventrite V; antenna brown; and leg orange, yellow from coxa to mid-length of tibia in some, brown from mid-length of tibia to apex. Color pattern 2 (Fig. 11E): head black; pronotum with different combinations and proportions of colors, orange at sides in most, black near midline and posterior; elytron black, yellow or mixed with brown and yellow; venter mixed with orange and black; and leg orange or yellow from coxa to mid-length of tibia, brown from mid-length of tibia to apex. Color pattern 3: dorsum and venter entirely black to black-brown, antennae brown, and legs yellow-brown on basal half, brown on apical half.

The aedeagus slightly varied in the apex shapes of the median lobe and parameres. In rare cases, the sides of the pronotum are dramatically wider and more robust (e.g., Fig. 11E_1_). A comparison of specimens using mitochondrial DNA would be useful to test species boundaries.

Based on specimens from Yunnan, this species inhabits low to middle elevations (ca. 500–1200 m). Yunnan is mountainous, rainy, subtropical to tropical, with subtropical evergreen broad-leaf forest or tropical rain forest. Some of our specimens are collected from Xi-shuang-ban-na tropical rain forest. Known from Oriental Region only.

This species was treated as a subspecies of *Phorocardiusmelanopterus* (Candèze, 1878) by Fleutiaux (1931: 311). We have studied the photograph of the single type specimen of *P.melanopterus* [RBINS, label information: Coll. R. I. SC. N. B., CAMBODGE // Collection E. Candèze // n. sp. *Melanopterus* cdz., Cambodia // *Cardiophorusmelanopterus*, Cd., dèt. E. Candèze // Type]. In that specimen, the head is brown-black, the rest of the body is entirely brown throughout including legs and basal four antennomeres (all other antennomeres are missing on the type specimen), and the pronotum with lateral carina diverging from hind angle carina. Its color is different from all known color patterns of *P.manuleatus*. Additionally, *P.melanopterus* probably does not belong to *Phorocardius* because of the presence of pronotal lateral carina (see checklist above).

##### 6. Phorocardius minutus

Taxon classificationAnimaliaColeopteraElateridae

Ruan & Douglas sp. nov.

AD1819FF-55E2-5A3A-A257-69E82EE57BEE

http://zoobank.org/00316F6E-6213-4A86-A88D-8430EBC4165B

**Figure 12.**
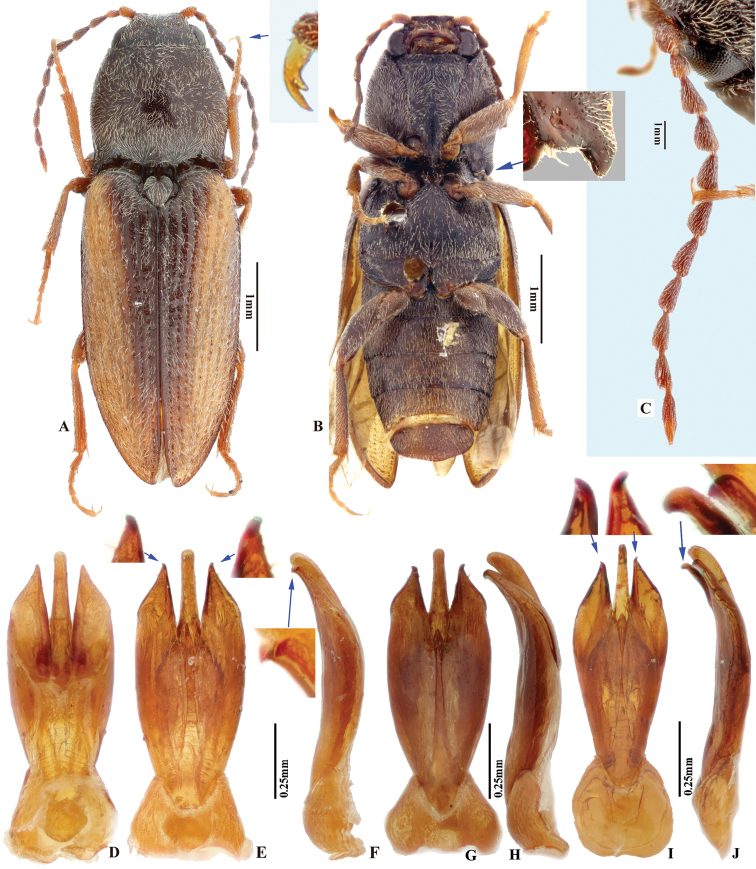
*Phorocardiusminutus* Ruan & Douglas, sp. nov. **A** holotype, habitus, dorsal view, arrow indicating claw **B** paratype, habitus, ventral view, arrow indicating hypomeral hind edges **C** antennae of holotype, showing triangular antennomeres III–IV **D–F** aedeagus of holotype **D** dorsal view **E** ventral view, arrows indicating apices of parameres **F** lateral view, arrow indicating apices of parameres **G, H** aedeagus of paratype collected from Da-qing-gou, Inner Mongolia, ventral and lateral views **I, J** aedeagus of paratype collected from Ke-you-zhong-qi, Inner Mongolia, ventral and lateral views, arrows indicating apices of parameres.

**Figure 13.**
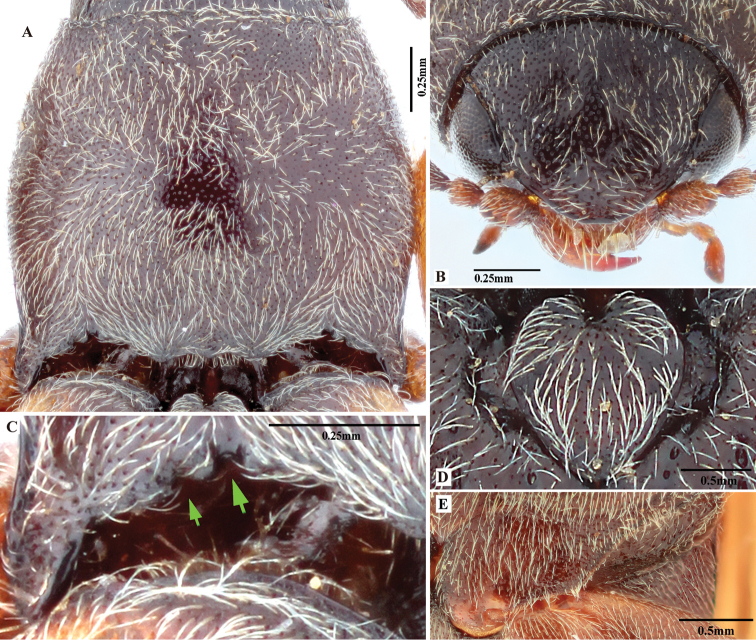
*Phorocardiusminutus* Ruan & Douglas, sp. nov. **A** pronotum of holotype, dorsal view **B** head of holotype, dorsal-frontal view **C** posterior edge of pronotum of holotype, left side, dorsal view, arrows indicating sublateral incisions **D** scutellar shield of holotype, dorsal view **E** metacoxal plate of paratype, ventral view.

Figs 12 , 13 , 23F , 24F , 25F

###### Type locality.

Inner Mongolia: Da-yin-zi, Linxi County (“Ta-Yngtse, Linsisien”).

###### Etymology.

This species is named for its small body size.

###### Distribution.

China (Inner Mongolia).

###### Differential diagnosis.

Body length 5–7 mm. Prothorax: procoxal cavities open; prosternal process gradually narrowed posterad to ventral apex in ventral view, with apex narrowly rounded. Pterothorax: scutellar shield with posterior apex narrowly rounded. Tarsal claw with ventral apex smaller than dorsal apex. Male genitalia: paramere with apex pointed and bent laterad and ventrad, without preapical lateral expansion or apical mesal callus. Female unknown.

*Phorocardiusminutus* Ruan & Douglas, sp. nov. is distinct for its small body size, color, and more robust appendages compared to other Chinese species. Its partly yellow elytra resemble *P.flavistriolatus* Ruan & Douglas, sp. nov., and *P.comptus* (Candèze, 1860).

This species can be differentiated from *P.flavistriolatus* Ruan & Douglas, sp. nov. by the following characters: in *P.minutus* Ruan & Douglas, sp. nov., aedeagus with paramere apex claw-like, produced laterally; males are less than 6.3 mm in body length; and body brown, elytra yellow, with suture and lateral-basal edges near epipleura brown; while in *P.flavistriolatus* Ruan & Douglas, sp. nov., aedeagus with apex of paramere not claw like or produced laterally; males are longer than 7 mm; body black-brown; and elytra black-brown with two longitudinal yellow stripes.

*P.minutus* Ruan & Douglas, sp. nov. can be differentiated from *P.comptus* by the following characters: in *P.minutus* Ruan & Douglas, sp. nov., males are less than 6.3 mm in body length; and body brown, elytra yellow, with suture and lateral-basal edges near epipleura brown; while in *P.comptus*, males are longer than 7 mm; and body black, elytra black with two longitudinal yellow stripes.

###### Description.

(Based on all type specimens) Dorsum matt. Head brown, with mouthparts red-brown to pale brown. Antennae pale brown. Pronotum brown, with posterior edge dark brown. Scutellar shield brown. Elytra yellow, with suture and lateral-basal edges near epipleura brown. Ventral surfaces brown, including hypomera. Epipleura brown. Legs pale brown to brown. Body with yellow pubescence.

###### Measurements.

(based on all type specimens) Male body length 5.2–6.3 mm, width 1.6–2.2 mm. Body length to width ratio 2.9–3.2. Pronotal width to length ratio 1.1–1.2. Pronotum slightly narrower than elytra, Pronotal width to body width ratio 0.86–0.87. Elytral length to pronotal length ratio 2.7–2.9; elytron length to width ratio 3.9–4.3.

***Head*.** Frons and vertex with interspaces between punctures 1–3 × average puncture diameter. In frontal view, edge of frontal carina convex. Antenna with apex extending slightly over posterior angle of pronotum. Distance between eyes to width of eye ratio 4.1–4.5. Antenna length to body length ratio 0.40–0.45; proportions of antennomere length as follows: 100 (scape); 66–70; 98–100; 88–95; 97–107; 97–110; 108–112; 110–115; 109–120; 104–110; 139–142.

***Prothorax*.** Pronotum in dorsal view (Fig. 13A): sides convex from anterior to near posterior fourth, concave on posterior fourth, widest near mid-length; posterior angles with lateral sides slightly and evenly convex; surface with interspaces between punctures 1–2 × average puncture diameter. In ventral view, ventral surface of prosternal process with sides carinate at basal half (not carinate at apical half), gradually narrow from anterior to posterior end, with apex narrowly rounded. In lateral view, prosternal process with ventral surface curved strongly dorsad, posterior end weakly concave (Fig. 24F, upper arrow). Procoxal cavities open.

***Pterothorax*** (Figs 24F, 25F). Mesepisternum in ventral view with antero-mesal angle broadly rounded (Fig. 25F, upper (green) arrow). Projections on posterior edge of mesosternum absent in ventral view (Fig. 25F, lower (red) arrow) and lateral view (Fig. 24F, lower (red) arrow). Scutellar shield: short, width to length ratio 0.85–0.94; anterolateral edges evenly convex; posterior apex narrowly rounded. Elytra: upper edge of epipleura with minute serrations.

***Legs*.** Femora and tibiae thick. Length ratio of metatarsomeres I–V (excluding claws): 100; 66–76; 60–63; 45–48; 110–120. Claw with ventral apex much smaller than dorsal apex.

***Abdomen*.** Lateral edges of visible abdominal ventrites I–V with minute serrations.

***Male genitalia*.** Slender in ventral and lateral views. Median lobe in ventral view gradually narrowed from base to rounded apex. Median lobe in lateral view gently curved ventrad, apex rounded. Paramere in ventral view: wide, width 2–2.5 × median lobe width (measured across the mid-length of paramere and median lobe respectively), widest near apical third, abruptly narrowed near apex; apex pointed, bent laterally; preapical lateral expansion and apical mesal callus absent. Paramere in lateral view: robust, almost straight from base to mid-length; bent ventrad from mid-length to apex; apex pointed and facing ventrad, claw-like, but not hooked.

**Female.** Unknown.

###### Type material.

***Holotype***. ♂ (IZCAS), labels: 1) Manchoukuo, Ta-Yngtse (大营子), Linsisien (林西县), leg. E. Bourgault, VII.1940; 2) *Phorocardiuscomptus* Cand. Det. Siqin Ge; 3) Holotype *Phorocardiusminutus* sp. nov. Des. Ruan et al., 2019.

***Paratypes*** (3♂). 1♂ (IZCAS), labels: 1) Inner Mongolia, Ke-you-zhong-qi (科右中旗), stock farm, 1995.VII.15 [in Chinese]; 2) leg. Mingzhi Yang [in Chinese]; 3) Cardiopnorine; 4) Elateridae; 5) Paratype *Phorocardiusminutus* sp. nov. Des. Ruan et al., 2019. • 1♂ (SZPT), labels: 1) Inner Mongolia, Da-qing-gou (大青沟), Xiao-qing-hu, 19.VII.2013, leg. Kai Shi [in Chinese]; 2) Paratype *Phorocardiusminutus* sp. nov. Des. Ruan et al., 2019. • 1♂ (SZPT), labels: 1) Inner Mongolia, Da-qing-gou (大青沟), Xiao-qing-hu, sweeping, 20.VII.2013, leg. Kai Shi [in Chinese]; 2) Paratype *Phorocardiusminutus* sp. nov. Des. Ruan et al., 2019.

###### Remarks.

The aedeagus of one paratype collected from ‘Ke-you-zhong-qi, Inner mongolia’ is slightly different from that of the holotype by being slender in ventral and lateral views (Fig. 11I, J). However, all external characters of this individual (e.g., body color, shape and length, punctures on head and pronotum) are identical with the Holotype. The slight differences in the shape of aedeagus are treated as intraspecific variation here.

Previously, the genus *Phorocardius* was only known from the Oriental Region. The discovery of *P.minutus* Ruan & Douglas, sp. nov. from the Palearctic Region indicates that the members of this genus can survive in areas with freezing winter temperatures (in Ke-you-zhong-qi, Inner Mongolia, the minimum temperature is approximately –20 °C in January). Examination of female genitalia or phylogenetic studies would be useful to see how closely this species is related to other *Phorocardius*. The thick tibiae and ascendant prosternal process of this species are similar to many fossorial elaterids (Douglas 2011), including many species with flightless females. Females of this species are currently unknown.

*Phorocardiusminutus* Ruan & Douglas, sp. nov. is like *Diocarphussolitarius* (Fleutiaux, 1931) in the ventral apex of the tarsal claw is much smaller than the dorsal apex. However, *P.minutus* can be easily separated from *Diocarphus* by its open procoxal cavities and the pronotum without pronotal lateral carina.

Based on specimen information, this species inhabits low elevation areas (ca. 0–500 m) in Inner Mongolia, north China. This area is arid with temperate grassland and shrubland and cold winters. Known only from the Palearctic Region.

##### 7. Phorocardius rufiposterus

Taxon classificationAnimaliaColeopteraElateridae

Ruan & Douglas sp. nov.

BD0BE45C-0328-5BDC-B8B0-B37D1678FA6F

http://zoobank.org/60CF4626-B266-496A-8B46-344DB7BCF2E6

**Figure 14.**
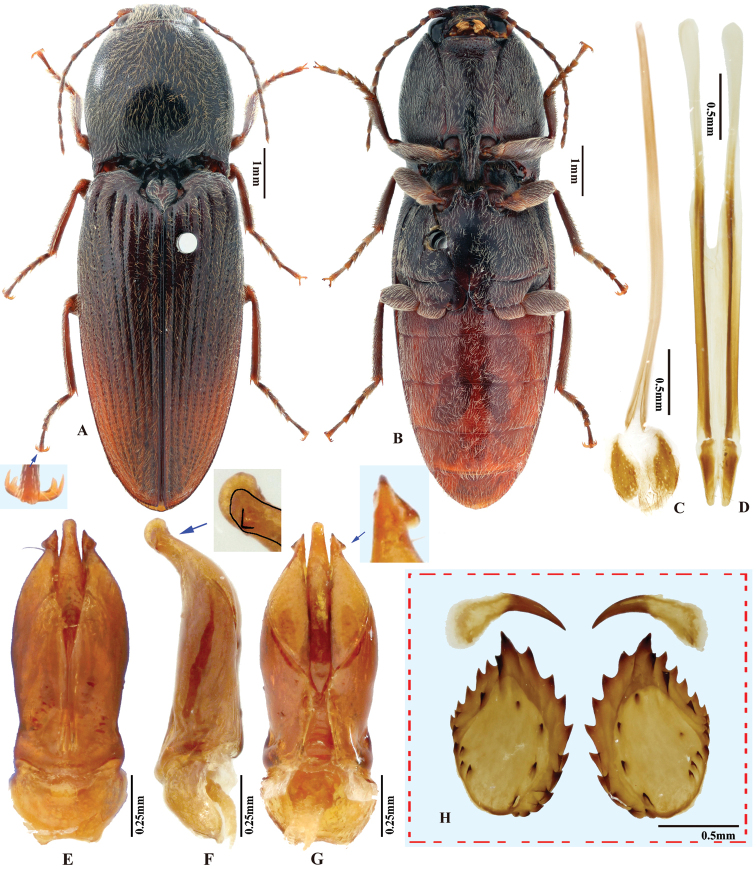
*Phorocardiusrufiposterus* Ruan & Douglas, sp. nov. **A** holotype, habitus, dorsal view, arrow indicating claws **B** holotype, habitus, ventral view **C** female abdominal sternite VIII, dorsal view (paratype) **D** ovipositor, dorsal view (paratype) **E** aedeagus, ventral view (paratype) **F** aedeagus, lateral view (paratype), arrow indicating apex of paramere and median lobe **G** aedeagus, dorsal view (paratype), arrow indicating apex of paramere **H** distal (upper side) and proximal sclerites of bursa copulatrix.

**Figure 15.**
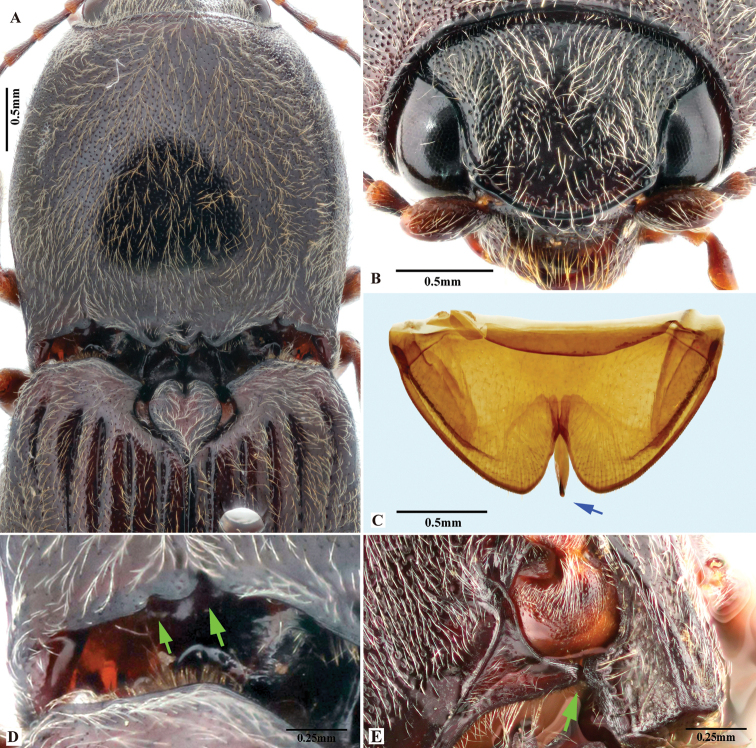
*Phorocardiusrufiposterus* Ruan & Douglas, sp. nov. **A** pronotum and scutellar shield of holotype, dorsal view **B** head, frontal view **C** last abdominal ventrite (ventrite V) of female, dorsal view, arrow indicating blade-like projection **D** posterior edge of pronotum, left side, dorsal view, arrows indicating sublateral incisions **E** procoxa, lateral-ventral view, arrow indicating closed procoxal cavity.

Figs 14 , 15 , 23G , 24G , 25G , 26F

###### Type locality.

Yunnan, Xi-shuang-ban-na, Xiao-meng-yang.

###### Etymology.

This species is named after the red-brown color of the posterior half of the body.

###### Distribution.

China (Yunnan).

###### Differential diagnosis.

Body length greater than 7.0 mm; integument black (non-metallic) anteriorly, fading to red-brown or yellow-brown on posterior half. Prothorax: procoxal cavities closed (narrowly open in a few); prosternal process not strongly narrowed posterad from base to ventral apex in ventral view, ventral apex almost truncate. Male genitalia: paramere acute beyond preapical lateral expansion; with preapical lateral expansion present, without apical mesal callus. Female: apex of last abdominal ventrite (ventrite V) with longitudinal slender blade-shaped projection at middle, deeply emarginate at sides.

This species is unique for its closed procoxal cavities.

It resembles *Phorocardiusmagnus* Fleutiaux, 1931 in the general body shape and the lighter color on the posterior half of body. They can be distinguished by the following characters: in *P.rufiposterus* Ruan & Douglas, sp. nov., aedeagus with apex of paramere robust (sides convex in dorsal view before large preapical expansion), apex spear-shaped in ventral view with acute tip and triangular preapical lateral expansion; head with frontal carina convex in frontal view; and female with ventrite V deeply emarginate, a slender blade-shaped projection present at middle (Fig. 15C), while in *P.magnus*, body entirely red-brown to brown throughout, with pronotum slightly darker; aedeagus with paramere narrow and concave before small preapical expansion in ventral view; head with frontal carina straight in frontal view; and female with the apex of abdominal ventrite V tri-lobed, middle lobe semicircular to longitudinal with apex rounded, not slender blade-shaped.

###### Description.

(based on all type specimens) Body black anteriorly, fading to red-brown or yellow-brown on posterior half. Dorsum glabrous and shiny. Head black, with mouthparts red-brown to dark brown. Antennae brown. Pronotum black, with anterior and posterior edge brown. Elytra black to dark red-brown anteriorly, fading to red-brown or yellow-brown posteriorly. Venter black to dark red-brown on anterior half, fading to red-brown or yellow-brown on posterior half. Epipleura red-brown. Legs red-brown to dark red-brown. Body surface covered with yellow-grey pubescence.

###### Measurements.

(based on all type specimens) Male body length 8.2–10.2 mm, width 2.6–3.2 mm. Female body length 8.5–10.6 mm, width 3.0–3.5 mm. Body length to width ratio 3.0–3.2. Pronotal width to length ratio 1.0–1.1. Pronotal width to body width ratio 0.87–0.90. Elytral length to pronotal length ratio 2.3–2.4; elytron length to width ratio 4.1–4.2.

***Head*.** Frons and vertex with interspaces between punctures 1.5–4 × average puncture diameter, sparsest at centre of frons. Frontal carina in frontal view convex, not straight. Antenna with apex not reaching beyond posterior angle of pronotum. Distance between eyes to width of eye ratio in frontal view 3.0–3.2. Antenna length to body length ratio, in male 0.36–0.37, in female 0.33–0.34; proportions of antennomere length (male) as follows: 100 (scape); 51–59; 75–80; 71–76; 75–80; 73–80; 68–78; 67–75; 73–80; 78–80; 92–99.

***Prothorax*.** Pronotum in dorsal view (Fig. 14A): sides evenly convex from apex to slight concavity near posterior third, widest near mid-length; posterior angles with lateral sides almost straight, not bulged; surface with interspaces between punctures 1.5–2.5 × average puncture diameter. Punctures much smaller or nearly absent posterad than at centre of disc. In ventral view, ventral surface of prosternal process with sides carinate and slightly and gradually narrow from anterior to mid-length, parallel-sided from mid-length to posterior end, apex almost truncate. In lateral view, prosternal process with ventral surface curved slightly dorsad, posterior end with ventral 2/3 almost straight, dorsal 1/3 produced posteriorly (Fig. 24G, upper arrow). Procoxal cavity closed (Fig. 15E), narrowly open in a few.

***Pterothorax*** (Figs 24G, 25G). Mesepisternum in ventral view with antero-mesal angle acute, long (Fig. 25G, upper (green) arrow). Projections on posterior edge of mesosternum: in ventral view weakly developed (Fig. 25G, lower (red) arrow); in lateral view almost absent (Fig. 24G, lower (red) arrow). Scutellar shield: width to length ratio 0.88–0.90; anterolateral edges slightly sinuate; posterior apex pointed. Elytra: upper edge of epipleura with minute serrations.

***Legs*.** Length ratio of metatarsomeres I–V (excluding claws): 100; 80–90; 65–75; 57–62; 137–155. Claw with ventral apex almost as large as dorsal apex.

***Abdomen*.** Lateral edges of visible abdominal ventrites I–V with minute serrations.

***Male genitalia*.** Robust in ventral and lateral views. Median lobe in ventral view gradually narrowed from base to near apex, apex rounded to apically flattened. Median lobe in lateral view curved ventrad, with apex dilated and recurved dorsad. Paramere in ventral view: wide, width 2.5–3.5 × median lobe width (measured at mid-length of paramere and median lobe respectively); widest near mid-length, gradually narrowed and with outer sides evenly convex towards apex; apex spear-shaped, with acute tip and triangular preapical lateral expansion, apical mesal callus absent. Paramere in lateral view: robust, almost straight from base to apical third, gradually narrowed and bent ventrad from apical third to apex; apex slightly recurved dorsad, preapical ventral expansion absent, with an angulate structure near apex (see Fig. 14F), without hook-shaped structure.

**Female.** Body color like male. Ventrite V deeply emarginate at apex, with longitudinal slender blade-shaped projection at midline (Fig. 15C) (male with ventrite V entirely convex and rounded). Proximal sclerites of bursa copulatrix oval, apex acute (Fig. 14H): base with without concavity, each with 14–16 large spines occupying two-thirds of edges, 9–10 smaller spines on disc.

###### Type material.

***Holotype***. ♂ (IZCAS), labels: 1) **Yunnan**, Xi-shuang-ban-na, Xiao-meng-yang (小勐养), 850 m, Chinese Academy of Sciences [in Chinese]; 2) 1957.VI.18, leg. Linchao Zang [in Chinese]; 3) Holotype *Phorocardiusrufiposterus* sp. nov. Des. Ruan et al., 2019.

***Paratypes*** (23♂, 11♀). 1♂ (SZPT), labels: 1) **Yunnan**, Xi-shuang-ban-na, Xiao-meng-yang (小勐养), 850 m, Chinese Academy of Sciences [in Chinese]; 2) 1957.IX.13, leg. Shuyong Wang [in Chinese]; 3) *Phorocardius* sp. det. Shihong Jiang, 1999; 4) Paratype *Phorocardiusrufiposterus* sp. nov. Des. Ruan et al., 2019. • 2♀ (SZPT), labels: 1) **Yunnan**, Lin-cang, Yun-xian County, Man-wang township (漫湾镇), light trap, VI–VII, leg. Zichun Xiong, Shenzhen Polytechnic [in Chinese]; 2) Paratype *Phorocardiusrufiposterus* sp. nov. Des. Ruan et al., 2019. • 2♂ (SZPT), labels: 1) CN: **Yunnan**, Xinping County (新平), 2013.VI.7, Collector unknown, Shenzhen Polytechnic [partly in Chinese]; 2) Paratype *Phorocardiusrufiposterus* sp. nov. Des. Ruan et al., 2019. • 1♂ (SZPT), labels: 1) **Yunnan**, Xi-shuang-ban-na, Meng-a (勐阿), 1000 m, Chinese Academy of Sciences [in Chinese]; 2) 1958.VI.21, leg. Shuyong Wang [in Chinese]; 3) *Phorocardius* sp. det. Shihong Jiang, 1999; 4) Paratype *Phorocardiusrufiposterus* sp. nov. Des. Ruan et al., 2019. • 2♂2♀ (IZCAS), labels: 1) **Yunnan**, Xi-shuang-ban-na, Xiao-meng-yang (小勐养), 850 m, Chinese Academy of Sciences [in Chinese]; 2) 1957.VI.14–20, leg. Shuyong Wang & Linchao Zang [in Chinese]; 3) Paratype *Phorocardiusrufiposterus* sp. nov. Des. Ruan et al., 2019. • 2♂ (IZCAS), labels: 1) Shuan Jiang (双江县), 55-VI; 2) Paratype *Phorocardiusrufiposterus* sp. nov. Des. Ruan et al., 2019. • 1♂ (IZCAS), labels: 1) **Yunnan**, Xi-shuang-ban-na, Meng-la (勐腊), 620–650 m, Chinese Academy of Sciences [in Chinese]; 2) 1958.VI.10, leg. Yirang Zhang [in Chinese]; 3) Paratype *Phorocardiusrufiposterus* sp. nov. Des. Ruan et al., 2019. • 3♂4♀ (IZCAS), labels: 1) **Yunnan**, Xi-shuang-ban-na, Meng-a (勐阿), 800–1080 m, Chinese Academy of Sciences [in Chinese]; 2) 1958.V–VIII, leg. Shuyong Wang & Fuji Pu [in Chinese]; 4) Paratype *Phorocardiusrufiposterus* sp. nov. Des. Ruan et al., 2019. • 4♂2♀ (IZCAS), labels: 1) **Yunnan**, Xi-shuang-ban-na, Meng-hun (勐混), 650–950 m, Chinese Academy of Sciences [in Chinese]; 2) 1958.VI.3–15, leg. Xuwu Meng, Shuyong Wang & Chunpei Hong [in Chinese]; 3) Paratype *Phorocardiusrufiposterus* sp. nov. Des. Ruan et al., 2019. • 4♂ (IZCAS), labels: 1) **Yunnan**, Xi-shuang-ban-na, Meng-hun (勐混), 650–1200 m, Chinese Academy of Sciences [in Chinese]; 2) 1958.V–VII, leg. Xuwu Meng & Zhixing Chen [in Chinese]; 3) Paratype *Phorocardiusrufiposterus* sp. nov. Des. Ruan et al., 2019. • 1♂ (TARI , ex. SZPT), labels: 1) **Yunnan**, Xi-shuang-ban-na, Meng-hun (勐混), 750 m, Chinese Academy of Sciences [in Chinese]; 2) 1958.V-31, leg. Xuwu Meng [in Chinese]; 3) Paratype *Phorocardiusrufiposterus* sp. nov. Des. Ruan, 2018. • 1♀ (IZCAS), labels: 1) **Yunnan**, Jing-dong (景东), 1170 m, 1958.VII.3 [in Chinese]; 2) Paratype *Phorocardiusrufiposterus* sp. nov. Des. Ruan et al., 2019. • 1♂ (IZCAS), labels: 1) southwest **Yunnan**, close to Jing-ping (金平), 1170 m, 1956.VII.27, leg. панфилов [written in Russian]; 2) Paratype *Phorocardiusrufiposterus* sp. nov. Des. Ruan et al., 2019. • 1♂ (IZCAS), labels: 1) **Yunnan**, Lan-cang (澜沧), 1000 m, 1957.VII.29, leg. Lingchao Zang [in Chinese]; 2) Paratype *Phorocardiusrufiposterus* sp. nov. Des. Ruan et al., 2019.

###### Remarks.

This species is unique for its closed procoxal cavities, which has not been reported in other *Phorocardius* species. However, other aspects of this species are consistent with generic traits of *Phorocardius*. These are: characteristic claws and female and male genitalia.

Integument color varies slightly between individuals. However, the gradual change of color from anterior to posterior end of the body can be observed in all specimens.

Based on specimen information, this species inhabits low to middle elevations (ca. 500–1500 m) in Yunnan Prov., south China. Yunnan is rainy, subtropical to tropical, with evergreen broad-leaf forest or tropical rain forest. This species is distributed only in the Oriental Region.

##### 8. Phorocardius unguicularis

Taxon classificationAnimaliaColeopteraElateridae

(Fleutiaux, 1918)

531C46BD-6AAE-5077-A4AD-FA5CDB02AA38

**Figure 16.**
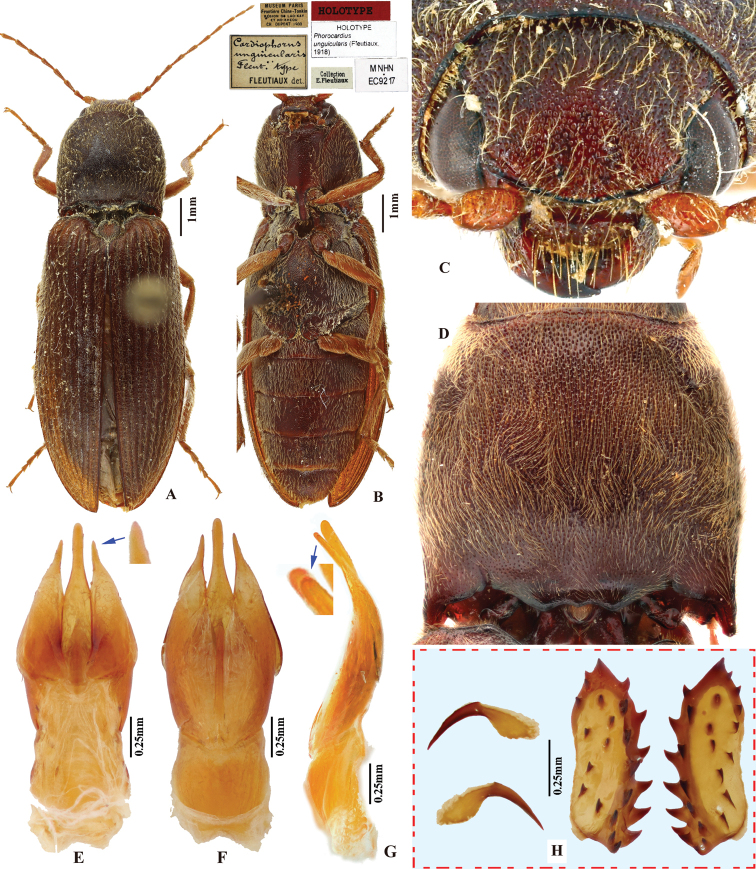
*Phorocardiusunguicularis* (Fleutiaux, 1918). **A** holotype habitus, dorsal view (photograph: Dr Antoine Mantilleri, MNHN) **B** holotype habitus, ventral view with specimen labels (photograph: Dr Antoine Mantilleri, MNHN) **C** head of holotype, frontal view **D** pronotum (non-type specimen) **E** aedeagus, dorsal view (non-type specimen), arrow indicating apex of paramere **F** aedeagus, ventral view (non-type specimen) **G** aedeagus, lateral view (non-type specimen), arrow indicating apices of parameres **H** distal (left side) and proximal sclerites of bursa copulatrix (inner view, non-type specimen).

Figs 16 , 23H , 24H , 25H , 26G

Cardiophorus unguicularis Fleutiaux, 1918: 222. Type locality: “Tonkin: Région de Lao-Kay et de Ho-Khéou, frontière de Chine”, interpreted as Vietnam: the area near frontier of Lao-Cai city (Vietnam) and He-Kou city (China).
Phorocardius unguicularis : Fleutiaux 1913: 311.

###### Distribution.

China: Yunnan (Fleutiaux 1931, 1947), Hainan (new record), Sichuan (“Se-Tchouen, Aubert” – Fleutiaux (1931)); Vietnam (Fleutiaux 1918).

###### Differential diagnosis.

Body length greater than 7.0 mm; integument brown to dark brown. Prothorax: procoxal cavities open; prosternal process gradually and only slightly narrowed posterad to ventral apex in ventral view, with apex almost truncate. Pterothorax: scutellar shield with posterior apex pointed. Tarsal claw with ventral apex not smaller than dorsal apex. Male genitalia: paramere without preapical lateral expansion or apical mesal callus in any view. Female: apex of last abdominal ventrite (ventrite V) simple, not emarginate at apex.

This species is unique among Chinese *Phorocardius* species by having extremely dense pronotal punctation (interspaces between pronotal punctures 0.3–1 × average puncture diameter).

*Phorocardiusunguicularis* (Fleutiaux, 1918) resembles *P.magnus* Fleutiaux, 1931 in body color and size. They can be separated by the following combination of characters: in *P.unguicularis* (Fleutiaux, 1918), aedeagus slender in lateral view (paramere maximum thickness 1/5 paramere length), in ventral view paramere widened from base to mid-length, narrowed from mid-length to apex, apex pointed and without preapical lateral expansion; pronotum with deep punctures, interspaces between punctures 0.3–1 × average puncture diameter; and head with frontal carina convex at middle in frontal view; while in *P.magnus* Fleutiaux, 1931, aedeagus robust in lateral view (paramere maximum thickness 1/3 paramere length); in ventral view, paramere slightly widened from base to apical fourth, apical fourth abruptly narrowed, with hook-like preapical lateral expansion; pronotum with interspaces between punctures 1–2.5 × average puncture diameter; and head with frontal carina straight in frontal view.

*Phorocardiusunguicularis* (Fleutiaux, 1918) resembles *P.yanagiharae* (Miwa, 1927) in body color. They can be separated by the following combination of characters. In *P.unguicularis* (Fleutiaux, 1918), in ventral view, aedeagus with paramere gradually narrowed from mid-length to apex, apex pointed and without preapical lateral expansion; pronotum with deep punctures, interspaces between punctures 0.3–1 × average puncture diameter; and head with frontal carina convex in frontal view; while in *P.yanagiharae* (Miwa, 1927), in ventral view, aedeagus with paramere, abruptly narrowed from apical third to apex, apex with hook-like preapical lateral expansion; pronotum with shallow punctures, interspaces between punctures 1–2 × average puncture diameter; and head with frontal carina straight in frontal view.

*Phorocardiusunguicularis* is also similar to *P.astutus* (Candèze, 1888), it differs from the latter based on the following characters: body brown to dark-brown with yellow-brown appendages; legs yellow-brown throughout; proximal sclerites of copulatrix kidney-shaped with slight basal concavity; and parameres of aedeagus without pre-apical lateral expansions. In *P.astutus*: body brown-black with dark appendages; legs with red-brown joints; proximal sclerites of copulatrix oval, not kidney-shaped, without basal concavity; and parameres wedge-like with pre-apical lateral expansions. Additionally, *P.unguicularis* has narrower pronotum and body, and larger body length. Further study of these two species would be important to verify their status.

###### Description.

(Based on photographs of the holotype and all examined specimens) Body brown to dark brown (brown-black in a few); legs and antennae brown; pronotum slightly darker than rest (Fig. 16A, B). Integument matt, with yellow pubescence.

###### Measurements.

(based on the type and non-type specimens) Male body length 8.6–11.2 mm, width 2.2–3.2 mm. Female body length 9.6–12.8 mm, width 3.0–4.0 mm. Body length to width ratio 2.9–3.1. Pronotal width to length ratio 1.1–1.2. Pronotal width to body width ratio 0.76–0.81. Elytral length to pronotal length ratio 2.7–3.1; elytron length to width ratio 4.4–4.7.

***Head*.** Frons and vertex with interspaces between punctures 0.5–1.5 × average puncture diameter. Frontal carina in frontal view convex, not straight. Antenna with apex slightly extending over base of elytron, slightly varied long in different individuals. Distance between eyes to width of eye ratio 3.6–3.8. Antenna length to body length ratio, in male 0.39–0.42, in female 0.37–0.39. Proportions of antennomere lengths (male): 100 (scape); 60–65; 80–85; 95–99; 95–99; 91–95; 94–104; 92–95; 86–90; 73–83; 100–105.

***Prothorax*.** Pronotum in dorsal view: sides evenly convex, widest near mid-length (Fig. 16C); posterior angles with lateral sides almost straight, slightly convex and bulged at basal half in some cases; surface with interspaces between punctures 0.3–1 × average puncture diameter. In ventral view, ventral surface of prosternal process with sides not carinate and gradually narrowed from anterior to near posterior end, apex almost truncate. In lateral view, prosternal process with ventral surface curved slightly dorsad, posterior end with ventral 2/3 almost straight, dorsal 1/3 produced posteriorly (Fig. 24H, upper arrow). Procoxal cavities open.

***Pterothorax*** (Figs 24H, 25H). Mesepisternum in ventral view with antero-mesal angle right-angled (Fig. 25H, upper (green) arrow). Projections on posterior edge of mesosternum: almost absent in ventral view (Fig. 25H, lower (red) arrow), and lateral view (Fig. 24H, lower (red) arrow). Scutellar shield: width to length ratio 0.94–0.95; anterolateral edges slightly sinuate; posterior apex pointed. Elytra: upper edge of epipleura with minute serrations.

***Legs*.** Length ratio of metatarsomeres I–V (excluding claws): 100; 78–85; 70–78; 50–57; 125–134. Claw with ventral apex almost as large as dorsal apex.

***Abdomen*.** Lateral edges of visible abdominal ventrites I–V with minute serrations.

***Male genitalia*.** Robust from ventral and dorsal views, slender in lateral view. Median lobe in ventral view gradually narrowing from base to near mid-length, apical half elongate with sides parallel-sided to slightly convex, apex narrowly rounded. Median lobe in lateral view almost straight, apex narrowly rounded. Paramere in ventral view: robust, width 3–4 × median lobe width (measured at mid-length of paramere and median lobe respectively), widest near mid-length; gradually narrowing from mid-length to apex, apex elongate and needle-like, without preapical lateral expansion or apical mesal callus (Fig. 16E). Paramere in lateral view: slender, gradually narrowing and curved ventrad from base to near apex; preapical ventral expansion absent, without hook-shaped structure.

**Female.** Body color like male. Apex of abdominal ventrite V slightly sinuate (Fig. 26G). Proximal sclerites of bursa copulatrix elongate-kidney shaped, apex acute (Fig. 16H), base with slight concavity: each with 13–15 large spines mainly on the convex mesal edge, 10–12 smaller spines on disc.

###### Type material.

***Holotype*** (sex unknown, in MNHN, photographs of holotype provided by Dr Antoine Mantilleri), labels: 1) Museum Paris, Frontière Chine-Tonkin, Region De Lao-Kay, Et Ho-Kheou, Ch. Dupont; 2) *Cardiophorusunguicularis* Fleut., type, Fleutiaux det.; 3) Collection E. Fleutiaux; 4) Holotype [red label]; 5) Holotype, *Phorocardiusunguicularis* (Fleutiaux, 1918); 6) MNHN EC9217.

###### Additional material.

1♀ (TARI ), without information of locality, with only one label: *Phorocardiusunguicularis* Fleut., Coll. E. Fleutiaux. 1♂16♀ (IZCAS), labels: 1) **Yunnan**, Li-jiang, Yu-long-shan (玉龙山), 2700 m, Chinese Academy of Sciences [in Chinese]; 2) 1984.VII.27, leg. Shuyong Wang [in Chinese]; 3) *Phorocardiusunguicularis* (Fleutiaux, 1918) Det. Ruan, 2018. • 4♂4♀ (IZCAS), labels: 1) **Yunnan**, Li-jiang, Yu-hu (玉湖), 2750 m, Chinese Academy of Sciences [in Chinese]; 2) 1984.VII.21–23, leg. Jiangguo Fang / Changfang Li [in Chinese]; 3) *Phorocardiusunguicularis* (Fleutiaux, 1918) Det. Ruan, 2018. • 1♀ (IZCAS), labels: 1) *Cardiophorus*, **Yunnan**, Collection Fleutiaux, Li-jiang, Yu-long-shan (玉龙山), 2700 m, Chinese Academy of Sciences [in Chinese]; 2) 1984.VII.27, leg. Shuyong Wang [in Chinese]; 3) *Phorocardiusunguicularis* (Fleutiaux, 1918) Det. Ruan, 2018. • 2♂7♀ (IZCAS), labels: 1) **Yunnan**, Yong-sheng, Liu-de (六德), 2250–2750 m, Chinese Academy of Sciences [in Chinese]; 2) 1984.VII, leg. Shuyong Wang et al. [in Chinese]; 3) *Phorocardiusunguicularis* (Fleutiaux, 1918) Det. Ruan, 2018. • 1♂ (IZCAS), labels: 1) **Hainan** Prov., Chang-jiang County, Ba-wang-ling, 145 m, 2007.V.7N, Chinese Academy of Sciences [in Chinese]; 2) 19.1104N 109.08168E, leg. Hongbin Liang, Chinese Academy of Sciences [in Chinese]; 3) *Phorocardiusunguicularis* (Fleutiaux, 1918) Det. Ruan, 2018.

###### Remarks.

Based on specimen information, this species inhabits low to high elevations in south China and north Vietnam. The highest elevation record for this species is around 2750 m. Some specimens were collected at the foot of Yu-long-shan Mountain (also known as Yu-long Snow Mountain), whose main peak is 5596 m, and with snowfall all the year-round above 3500 m. South China and north Vietnam are rainy, with subtropical to tropical climates, with subtropical evergreen broad-leaf forest or tropical rain forest. This species is known only from the Oriental Region.

##### 9. Phorocardius yanagiharae

Taxon classificationAnimaliaColeopteraElateridae

(Miwa, 1927)

9F674A35-716A-58F7-B08D-9D5C95C70EF4

Figs 17 , 18

Cardiophorus yanagiharae Miwa, 1927: 109. Type locality: Taiwan, Tainan (China).
Phorocardius yanagiharae : Miwa 1934: 209.

###### Distribution.

China: Taiwan (Miwa 1927, 1931).

###### Differential diagnosis.

Body length greater than 7.0 mm; integument red-brown to brown throughout. Prothorax: procoxal cavities open. Pterothorax: scutellar shield with posterior edge pointed. Tarsal claw with ventral apex not smaller than dorsal apex. Male genitalia: paramere with preapical lateral expansion present, without apical mesal callus. Female unknown.

*Phorocardiusyanagiharae* (Miwa, 1927) resembles *P.magnus* Fleutiaux, 1931 in general body color and shape. They can be separated by the following combination of characters. In *P.yanagiharae* (Miwa, 1927), in lateral view, aedeagus with median lobe straight at apex, with paramere slender and ca. 1/2 as wide as median lobe (measured near middle part); pronotum narrower than in *P.magnus*, pronotal width to body width ratio 0.86; and in dorsal view, pronotum with sides of posterior angles strongly bulged and convex (Fig. 18C, D); while in *P.magnus* Fleutiaux, 1931: in lateral view, aedeagus with median lobe recurved dorsally at apex, with paramere robust and as wide as median lobe (measured near middle part); pronotum wider than in *P.yanagiharae*, pronotal width to body width ratio 0.90–0.97; and in dorsal view, pronotum with sides of posterior angles not bulged, straight to slightly convex (Fig. 9A).

*Phorocardiusyanagiharae* (Miwa, 1927) also resembles *P.unguicularis* (Fleutiaux, 1918) in body color. They can be separated by the following combination of characters. In *P.yanagiharae* (Miwa, 1927), in ventral view, aedeagus with paramere, abruptly narrowed from apical third to apex, apex with hook-like preapical lateral expansion; pronotum with shallow punctures, interspaces between punctures 1–2 × average puncture diameter; and head with frontal carina straight in frontal view; while in *P.unguicularis* (Fleutiaux, 1918), in ventral view, aedeagus with paramere gradually narrowed from mid-length to apex, apex pointed and without preapical lateral expansion; pronotum with deep punctures, interspaces between punctures 0.3–1 × average puncture diameter; and head with frontal carina convex in frontal view.

###### Description.

(based on holotype) Color entirely red-brown to brown throughout, with legs and antennae yellow-brown to brown; pronotum and venter slightly darker than elytra. Integument matt, with light yellow pubescence.

###### Measurements.

(based on holotype) Body length 9.4 mm. Body width 3.3 mm. Body length to width ratio 2.9. Pronotal width to length ratio 1.1. Pronotal width to body width ratio 0.86. Elytral length to pronotal length ratio 2.6; elytron length to width ratio 4.3.

***Head*.** Frons and vertex with interspaces between punctures 1–3 × average puncture diameter. Frontal carina in frontal view transversely straight. Antenna with apex extending to posterior angle of pronotum. Distance between eyes to width of eye ratio 3.0. Antenna length to body length ratio 0.36.

***Prothorax*.** Pronotum in dorsal view (Fig. 18A): sides strongly convex from anterior edge to posterior fourth, slightly convex from posterior fourth to base of posterior angle, concave at base of posterior angle; widest near posterior third; posterior angles with lateral margin convex, strongly bulged laterally (Fig. 18C, D); surface with interspaces between punctures 1–2 × average puncture diameter.

**Figure 17.**
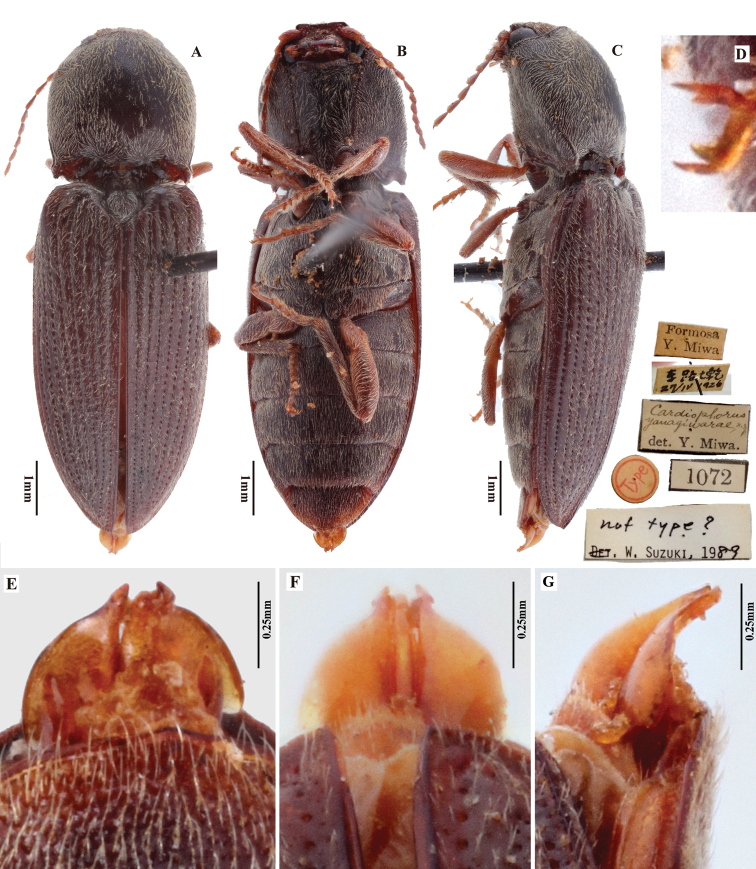
*Phorocardiusyanagiharae* (Miwa, 1927). **A** holotype, habitus, dorsal view **B** holotype, habitus, ventral view **C** holotype, habitus, lateral view with specimen labels **D** claw of holotype **E** apical part of aedeagus of holotype, ventral view **F** apical part of aedeagus of holotype, dorsal view **G** apical part of aedeagus of holotype, lateral view.

***Pterothorax*.** Projections on posterior edge of mesosternum: in lateral view present, acute, (Fig. 17C). Scutellar shield: width to length ratio 1.0; anterolateral edges slightly sinuate; posterior apex pointed. Elytra: upper edge of epipleura with minute serrations.

***Legs*.** Length ratio of metatarsomeres I–V (excluding claws): 100; 66; 61; 47; 122. Claw with ventral apex almost as large as dorsal apex.

***Abdomen*.** Lateral edges of visible abdominal ventrites I–V with minute serrations.

***Male genitalia*** (only apical third observed in current study, see Fig. 17E–G). Apical third robust in ventral and lateral views. Apical third of median lobe in ventral view (Fig. 17E) narrowing from base to apex, apex rounded. Apical third of median lobe in lateral view bent ventrad (Fig. 17G). Apical third of paramere in ventral view: extremely wide, 2–3 × wider than median lobe (measured at base of apical third), sides convex and narrowed to near apex, preapical lateral expansion triangular, facing laterally (Fig. 17F); apex acute beyond preapical lateral expansion, apical mesal callus absent. Apical third of paramere in lateral view: bent ventrad and gradually narrowed towards apex, preapical ventral expansion absent, without hook-shaped structure near apex.

**Female.** Unknown.

###### Type material.

***Holotype***. male (TARI ), labels: 1) Formosa, Y. Miwa; 車路墘, 27/IV, 1926; 2) *Cardiophorusyanagiharae* n. sp., det. Y. Miwa; 3) Type; 4) 1072; 5) Not type?, det. W. Suzuki, 1989; 6) Holotype of *Cardiophorusyanagiharae* Miwa, 1927, Identified by Ruan & Douglas, 2020.

###### Remarks.

Miwa (1927) stated that only one specimen was used for description of *Cardiophorusyanagiharae* Miwa, 1927 in the original publication. Therefore, according to ICZN (Art. 73.1.2.), the single specimen he described was fixed as holotype.

We have investigated the TARI Elateridae collection. Only one single specimen labeled as “*Cardiophorusyanagiharae* sp. nov.” was discovered, which we have identified as the holotype for the following reasons: 1) it is preserved in the type collection with a circular type label and a rectangular TARI  type number label; 2) it has a label indicating “*Phorocardiusyanagiharae* n. sp.” in Miwa’s handwriting; and 3) it has a label that indicates the specimen locality “車路墘”, which is a location in the city “Tainan”, which matches the type locality Miwa (1927) provided.

**Figure 18.**
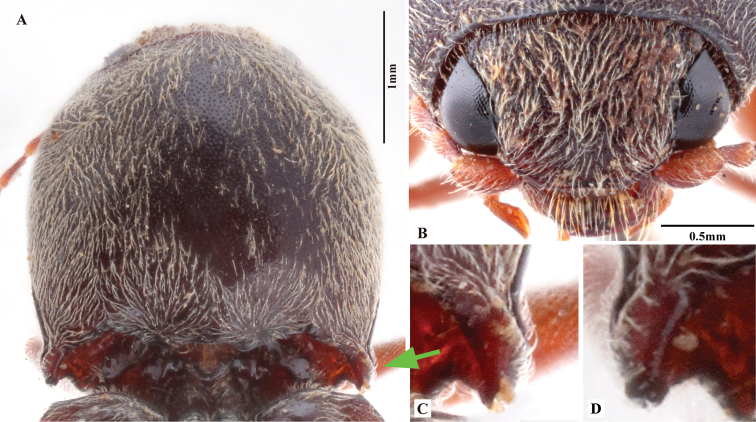
*Phorocardiusyanagiharae* (Miwa, 1927). **A** holotype, pronotum, dorsal view **B** holotype, head, frontal view **C** right posterior angle of pronotum, dorsal view **D** left posterior angle of pronotum, dorsal view.

We believe Miwa had incorrectly reported the sex of the type as female. Moreover, Miwa implied the collecting date is “21/IV, 1926”. However, according to our examination, the date on the label is “27/IV, 1926”.

The previous record of *Phorocardiusyanagiharae* from Sichuan province (Jiang 1993; Jiang and Wang 1999) are erroneous. We investigated those specimens the authors used, and they turned out to be a new species (i.e., *Phorocardiusyunnanensis* Ruan & Douglas, sp. nov., see following text).

Based on specimen information, this species inhabits low elevations (below 100 m) in south Taiwan island. The area is rainy, with subtropical to tropical climate. This species is currently considered endemic to Taiwan.

##### 10. Phorocardius yunnanensis

Taxon classificationAnimaliaColeopteraElateridae

Ruan & Douglas sp. nov.

F997E33F-A32F-5490-997E-2AD787B38A54

http://zoobank.org/66DD489B-D095-4CCE-86A0-40676B3F1DA3

**Figure 19.**
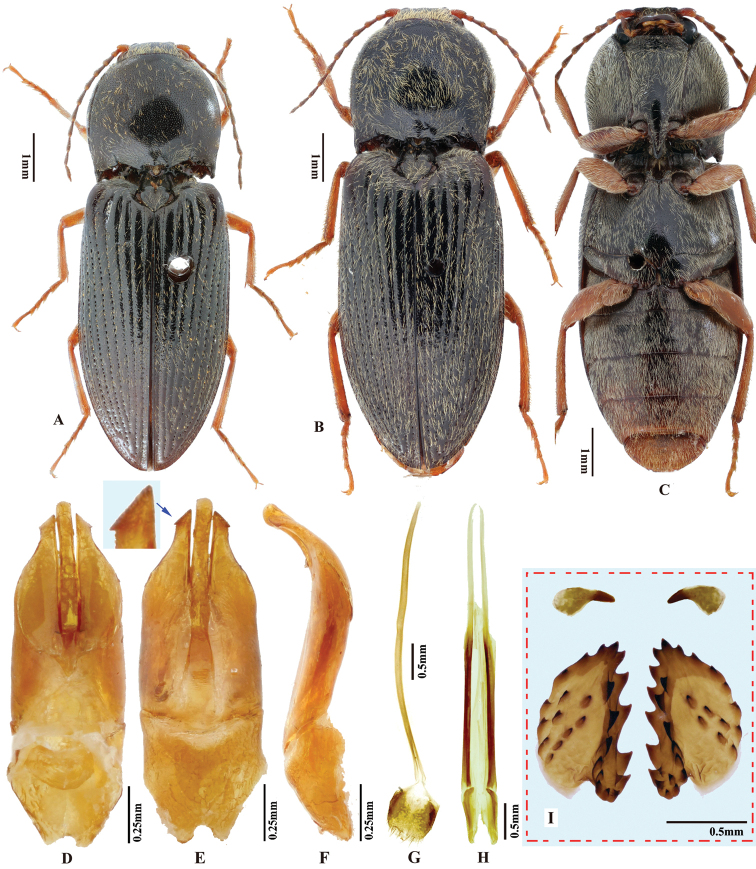
*Phorocardiusyunnanensis* Ruan & Douglas, sp. nov. **A** holotype, male, dorsal view **B** paratype, female, dorsal view **C** paratype, female (Yunnan, Xi-shuan-ban-na, Meng-zhe), ventral view **D** aedeagus of holotype, dorsal view **E** aedeagus of holotype, ventral view, arrow indicating apex of paramere **F** aedeagus of holotype, lateral view **G** female abdominal sternite VIII, dorsal view (paratype) **H** ovipositor of paratype, dorsal view **I** distal (upper side) and proximal sclerites of bursa copulatrix (paratype).

**Figure 20.**
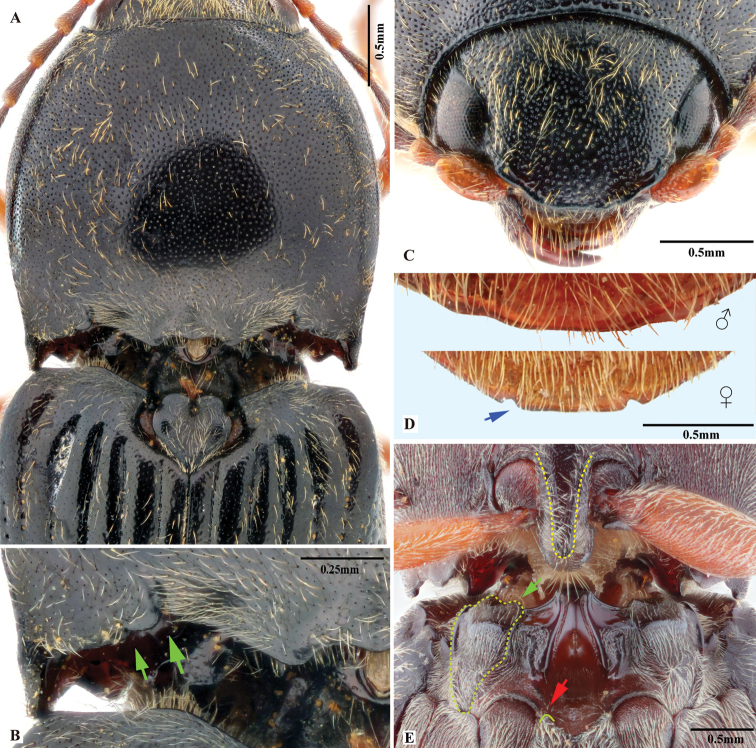
*Phorocardiusyunnanensis* Ruan & Douglas, sp. nov. **A** pronotum and scutellum of holotype, dorsal view **B** posterior edge of pronotum, left side, dorsal view, arrows showing sublateral incisions **C** head of holotype, frontal view **D** apex of last abdominal ventrite (ventrite V) of male and female, ventral view (paratypes), arrow showing apical lateral incisions **E** paratype, ventral view of pro- and mesothorax; indicating shapes of prosternal process, mesepisternum (upper, green arrow on antero-mesal angle) and projections on posterior edge of mesosternum (lower, red arrow).

Figs 19 , 20 , 23I , 24I , 26H

###### Type locality.

Yunnan Prov., Xi-shuang-ban-na, Meng-a (alt. 1050–1080 m).

###### Etymology.

The name of this species refers to the type locality.

###### Distribution.

China (currently endemic to Yunnan).

###### Differential diagnosis.

Body length greater than 7.0 mm; integument black and shiny (non-metallic), elytron without yellow stripes, appendages yellow-brown. Prothorax: procoxal cavities narrowly open; prosternal process not strongly narrowed posterad to ventral apex in ventral view, with apex truncate to slightly rounded. Pterothorax: scutellar shield with posterior apex pointed. Tarsal claw with ventral apex not smaller than dorsal apex. Male genitalia: paramere acute in ventral view with small, acute preapical lateral expansion, without apical mesal callus. Female: apex of last abdominal ventrite (ventrite V) truncate to slightly convex, bent dorsad, each side with an incision.

This species is distinctive for having the dorsum entirely black and shiny and legs entirely yellow-brown (except for brown-black coxae).

*Phorocardiusyunnanensis* Ruan & Douglas, sp. nov. is close to *P.vicinus* in brown-black body color, but distinguishable by the following. In *P.yunnanensis* Ruan & Douglas, sp. nov., pronotum longer with length of pronotum to elytra ratio 0.37–0.40 (excluding posterior angle) or ca. 0.43 (including posterior angle); antennae, palpi of mouthparts and legs yellow-brown to brown; and proximal sclerites of bursa copulatrix with basal edge concave. According to Kollar (1848) and type material (NHMW), *P.vicinus* has length of pronotum to elytra ratio only 0.33; appendages brown-black to black; and proximal sclerites of bursa copulatrix not concave at basal edge.

###### Description.

(based on all type specimens (25♂, 24♀)) Dorsum black and shiny, venter brown-black with last 2–3 ventrites yellow-brown. Antennae brown to yellow-brown, with first two antennomeres slightly lighter in color. Legs entirely yellow-brown (except coxae brown-black). Surface of body with yellow pubescence.

###### Measurements.

(based on all type specimens) Male body length 7.3–8.6 mm, width 2.6–3.3 mm. Female body length 7.3–10.4 mm, width 2.8–3.8 mm. Body length to width ratio 2.6–2.9. Pronotal width to length ratio 1.1–1.2. Pronotal width to body width ratio 0.84–0.91. Elytral length to pronotal length ratio 2.5–2.7; elytron length to width ratio 3.6–3.9.

***Head*.** Frons and vertex with interspaces between punctures 1–2 × average puncture diameter; punctures slightly sparser at centre of vertex. Frontal carina in frontal view straight at middle, curved dorsally at sides. Distance between eyes to width of eye ratio 3.4–4.1. Antenna with apex reaching to or slightly reaching beyond posterior angle of pronotum in male, not reaching to posterior angle in female. Antenna length to body length ratio, in male 0.34–0.39; in female 0.32–0.36. Proportions of antennomere lengths (male): 100 (scape); 57–65; 73–79; 78–80; 77–84; 78–89; 74–89; 77–89; 75–79; 73–83; 105–116.

***Prothorax*.** Pronotum in dorsal view: sides evenly convex from anterior edge to constriction near posterior fifth, widest near mid-length; posterior angles with lateral sides almost straight, not bulged; surface with small punctures, interspaces between punctures 1.5–3 × average puncture diameter. In ventral view, ventral surface of prosternal process with sides carinate and gradually narrow from anterior to posterior end, with apex rounded. In lateral view, prosternal process with ventral surface curved slightly dorsad, posterior end concave (Fig. 24I, upper arrow). Procoxal cavities narrowly open.

***Pterothorax*** (Figs 20E, 24I). Mesepisternum in ventral view with antero-mesal angle broadly rounded mesad of a notch, facing antero-mesally (Fig. 20E, upper (green) arrow); Projections on posterior edge of mesosternum: in ventral view weak (Fig. 20E, lower (red) arrow); in lateral view weak to absent, not produced anteriorly (Fig. 24I, lower (red) arrow). Scutellar shield: width to length ratio 0.7–0.93; anterolateral edges slightly sinuate; posterior edge pointed. Elytra: upper edge of epipleura with minute serrations.

***Legs***. Length ratio of metatarsomeres I–V (excluding claws): 100; 73–79; 65–79; 45–54; 138–154. Claw with ventral apex almost as large as dorsal apex.

***Abdomen***. Lateral edges of visible abdominal ventrites I–V with minute serrations.

***Male genitalia*** (Fig. 19D–F). Robust in dorsal view, slender in lateral view. Median lobe in ventral view narrowing from base to basal third, parallel-sided and slender from basal third to apex, apex broadly rounded to truncate. Median lobe in lateral view evenly curved ventrad from base to apex, apex rounded and dilated. Paramere in dorsal view: wide from base to mid-length, gradually narrowed beyond mid-length, apex slanted; preapical lateral expansion small and sharp, facing laterally; apical mesal callus absent; width 2–2.5 × median lobe width (measured at mid-length of paramere and median lobe respectively). Paramere in lateral view straight from base to mid-length, curved ventrad from mid-length to apex; apex without sharp hook-shaped preapical ventral expansion.

**Female**. Body length slightly larger than male (Fig. 19B, C), apex of abdominal ventrite V in ventral view truncate to slightly convex, bent dorsad, each side with an incision (Figs 20D, 26H, indicated by blue arrow) (in male, apex of abdominal ventrite V not bent dorsad, each side weakly concave, see Fig. 20D). Bursa copulatrix with proximal sclerites large, semi-spherical, base with deep concavity, apex narrowed and angulate; with many spines on internal surface: each with 9–11 large ones on mesal edge, 10–12 smaller ones on disc.

###### Type material.

***Holotype***. ♂ (IZCAS), labels: 1) **Yunnan**, Xi-shuang-ban-na, Meng-a (勐阿), 1050–1080 m, Chinese Academy of Sciences [in Chinese]; 2) 1958.VI.6, leg. Fuji Pu [in Chinese]; 3) Holotype *Phorocardiusyunnanensis* sp. nov. Des. Ruan & Douglas, 2019.

***Paratypes*** (34♂42♀). 1♂1♀ (IZCAS), labels: 1) **Yunnan**, Xi-shuang-ban-na, Meng-a (勐阿), 1050–1080 m, Chinese Academy of Sciences [in Chinese]; 2) 1958.VI.9–10, leg. Shuyong Wang [in Chinese]; 3) Paratype *Phorocardiusyunnanensis* sp. nov. Des. Ruan & Douglas, 2019. • 1♀ (IZCAS), labels: 1) **Yunnan**, Xi-shuang-ban-na, Meng-a (勐阿), 1000 m, Chinese Academy of Sciences [in Chinese]; 2) 1958.V.19, leg. Fuji Pu [in Chinese]; 3) Paratype *Phorocardiusyunnanensis* sp. nov. Des. Ruan & Douglas, 2019. • 2♂3♀ (IZCAS), labels: 1) **Yunnan**, Xi-shuang-ban-na, Meng-zhe (勐遮), 1200 m, Chinese Academy of Sciences [in Chinese]; 2) 1958.VI.15, leg. Fuji Pu [in Chinese]; 3) *Phorocardiusyanagiharae*, det. Siqin Ge; 4) Paratype *Phorocardiusyunnanensis* sp. nov. Des. Ruan & Douglas, 2019. • 5♂6♀ (IZCAS), labels: 1) **Yunnan**, Xi-shuang-ban-na, Meng-zhe (勐遮), 1200 m, Chinese Academy of Sciences [in Chinese]; 2) 1958.VI.15, leg. Shuyong Wang [in Chinese]; 3) *Phorocardiusyanagiharae* (Miwa), det. Shihong Jiang, 1998; 4) Paratype *Phorocardiusyunnanensis* sp. nov. Des. Ruan & Douglas, 2019. • 1♀ (IZCAS), labels: 1) **Yunnan**, Xi-shuang-ban-na, Meng-zhe (勐遮), 1200 m, Chinese Academy of Sciences [in Chinese]; 2) 1958.IV.14, leg. Shuyong Wang [in Chinese]; 3) Paratype *Phorocardiusyunnanensis* sp. nov. Des. Ruan & Douglas, 2019. • 1♂ (IZCAS), labels: 1) **Yunnan**, Xi-shuang-ban-na, Meng-zhe (勐遮), 1200 m, Chinese Academy of Sciences [in Chinese]; 2) 1958.VI.17, leg. Zhi-zi Chen [in Chinese]; 3) Paratype *Phorocardiusyunnanensis* sp. nov. Des. Ruan & Douglas, 2019. • 1♂ (IZCAS), labels: 1) **Yunnan**, Xi-shuang-ban-na, Meng-zhe (勐遮), 870 m, Chinese Academy of Sciences [in Chinese]; 2) 1958.VII.7, leg. Fuji-Pu [in Chinese]; 3) Paratype *Phorocardiusyunnanensis* sp. nov. Des. Ruan & Douglas, 2019. • 1♂ (IZCAS), labels: 1) **Yunnan**, Meng-zhe (勐遮), Nan-nuo-shan, 1100 m, 1957.IV.28, leg. Fuji Pu, light trap [in Chinese]; 2) [same information as label 1, in Russian]; 3) Paratype *Phorocardiusyunnanensis* sp. nov. Des. Ruan & Douglas, 2019. • 1♂ (IZCAS), labels: 1) **Yunnan**, Xi-shuang-ban-na, Meng-hun (勐混), 1200 m, Chinese Academy of Sciences [in Chinese]; 2) 1958.V.28, leg. Shuyong Wang [in Chinese]; 3) Paratype *Phorocardiusyunnanensis* sp. nov. Des. Ruan & Douglas, 2019. • 1♀ (IZCAS), labels: 1) **Yunnan**, Xi-shuang-ban-na, Meng-hun (勐混), 1000–1200 m, Chinese Academy of Sciences [in Chinese]; 2) 1958.V.21, leg. Leyi Zheng [in Chinese]; 3) Paratype *Phorocardiusyunnanensis* sp. nov. Des. Ruan & Douglas, 2019. • 2♂1♀ (IZCAS), labels: 1) **Yunnan**, Xi-shuang-ban-na, Meng-hun (勐混), 1200–1400 m, Chinese Academy of Sciences [in Chinese]; 2) 1958.V.17–24, leg. Xuwu Meng [in Chinese]; 3) Paratype *Phorocardiusyunnanensis* sp. nov. Des. Ruan & Douglas, 2019. • 1♂ (IZCAS), labels: 1) **Yunnan**, Xi-shuang-ban-na, Meng-hun (勐混), 1200 m, Chinese Academy of Sciences [in Chinese]; 2) 1958.V.17, leg. Xuwu Meng [in Chinese]; 3) Paratype *Phorocardiusyunnanensis* sp. nov. Des. Ruan & Douglas, 2019. • 1♂ (IZCAS), labels: 1) **Yunnan**, Xi-shuang-ban-na, Da-meng-long (大勐龙), 650 m, Chinese Academy of Sciences [in Chinese]; 2) 1958.VI.9, leg. Shuyong Wang [in Chinese]; 3) Paratype *Phorocardiusyunnanensis* sp. nov. Des. Ruan & Douglas, 2019. • 1♀ (IZCAS), labels: 1) **Yunnan**, Jing-dong (景东), Dong-jia-fen, 1250 m, 1956.V.27, leg. Zha-gu-liang-ye-fu [in Chinese]; 2) Paratype *Phorocardiusyunnanensis* sp. nov. Des. Ruan & Douglas, 2019. • 1♀ (IZCAS), labels: 1) **Yunnan**, Jing-dong (景东), 1170 m, 1956.VI.30, leg. Zha-gu-liang-ye-fu, light trap [in Chinese]; 2) Paratype *Phorocardiusyunnanensis* sp. nov. Des. Ruan & Douglas, 2019. • 1♂ (IZCAS), labels: 1) **Yunnan**, Jing-dong (景东), Dong-jia-fen, 1250 m, 1956.VI.29, leg. Krizhanovsky [in Chinese]; 2) Paratype *Phorocardiusyunnanensis* sp. nov. Des. Ruan & Douglas, 2019. • 2♀ (IZCAS), labels: 1) **Yunnan**, Jing-dong (景东), Dong-jia-fen, 1250 m, 1956.VI.3, leg. Krizhanovsky [in Chinese]; 2) Paratype *Phorocardiusyunnanensis* sp. nov. Des. Ruan & Douglas, 2019. • 1♂ (IZCAS), labels: 1) **Yunnan**, Bao-shan (保山) to Yong-ping (永平), 1955.V.28, leg. Le Wu [in Chinese]; 2) Paratype *Phorocardiusyunnanensis* sp. nov. Des. Ruan & Douglas, 2019. • 1♂1♀ (IZCAS), labels: 1) **Yunnan**, east of Bao-shan (保山), Lang-cang river 1200 m, 1955.V.28, leg. Bu-xi-ke [in Chinese]; 2) Paratype *Phorocardiusyunnanensis* sp. nov. Des. Ruan & Douglas, 2019. • 2♂ (IZCAS), labels: 1) **Yunnan**, Si-mao (思茅), 1380 m, Chinese Academy of Sciences [in Chinese]; 2) 1958.VI.7, leg. Shuyong Wang [in Chinese]; 3) Paratype *Phorocardiusyunnanensis* sp. nov. Des. Ruan & Douglas, 2019. • 2♂ (IZCAS), labels: 1) **Yunnan**, Si-mao (思茅), Pu-wen, 950–1200 m, 1957.V.11, leg. Guangji Hong & Zhirang Meng [in Chinese]; 2) [same information as label 1, in Russian]; 3) Paratype *Phorocardiusyunnanensis* sp. nov. Des. Ruan & Douglas, 2019. • 3♂12♀ (SZPT, ex. LQCC), labels: 1) **Yunnan**, Pu-er, Si-mao (思茅), 1400 m, 9–11.V.2018, Meizihu Park, leg. Jianyue Qiu & Hao xu, Shenzhen Polytechnic [in Chinese]; 2) Paratype *Phorocardiusyunnanensis* sp. nov. Des. Ruan & Douglas, 2019. • 6♂3♀ (SZPT), labels: 1) **Yunnan**, Lin-cang, Yun-xian County, Man-wang township (漫湾镇), light trap, VI–VIII, 2018, leg. Zichun Xiong, Shenzhen Polytechnic [in Chinese]; 2) Paratype *Phorocardiusyunnanensis* sp. nov. Des. Ruan & Douglas, 2019. • 1♂2♀ (SZPT), labels: 1) Nabang Town, 那邦镇, Yingjiang County, **Yunnan**, 2018-IV-3, 252 m, Lu Qiu Leg.; 2) Paratype *Phorocardiusyunnanensis* sp. nov. Des. Ruan & Douglas, 2019. • 4♀ (IZCAS), labels: 1) **Yunnan**, Cang-yuan (沧源), 750–790 m, Chinese Academy of Sciences [in Chinese]; 2) 1980.V.19–22, leg. Jinwen Shang [in Chinese]; 3) Paratype *Phorocardiusyunnanensis* sp. nov. Des. Ruan & Douglas, 2019. • 1♀ (IZCAS), labels: 1) **Yunnan**, Cang-yuan (沧源), ban-lao, 1100 m, Chinese Academy of Sciences [in Chinese]; 2) 1980.V.18, leg. Hongxing Li [in Chinese]; 3) Paratype *Phorocardiusyunnanensis* sp. nov. Des. Ruan & Douglas, 2019. • 1♂ (IZCAS), labels: 1) **Yunnan**, Lu-xi (潞西), 1250 m, Chinese Academy of Sciences [in Chinese]; 2) 1980.V.18, leg. Jinwen Shang [in Chinese]; 3) Paratype *Phorocardiusyunnanensis* sp. nov. Des. Ruan & Douglas, 2019. • 1♀ (IZCAS), labels: 1) **Yunnan**, Rui-li (瑞丽), 1400 m, 1956.VI.6, leg. Benshou Zhou [in Chinese]; 2) [same information as label 1, in Russian]; 3) Paratype *Phorocardiusyunnanensis* sp. nov. Des. Ruan & Douglas, 2019.

###### Remarks.

Based on specimen information, this species is currently only known from mountainous southwest Yunnan, China. It inhabits mainly middle elevations (ca. 650–1400 m). Southwest Yunnan is rainy and humid, subtropical to tropical with subtropical evergreen broad-leaf forest or tropical rain forest. Specimens were collected by sweep-netting and light traps, indicating both diurnal and nocturnal activity.

##### 11. Phorocardius zhiweii

Taxon classificationAnimaliaColeopteraElateridae

Ruan, Douglas & Qiu sp. nov.

2996AC39-28D7-5A2C-9557-DD0AD04C3F9D

http://zoobank.org/6DFDF68D-2147-4055-8034-243A6FEBEA72

**Figure 21.**
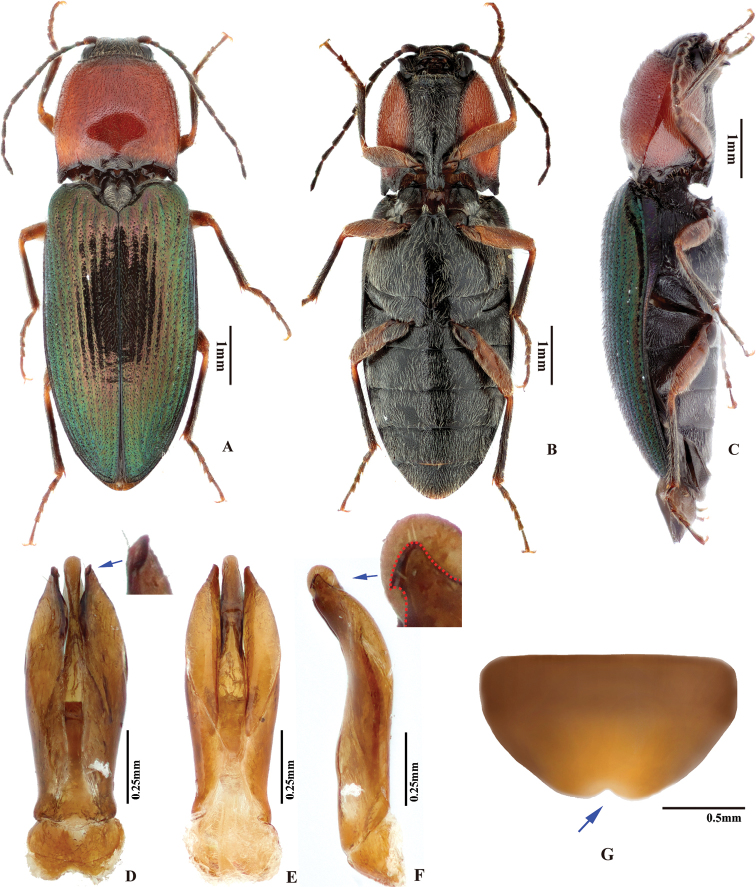
*Phorocardiuszhiweii* Ruan, Douglas & Qiu, sp. nov., holotype, male. **A** habitus, dorsal view. **B** habitus, ventral view **C** habitus, lateral view **D** aedeagus ventral view, arrow indicating apex and apical mesal callus of paramere **E** aedeagus, dorsal view **F** aedeagus, lateral view, arrow indicating apices of paramere and median lobe **G** tergite 7, dorsal view, hand drawing, arrow indicating posterior concavity.

**Figure 22.**
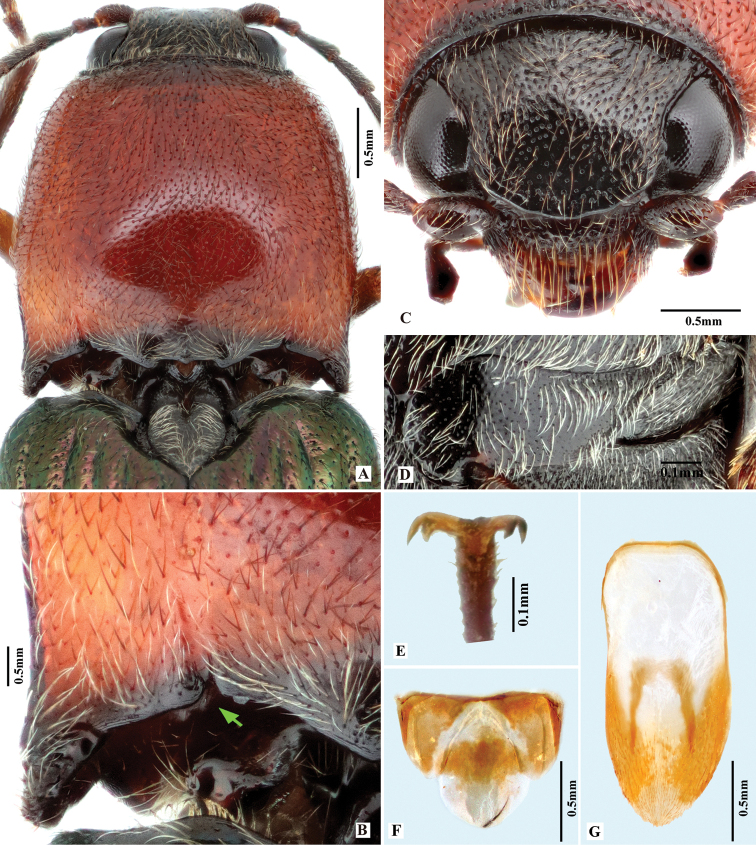
*Phorocardiuszhiweii* Ruan, Douglas & Qiu, sp. nov. **A** pronotum and scutellar shield, dorsal view **B** left posterior edge of pronotum, showing sublateral incision (arrow) **C** head, frontal view **D** metacoxal plate, ventral view **E** tarsal claws **F** male abdominal tergites IX–X, dorsal view **G** male abdominal sternite IX, dorsal view.

**Figure 23.**
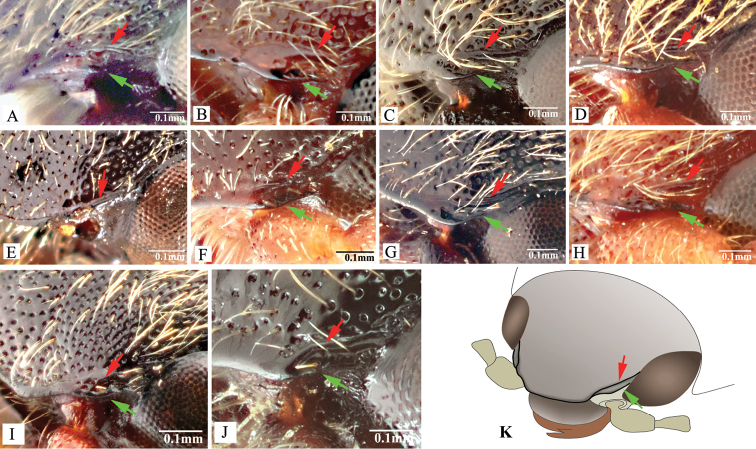
Shape of supraantennal carina of Chinese *Phorocardius species*. Arrows indicating supraantennal carina forked near eye. **A***P.alterlineatus* Ruan & Douglas, sp. nov. **B***P.flavistriolatus* Ruan & Douglas, sp. nov. **C***P.florentini*. **D***P.magnus*. **E***P.manuleatus*. **F***P.minutus* Ruan & Douglas, sp. nov. **G***P.rufiposterus* Ruan & Douglas, sp. nov. **H***P.unguicularis*. **I***P.yunnanensis* Ruan & Douglas, sp. nov. **J***P.zhiweii* Ruan, Douglas & Qiu, sp. nov. **K** head, frontal-lateral view, arrows indicating forked supraantennal carina.

**Figure 24.**
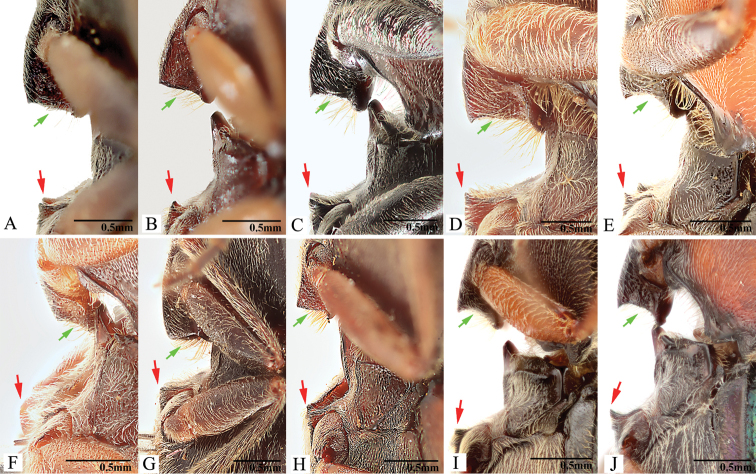
Lateral view of prosternal process and mesosternum of Chinese *Phorocardius* species. Upper green arrows indicating shape of posterior end of prosternal process; lower red arrows indicating projections (absent or present) on posterior edge of mesosternum (i.e., on anteroventral angle of mesosternal fossa according to Douglas 2003). **A***P.alterlineatus* Ruan & Douglas, sp. nov. **B***P.flavistriolatus* Ruan & Douglas, sp. nov. **C***P.florentini*. **D***P.magnus*. **E***P.manuleatus*. **F***P.minutus* Ruan & Douglas, sp. nov. **G***P.rufiposterus* Ruan & Douglas, sp. nov. **H***P.unguicularis*. **I***P.yunnanensis* Ruan & Douglas, sp. nov. **J***P.zhiweii* Ruan, Douglas & Qiu, sp. nov.

**Figure 25.**
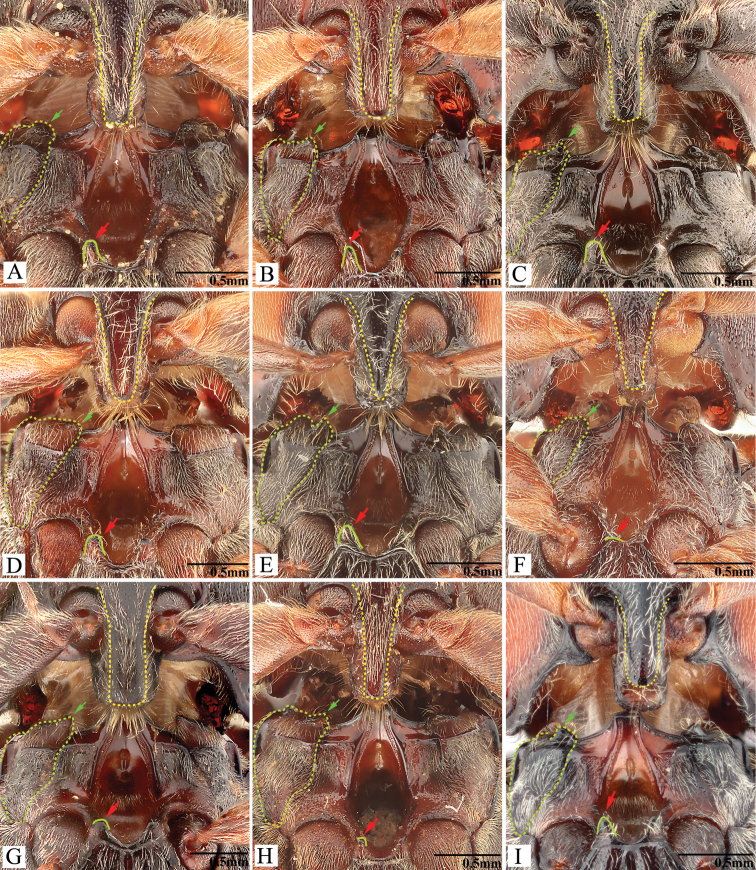
Ventral view of pro- and mesothorax of Chinese *Phorocardius* species. Showing shape of ventral surface of prosternal process (upper, yellow dashed line), mesepisternum (middle, green arrow and dashed line) and projections on posterior edge of mesosternum (lower, red arrow and solid line) middle, green arrows indicating anterior-mesal angle of mesepisternum. **A***P.alterlineatus* Ruan & Douglas, sp. nov. **B***P.flavistriolatus* Ruan & Douglas, sp. nov. **C***P.florentini*. **D***P.magnus*. **E***P.manuleatus*. **F***P.minutus* Ruan & Douglas, sp. nov. **G***P.rufiposterus* Ruan & Douglas, sp. nov. **H***P.unguicularis*. **I***P.zhiweii* Ruan, Douglas & Qiu, sp. nov.

Figs 21 , 22 , 23J , 24J , 25I

###### Type locality.

Yunnan: Long-chuan county, Hu-sa township, Gun-bang-jian-shan [i.e., Bang-gun-jian-shan邦棍尖山].

###### Etymology.

This species is named after its collector, Mr Zhiwei Dong, who generously provided specimens for this study.

###### Distribution.

China (Yunnan).

###### Differential diagnosis.

Body length greater than 7.0 mm; pronotum with integument red, elytra metallic green. Prothorax: procoxal cavities open; prosternal process not strongly narrowed posterad to ventral apex in ventral view, with ventral apex rounded. Pterothorax: scutellar shield with posterior edge pointed. Tarsal claw with ventral apex not smaller than dorsal apex. Male genitalia: paramere without preapical lateral expansion, but with apical mesal callus. Female unknown.

This species is distinct among *Phorocardius* in its entirely metallic green elytra and red pronotum. It is the second species having metallic color on elytra, as the type species of the genus (i.e., *P.florentini* (Fleutiaux, 1895)) also has slight blue metallic luster on elytra.

*Phorocardiuszhiweii* Ruan, Douglas & Qiu, sp. nov. resembles *P.manuleatus* most in body shape and color of leg and prothorax. They can be easily separated by elytral color and aedeagus shape. In *P.zhiweii* Ruan, Douglas & Qiu, sp. nov., elytra are metallic green with slight purple luster; in ventral view, the aedeagus has apical fourth slightly narrowed, robust in both ventral and lateral views; and the paramere has preapical lateral expansion absent, apical mesal callus present; while in *P.manuleatus*, elytra are black to yellow, without metallic color; and the aedeagus in ventral view has the apical fourth greatly narrowed. The aedeagus is slender in both ventral and lateral views with parameres lacking acute preapical lateral expansions or apical mesal calli.

###### Description.

(based on holotype) Dorsum shiny. Head black, with mouthparts red-brown to brown. Antennae brown. Pronotum orange, with posterior edge dark brown (Fig. 22A). Scutellar shield black. Elytra entirely metallic green, with purple luster. Hypomera orange; rest of ventral surface black (Fig. 21B). Epipleura metallic purple. Legs black on coxa, pale brown on femur and basal half of tibia, dark brown from mid-length of tibia to apex. Body with short, yellow-grey pubescence, brown setae also present on disc of pronotum.

###### Measurements.

(based on holotype) Body length 8.0 mm, width 2.8 mm. Body length to width ratio 2.9. Pronotal width to length ratio 1.1. Pronotal width to body width ratio 0.80. Elytral length to pronotal length ratio 2.5; elytron length to width ratio 3.8.

***Head*.** Frons and vertex with interspaces between punctures 1–3 × average puncture diameter (Fig. 22C). Frontal carina in frontal view convex. Antenna with apex extending to basal edge of elytron. Distance between eyes to width of eye ratio 2.7. Antenna length to body length ratio 0.42. Proportions of antennomere lengths (male): 100 (scape); 71; 76; 100; 100; 105; 103; 90; 90; 88; 116.

***Prothorax*.** Pronotum in dorsal view (Fig. 22A): sides slightly convex from anterior edge to concavity near posterior fourth, widest near posterior third; posterior angles with lateral sides almost straight, not bulged; surface with interspaces between punctures 2–2.5 × average puncture diameter. In ventral view, ventral surface of prosternal process with sides carinate and slightly and gradually narrow from anterior to mid-length, parallel from mid-length to near posterior end, apex broadly rounded. In lateral view, prosternal process with ventral surface curved slightly dorsad, posterior end concave (Fig. 24J). Procoxal cavities open.

***Pterothorax*** (Figs 24J, 25I). Mesepisternum in ventral view with antero-mesal corner right-angled (Fig. 25I, upper, green arrow). Projections on posterior edge of mesosternum: in ventral view present (Fig. 25I, red arrow); in lateral view present, strongly developed, produced anteriorly (Fig. 24J, red arrow). Scutellar shield: width to length ratio 1.0; anterolateral edges slightly sinuate; posterior apex pointed. Elytra: upper edge of epipleura with minute serrations.

***Legs*.** Length ratio of metatarsomeres I–V (excluding claws): 100; 71; 53; 50; 120. Claw with ventral apex almost as large as dorsal apex.

***Abdomen*.** Lateral edges of visible abdominal ventrites I–V with minute serrations.

***Male genitalia*.** Robust in ventral and lateral views. Median lobe in ventral view with sides nearly parallel, slightly narrowed near apex; apex broadly rounded (Fig. 21D). Median lobe in lateral view gently bent ventrad, apex dilated and broadly rounded (Fig. 21F). Paramere in ventral view: wide, widest near mid-length, sides convex to near apex; preapical ventral expansion absent, mesal side of apex with ovoid disc-shaped callus (Fig. 21D, indicated by blue arrow); paramere 2–3 × wider than median lobe (measured at mid-length of paramere and median lobe respectively). Paramere in lateral view: robust, almost straight at basal half, bent ventrad at apical half; preapical ventral expansion hook-like, with acute tip facing ventrad (Fig. 21F, indicated by blue arrow).

**Female.** Unknown.

###### Type material.

***Holotype***. male (IZCAS, ex. LQCC), labels: 1) **Yunnan**, Long-chuan county, Hu-sa township (户撒), Mang-dong road, Gun-bang-jian-shan Mt., leg. Zhiwei Dong, 2018-VI-5 [label in Chinese]; 2) ***Holotype***, *Phorocardiuszhiweii* sp. nov., Des. Ruan et al., 2019.

###### Remarks.

This species inhabits middle elevations (around 1500 m) in Yunnan Prov., south China. This area is rainy, subtropical to tropical, with subtropical evergreen broad-leaf forest or tropical rain forest. This species is known only from the Oriental Region.

There are two undetermined female specimens (SZPT) from Xi-shuang-ban-na (西双版纳), Yunnan that have metallic blue elytra and a black pronotum. One male specimen (SZPT) from Ying-jiang (盈江), Yunnan also has metallic blue elytra and a black pronotum. Its aedeagus is extremely close to that of *Phorocardiuszhiweii* Ruan, Douglas & Qiu, sp. nov. However, it is still not entirely clear whether these are conspecific. More specimens should be studied before a reliable determination can be made.

**Figure 26.**
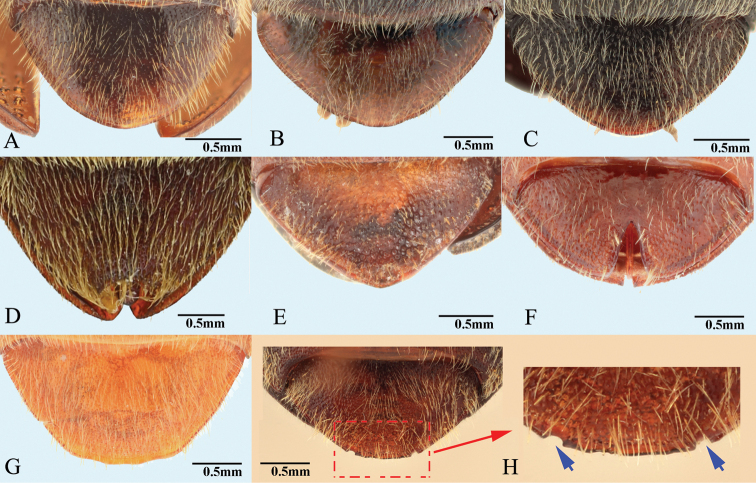
Female abdominal ventrite V in Chinese *Phorocardius* species (ventral view). **A***P.alterlineatus* Ruan & Douglas, sp. nov. **B***P.flavistriolatus* Ruan & Douglas, sp. nov. **C***P.florentini*. **D***P.magnus*. **E***P.manuleatus***F***P.rufiposterus* Ruan & Douglas, sp. nov. **G***P.unguicularis***H***P.yunnanensis* Ruan & Douglas, sp. nov, arrows showing incisions near apex.

**Figure 27.**
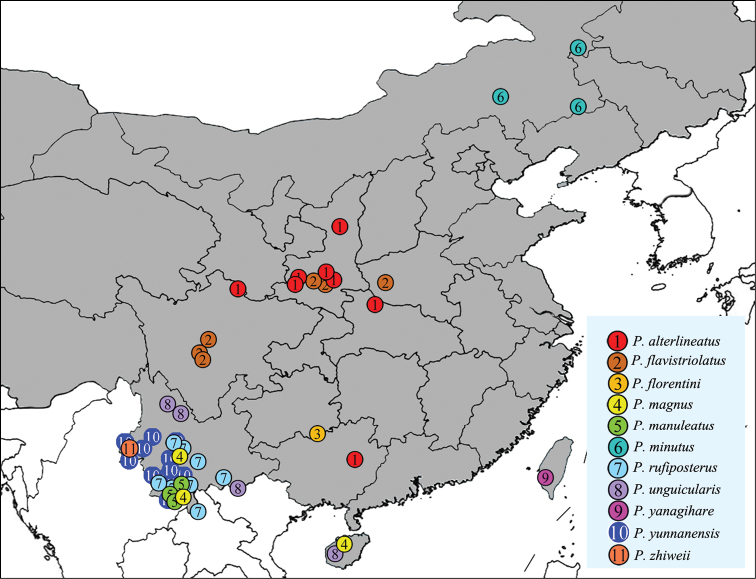
A distribution map of Chinese *Phorocardius* species. **1***P.alterlineatus* Ruan & Douglas, sp. nov. **2***P.flavistriolatus* Ruan & Douglas, sp. nov. **3***P.florentini***4***P.magnus***5***P.manuleatus***6***P.minutus* Ruan & Douglas, sp. nov. **7***P.rufiposterus* Ruan & Douglas, sp. nov. **8***P.unguicularis***9***P.yanagiharae***10***P.yunnanensis* Ruan & Douglas, sp. nov. **11***P.zhiweii* Ruan, Douglas & Qiu, sp. nov.

#### Removal of a questionable distributional record:

##### Phorocardius comptus

Taxon classificationAnimaliaColeopteraElateridae

(Candèze, 1860) [removed from distribution of mainland China and Taiwan]

B1F31EF1-8608-54B8-9E6A-17D78B9A5D99

Fig. 28

Cardiophorus comptus Candèze, 1860: 202. Type locality: Hindoustan méridional, Mysore (interpreted as India, Karnataka, Mysuru). Lectotype designated here.
Phorocardius comptus : Miwa 1934: 209 (distribution).
Dicronychus comptus : Ôhira 1973: 38 (as comb. nov. from Cardiophorus, distribution).
Phorocardius comptus : Ôhira 1978: 96 (as comb. nov. from Dicronychus, distribution, photograph of habitus).
Phorocardius comptus : Cate et al. 2007: 206 (distribution).

###### Distribution.

India (Candèze 1860, 1891); Nepal (Ôhira 1978); Sri Lanka (Ôhira 1973). Excluded from China here.

###### Remarks.

**Figure 28.**
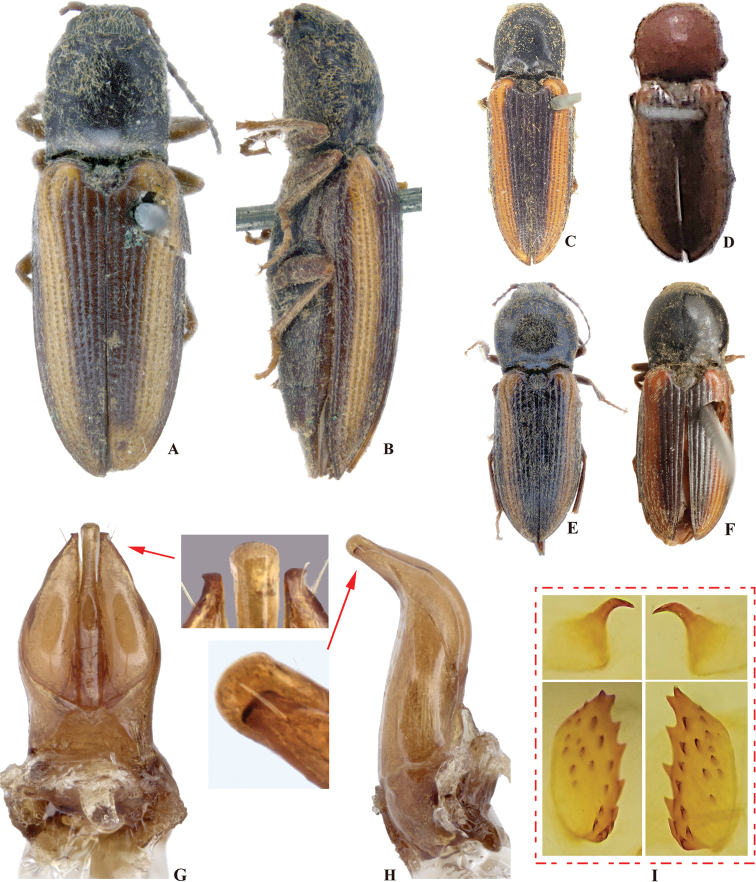
*Phorocardiuscomptus* (Candèze, 1860) (insets **A, B, E, G–I** are provided by Ms Karine Savard, Agriculture and Agri-food Canada; insets **C, D** are provided by Dr Yijie Tong, IZCAS) . **A** lectotype, male, dorsal view **B** lectotype, male, lateral view **C** paralectotype, male, dorsal view **D** paralectotype, female, dorsal view **E** female, dorsal view (NHMUK, non-type specimen) **F** female, dorsal view (TARI , used for distributional record by Miwa (1927)) **G** aedeagus of lectotype, dorsal view, arrow indicating apices of parameres and median lobe **H** aedeagus of lectotype, lateral view, arrow indicating apices of parameres and median lobe **I** distal (upper side) and proximal sclerites of bursa copulatrix (paralectotype, habitus shown in **D**).

*Phorocardiuscomptus* was recorded from Taiwan by Miwa (1927, 1931). However, Suzuki (1999) considered the record questionable. He excluded *P.comptus* from Taiwan after studying the specimens that Miwa (1927) used for the record. The same specimen was examined in our study (TARI , see Fig. 28F). We agree with Suzuki (1999) that the specimen used by Miwa (1927) is one of the “Shiraki specimens”. “Shiraki specimens” were originally housed in NHMUK, collected from tropical Oriental countries (e.g., India, Nepal, Borneo, etc.) and shipped to Taiwan by Dr Shiraki in 1916. All specimen labels were then replaced by new labels with several specific localities from Taiwan such as “Rônô”, “Kôshun”, “Kôtoshô”, “Horisha”, “Hori”, “Musha”, etc. (Kurosawa 1980; Chu and Xiao 1981; Chu 2011, 2013). Most of these Taiwan locality names refer to localities in the south and southeast Asia, the codes for these true locality names were documented in a file housed in National Taiwan University, Taipei, which was already lost shortly after world war II (Chu 2011). Additionally, under each of these specimens, there is a typical Shiraki’s label with his handwriting and a red circle mark. Therefore, the single *P.comptus* specimen in TARI  is one of these “Shiraki specimens”, since it has a locality label indicating “Rônô”, and a second label with Shiraki’s handwriting and a red circle mark.

*Phorocardiuscomptus* was previously recorded by Jiang (1993) and Jiang and Wang (1999) in Hubei Prov., China. We examined the specimens used in those studies and found that these specimens are not conspecific with *P.comptus*, instead they are described as a new species in this study (i.e., *Phorocardiusalterlineatus* Ruan & Douglas sp. nov. above). We consider *P.comptus* to be absent from mainland China and Taiwan.

**Figure 29.**
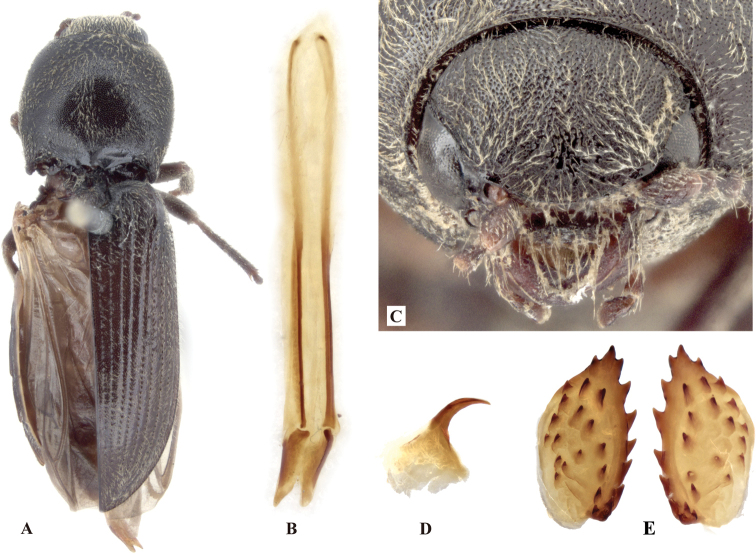
*Phorocardiuscontemptus* (Candèze, 1860) (images are provided by Ms Karine Savard, Agriculture and Agri-food Canada). **A** lectotype, female, dorsal view **B** ovipositor, dorsal view **C** head, frontal view (lectotype) **D** distal sclerite of bursa copulatrix (lectotype; only one sclerite is shown) **E** proximal sclerites of bursa copulatrix (lectotype).

Candèze (1860: 202) described *Cardiophoruscomptus* and *C.contemptus* on the same page and stated that *C.contemptus* (see Fig. 29) may be a variety *C.comptus*. The examination of the type material of both species shows that they are similar in female genitalia and external characters (except for the elytral color). It is possible that *C.contemptus* is a junior synonym of *C.comptus*. However, as we have not studied any male specimen of *C.contemptus*, it is treated here as valid.

Three specimens (NHMUK) are designated as lectotype or paralectotypes for the following reasons: 1) they are labeled with “SYNTYPE”; 2) the labels indicate either “S. India” or “Hindoustan”, which are consistent with the original description; 3) the collection of Candèze before 1869 had been transferred to the NHMUK (Bousquet 2016); and 4) the type materials are absent in either RBINS or MNHN.

###### Type material.

***Lectotype*** of *Cardiophoruscomptus* Candèze, 1860: ♂ (NHMUK), labels: 1) Syntype [a circular label]; 2) 677; 3) [a small blank square label in deep-red color]; 4) *Cardioph.Comptus* Cand. Hindoustan; 5) Janson Coll. Ex. Deyrolle. 1903.130; 6) Lectotype, *Cardiophoruscomptus* Candèze, 1888, Des. Ruan & Douglas, 2020.

***Paralectotypes*** of *Cardiophoruscomptus* Candèze, 1860: 1♂ (NHMUK), labels: 1) SYNTYPE [a circular label]; 2) S. India; 3) 733; 4) 11; 5) Janson coll., ex Dejean. 1903-130; 6) *C.comptus* ♂; 7) Paralectotype, *Cardiophoruscomptus* Candèze, 1888, Des. Ruan & Douglas, 2020. 1♀ (NHMUK), labels: 1) S. India, ex coll. …… [characters illegible]; 2) Janson coll., ex Dejean. 1903-130; 3) 727; 4) 10; 5) *C.Comptus* …… [characters illegible], ♀ Cdzé.; 6) ♀ genitalia See slide Coll. No. 796; 7) Paralectotype, *Cardiophoruscomptus* Candèze, 1888, Des. Ruan & Douglas, 2020. [We consider this female specimen as one of the paralectotypes because the locality on the label is consistent with the original description, and the specimen has a unique red prothorax, which is described as a variation by the author (Candèze 1860). This specimen is shown in Fig. 28D].

###### Additional material.

The specimen that was used as a distributional record by Miwa (1927) [this specimen is actually from the south or southeast Asia instead of Taiwan as discussed above]: 1♀ (TARI ), labels: 1) Rônô; 2) *Phorocardiuscomptus* Cand. Det T. Shiraki; 3) Not Taiwan, Shiraki specimen, W. Suzuki, 1989.

The specimens that were misidentified as *P.comptus* and used as a distributional record by Jiang (1993) and Jiang and Wang (1999) [these specimens are identified as *P.alterlineatus* sp. nov. Ruan & Douglas in this study]: 1♂ (SZPT), labels: 1) Hubei, Wu-dang Mts., Chao-tian-gong (朝天宫), 1982.VII.5; 2) *Phorocardiuscomptus* (Candèze), det. Shihong Jiang 19; 3) 7.30*2.60 cm. • 1♂ (SZPT), labels: 1) Hubei, Wu-dang Mts., Zi-xiao (紫霄), 1982.VII.10; 2) *Phorocardiuscomptus* (Candèze), det. Shihong Jiang 1993. • 1♀ (SZPT), labels: 1) Hubei, Wu-dang Mts., Jin-ding (金顶), 1982.VII.9; 2) *Phorocardiuscomptus* (Candèze), det. Shihong Jiang 1993. • 1♀ (SZPT), labels: 1) Hubei, Wu-dang Mts., Lao-yan (老燕), 1983.VII.2; 2) *Phorocardiuscomptus* (Candèze), det. Shihong Jiang 1993. • 1♀ (SZPT), labels: 1) Hubei, Wu-dang Mts., Nan-yan (南岩), 1983.VII.2; 2) *Phorocardiuscomptus* (Candèze), det. Shihong Jiang 1991.

## Discussion

No new *Phorocardius* species were described between 1931 and 2015, and only three *Phorocardius* species were documented from China since 1931. Our study shows that the species richness of *Phorocardius* is greater than previously known, particularly in China. Southwest China (especially the Heng-duan Mountains) is renowned as a global biodiversity hot spot (Myers et al. 2000). The high diversity of that area is most evident in that five of the six new species described in this study were discovered in this area (although other areas also require increased collecting effort).

The northernmost specimens of *Phorocardius* are of *P.minutus* Ruan & Douglas, sp. nov., collected from Ke-you-zhong-qi, Inner Mongolia, with minimum January temperatures near –20 °C. If monophyletic with other *Phorocardius*, this shows that *Phorocardius* species can inhabit not only humid tropical rain forest, but also arid and freezing grassland areas. This finding also suggests that at least some species of *Phorocardius* may not require forest soils, hollow trees, or decaying wood for larval development. The discovery of *P.minutus* expands the collective distributional records for the genus *Phorocardius* deep into the Palearctic Region for the first time.

According to specimen records, the highest elevation for Chinese *Phorocardius* species is 2750 m at the foot of Yu-long-shan Mountain (also know as Yu-long Snow Mountain), with snow present year-round above 3500 m, while the lowest elevation is around 50 m. The strongly sclerotized ovipositors of all *Phorocardius* species examined here suggests that they can oviposit in dense substrates, such as soils that are not sandy.

*Phorocardiuszhiweii* is the second species described in the genus with metallic coloration on the elytra, with *P.florentini* also having a slight blue metallic luster on the elytra. There may be additional undescribed metallic species, as we have examined three other undetermined specimens with metallic blue elytra.

We found that using the open procoxal cavities as a generic character, as in Douglas (2017), for *Phorocardius* is incorrect because the procoxal cavities of *P.rufiposterus* are closed (Fig. 15E), only narrowly open in rare cases. The degree of the opening of procoxal cavities also slightly varied between individuals of *P.magnus* Fleutiaux, 1931, with many specimens have nearly closed (narrowly open) procoxal cavities. Douglas (2017) also incorrectly placed *Phorocardius* in the key to genera among species with the pronotal lateral carina present in the first half of couplet 4. But the proximal sclerites of the bursa copulatrix are ovoid as mentioned there. Users of that key might incorrectly identify *Phorocardius* specimens as *Ryukyucardiophorus* based on the pronotal character. However, *Phorocardius* can be distinguished from *Ryukyucardiophorus* by its apically split claws (tooth at base in *Ryukyucardiophorus*) and by having four sclerites in the bursa copulatrix (only two in *Ryukyucardiophorus*).

The shape of the male genitalia illustrated in this study is highly variable in some species, especially in *P.flavistriolatus*, *P.manuleatus* and *P.minutus*. This may be due to one or more of the following reasons:

1. Rotation of structures. The variations could be amplified in planar illustrations (photographs only show two dimensions of three-dimensional structures. For example, in P. flavistriolatus and P. manuleatus, the apical mesal part of the paramere is bent (or turned) ventrad, and a slight bending (or turning) can cause a substantial difference from ventral or dorsal views (shown in Fig. 6).
2. Variation in degree of sclerotization. Some individuals have less strongly sclerotized aedeagi than other individuals. This can result in slightly different shapes. An extreme case was observed in one specimen of P. minutus, whose aedeagus had one paramere normal, and the other slightly collapsed and deformed.
3. Mating activity. In a few individuals of P. flavistriolatus (e.g., Fig. 4J), the apical part of the parameres are much more divergent than those in other individuals. This is probably due to the aedeagi being extended at the time of death of the beetle because divergent parameres were found only in specimens preserved with the aedeagus entirely extended from the posterior end of specimens. In Cardiophorinae (and probably Negastriinae), when the aedeagus is extended before copulation, the distal part of parameres diverge from the median lobe (the base of parameres are fused, with the apex flexible, Iablokoff-Khnzorian and Mardjanian 1981; Douglas 2017). We also observed the copulation in Ludioschema obscuripes for comparison. During the copulation, the distal part of the parameres diverges from the median lobe. After copulation, the aedeagus starts to retract back into the body, the parameres gradually move close to median lobe, and when the aedeagus is almost entirely retracted, the parameres return closely to median lobe.
4. Undescribed species diversity. It also remains possible that these species definitions contain undescribed cryptic species, so that further DNA comparisons should be done to test species limits.

This study also first documents that the shape of the apex of abdominal ventrite V in females is highly variable (arcuate, with arcuate median indentation, or with elongate invagination containing slender blade-like projection) between species (see Fig. 26A–H). This character system (first noted in unpublished drawings in specimen drawers in NHMUK by Christine von Hayek) provides a set of powerful diagnostic characters for some species (e.g., *P.rufiposterus*, *P.magnus*, and *P.yunnanensis*). However, in males, the same structure (usually arcuate and simple) is more evenly convex and lacks interspecific variation.

## Supplementary Material

XML Treatment for Phorocardius

XML Treatment for Phorocardius alterlineatus

XML Treatment for Phorocardius flavistriolatus

XML Treatment for Phorocardius florentini

XML Treatment for Phorocardius magnus

XML Treatment for Phorocardius manuleatus

XML Treatment for Phorocardius minutus

XML Treatment for Phorocardius rufiposterus

XML Treatment for Phorocardius unguicularis

XML Treatment for Phorocardius yanagiharae

XML Treatment for Phorocardius yunnanensis

XML Treatment for Phorocardius zhiweii

XML Treatment for Phorocardius comptus
